# Insights into Adaptations to a Near-Obligate Nematode Endoparasitic Lifestyle from the Finished Genome of *Drechmeria coniospora*

**DOI:** 10.1038/srep23122

**Published:** 2016-03-15

**Authors:** Liwen Zhang, Zhengfu Zhou, Qiannan Guo, Like Fokkens, Márton Miskei, István Pócsi, Wei Zhang, Ming Chen, Lei Wang, Yamin Sun, Bruno G. G. Donzelli, Donna M. Gibson, David R. Nelson, Jian-Guang Luo, Martijn Rep, Hang Liu, Shengnan Yang, Jing Wang, Stuart B. Krasnoff, Yuquan Xu, István Molnár, Min Lin

**Affiliations:** 1 Biotechnology Research Institute, Chinese Academy of Agricultural Sciences, Beijing, China; 2 Key Laboratory of Agricultural Genomics (Beijing), Ministry of Agriculture, China; 3 Molecular Plant Pathology, University of Amsterdam, Amsterdam, the Netherlands; 4 Department of Biotechnology and Microbiology, Faculty of Science and Technology, University of Debrecen, Hungary; 5 Department of Biochemistry and Molecular Biology, University of Debrecen, Debrecen, Hungary; 6 Tianjin Key Laboratory of Microbial Functional Genomics, TEDA School of Biological Sciences and Biotechnology, Nankai University, Tianjin, China; 7 Plant Pathology & Plant-Microbe Biology, Cornell University, Ithaca, New York, USA; 8 USDA-ARS, Robert W. Holley Center for Agriculture and Health, Ithaca, New York, USA; 9 Department of Microbiology, Immunology and Biochemistry, University of Tennessee Health Science Center, Memphis, Tennessee, USA; 10 State Key Laboratory of Natural Medicines, Department of Natural Medicinal Chemistry, China Pharmaceutical University, Nanjing, China; 11 Natural Products Center, School of Natural Resources and the Environment, University of Arizona, Tucson, Arizona, USA

## Abstract

Nematophagous fungi employ three distinct predatory strategies: nematode trapping, parasitism of females and eggs, and endoparasitism. While endoparasites play key roles in controlling nematode populations in nature, their application for integrated pest management is hindered by the limited understanding of their biology. We present a comparative analysis of a high quality finished genome assembly of *Drechmeria coniospora*, a model endoparasitic nematophagous fungus, integrated with a transcriptomic study. Adaptation of *D. coniospora* to its almost completely obligate endoparasitic lifestyle led to the simplification of many orthologous gene families involved in the saprophytic trophic mode, while maintaining orthologs of most known fungal pathogen-host interaction proteins, stress response circuits and putative effectors of the small secreted protein type. The need to adhere to and penetrate the host cuticle led to a selective radiation of surface proteins and hydrolytic enzymes. Although the endoparasite has a simplified secondary metabolome, it produces a novel peptaibiotic family that shows antibacterial, antifungal and nematicidal activities. Our analyses emphasize the basic malleability of the *D. coniospora* genome: loss of genes advantageous for the saprophytic lifestyle; modulation of elements that its cohort species utilize for entomopathogenesis; and expansion of protein families necessary for the nematode endoparasitic lifestyle.

Although annual crop losses to plant-parasitic nematodes are estimated at a staggering $157 billion worldwide^1^, options for nematode pest management are very limited due to environmental safety concerns^2^. This situation demands further research to discover effective but environmentally responsible alternatives to replace legislatively withdrawn nematicides. Biological control agents, such as nematophagous fungi, may be part of the answer when applied in the context of integrated pest management systems^3^,^4^. Thus, understanding the mechanisms governing the interactions between nematophagous fungi and their nematode prey, and biocontrol strategies based on these interactions are key issues for crop protection.

Nematophagous fungi comprise over 200 species from all major fungal taxa^5^. Most of these fungi are facultative parasites^4^, with the nematode prey serving as a supplementary nitrogen and lipid source for a basically saprophytic lifestyle^5^. Nematophagous fungi produce various infection structures, and follow three main strategies to parasitize and kill their prey. First, nematode-trapping fungi capture their prey using various trapping devices with mechanical or adhesive functions. Next, female and egg parasites utilize appressoria to penetrate the eggshell or the cyst wall. Finally, endoparasites infect juvenile or adult nematodes using conidia that are ingested by their host, e.g. *Harposporium* spp., or by spores that adhere to the cuticle of the host, e.g. *Drechmeria coniospora* and *Hirsutella minnesotensis*^5^,^6^,^7^. The majority of the endoparasites has a low saprotrophic capacity^6^ and develops more intimate relationships with their hosts, approaching obligate parasitism. Although these fungi may play key roles in controlling the populations of certain nematodes in nature, most research efforts have concentrated on the nematode-trapping fungi and the female and egg parasites.

The ascomycete *D. coniospora* is the sole formally recognized species in the *Drechmeria* genus. It infects a variety of nematode species, including important plant pathogens such as the potato rot nematode (*Ditylenchus destructor*) and the root-knot nematodes (*Meloidogyne* spp.)^5^,^6^. The infection complex of *D. coniospora* and *Caenorhabditis elegans* has also served as a model to examine innate immunity^8^. *D. coniospora* is almost exclusively reliant on its nematode host for survival, and its very poor growth and sporulation on common laboratory media significantly hindered microbiological and genetic research on this organism, as compared to other endoparasites such as *H. minnesotensis*^7^. Nevertheless, pioneering studies of Jansson, Dijksterhuis and others^6^,^9^,^10^,^11^,^12^,^13^,^14^,^15^,^16^, and recent 3D imaging by Rouger *et al.*^17^ clarified the infection cycle of *D. coniospora* (Fig. 1).

In recent years, -omics studies have significantly improved our understanding of host-microbe interactions, especially in those cases where the microorganisms are difficult to grow under laboratory conditions. Sequencing of the genomes of the female and egg parasite *Pochonia chlamydosporia*^18^, the nematode trapping fungi *Arthrobotrys oligospora*^19^, *Monacrosporium haptotylum*^20^, and *Drechslerella stenobrocha*^21^, and the facultative nematode endoparasite *H. minnesotensis* contributed to our understanding of the evolutionarily distinct strategies of nematode pathogenesis. The current study adds to this picture by investigating endoparasitism, the third major nematophagous strategy. Thus, we analyze the completed genome sequence of the near-obligate nematode endoparasite *D. coniospora*, and compare it to the recently published genome sequence of the facultative nematode endoparasite *H. minnesotensis*^7^. Our results shed light on the adaptations brought about by the near-obligate endoparasitic lifestyle of *D. coniospora*, and also highlight dynamic adaptations of the transcriptome to different developmental stages in the fungal life cycle.

## Results and Discussion

### Finished sequence assembly reveals chromosome structure

**Figure 2:**
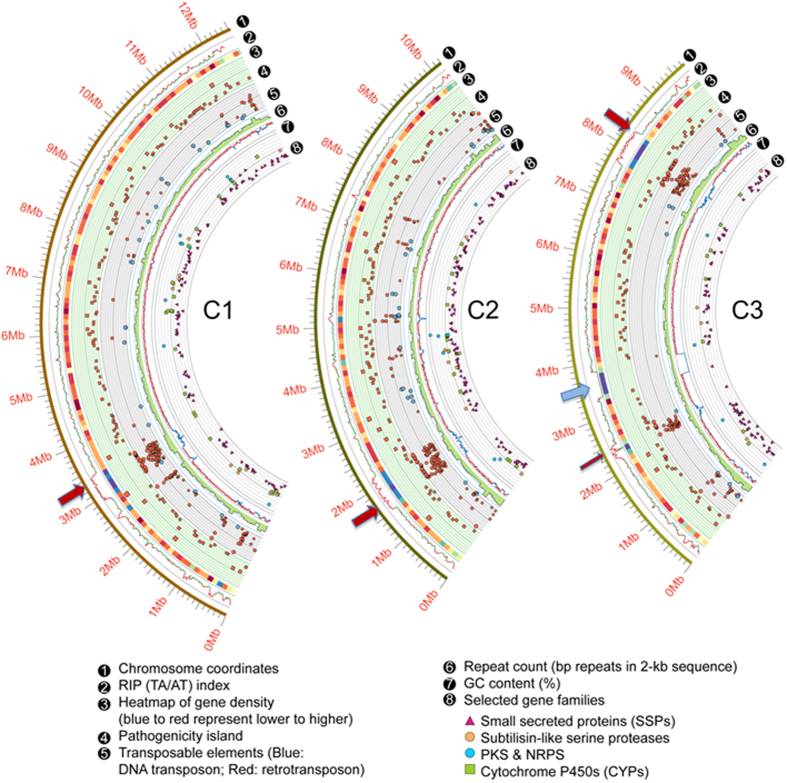
Genome structure of *D. coniospora*. Low gene density and low GC content (arcs 3 and 7, respectively) mark the position of the centromeres (red arrows) and the rDNA repeat region (blue arrow). A vestigial centromere from a putative chromosomal fusion event is indicated on chromosome 3 (narrow red arrow). Repeat induced point mutations (RIP) were quantified using the TpA/ApT index over a 2-kb sliding window. Active RIP is indicated by the index exceeding 0.89.

The 32.5 Mb finished genome assembly of the nematophagous endoparasitic fungus, *D. coniospora* ARSEF 6962, was constructed using a combination of whole-genome shotgun approaches on Solexa, Roche 454 and PacBio RS II platforms, followed by optical mapping (Table S1). Sequence coverage reached 457.9-fold, with a long-contig continuity (N50: 4.14 Mb) that is amongst the highest in published fungal genomic studies (Table 1and S2). Optical mapping anchored and oriented all contigs within three inferred chromosomes, measuring 12.5 Mb, 10.2 Mb and 9.8 Mb, respectively. These inferred chromosomes feature acrocentric regional centromeres marked by high repeat content, reduced gene density and low GC content (Fig. 2). Chromosome III also contains an additional, shorter and less well-defined centromere-like region. Such dicentric chromosomes are presumed to result from chromosome fusions, with the activity of one centromere suppressed during cell division^22^. Chromosome fusion might also account for the unusually low number of inferred chromosomes in *D. coniospora*. Each chromosome is flanked by large regions (approximately 0.5 Mb each) containing species-specific repeats, including the telomere regions. Sequencing of such dense repeats is considered to be extremely challenging, thus the successful mapping of these regions reflects the high quality of our genome assembly. Chromosome III also includes a >500 kb region consisting of tandem repeats of rDNA gene clusters (6-7 kb each), detected by optical mapping (Fig. 2). Similar assemblages have also been found in the genomes of plants and the yeast *Saccharomyces cerevisiae*^23^.

Long-range synteny is evident between the genome sequences of *D. coniospora* and the closely related insect pathogen, *Tolypocladium inflatum* (Fig. S1). 646 large syntenic blocks were detected, comprising 28.6 Mb (87.2%) of the *D. coniospora* genome, and the large majority of the 194 contigs of the *T. inflatum* genome assembly^24^ may be oriented using the chromosomes of *D. coniospora* as a reference. This high level of synteny may indicate that the evolutionary divergence of *D. coniospora* and *T. inflatum* involved the adaptation of common, ancestral pathogenicity processes and mechanisms to their respective nematophagous or entomopathogenic lifestyles.

### Genome dynamics

The completed genome assembly of *D. coniospora* features a repeat sequence content of 12.5% (4.11 Mb), 74% of which is specific to this fungus (Figs 2 and S2, Table S3, Supplementary Results). Transposons comprise 2.2% of the genome, with Type I retrotransposons dominating over Type II DNA transposons (618 vs. 113, respectively, Table S3). Retrotransposons are enriched in the centromeres and the terminal regions of the chromosomes, while DNA transposons appear scattered along the chromosomes (Figs 2 and S2). Similar trends were also observed in *H. minnesotensis*, although with a much higher overall transposon content (32% of the genome)^7^.

The completed genome of *D. coniospora* shows clear evidence for an active repeat-induced point mutation (RIP) system (Figs S3 and S4, Supplementary Results). RIP may be important to limit the activity of transposons in *D. coniospora*, given the frequent co-localization of RIP signals with transposons (Fig. 2, Supplementary Results). RIP only operates during sexual reproduction; its existence together with an active late sexual development protein (DCS 00280) suggests a possible cryptic sexual cycle in *D. coniospora*. The *D. coniospora* genome also encodes a well-conserved MAT1-1-1 ortholog (DCS 00888) while missing a *MAT1-2* idiomorph, suggesting that *D. coniospora* may be heterothallic (Supplementary Results). This is in contrast to *Ophiocordiceps sinensis*^25^ which is homothallic, but in accord with most closely related insect pathogens such as *T. inflatum*^24^, *Metarhizium spp.*^26^, and *Beauveria bassiana*^27^, and perhaps *H. minnesotensis*^7^. Nevertheless, a sexual cycle has never been observed for *D. coniospora* in nature or in the laboratory, nor has a teleomorph been linked to this fungus. This may simply be due to the slow growth rate of the fungus that might preclude easy detection of a sexual stage. Interestingly, the genome of *D. coniospora* encodes only three heterokaryon incompatibility proteins, as opposed to more than 21 present in the facultative insect pathogens *Metarhizium* spp. and *B. bassiana*^25^, and 17 in the facultative nematode endoparasite *H. minnesotensis*^7^. Heterokaryon incompatibility proteins are barriers against vegetative fusions between genetically distinct individuals^28^. The limited diversity of these proteins, as well as the lack of an observed sexual stage suggests that encounters between different fungal individuals are rare for *D. coniospora* (and *O. sinensis*^25^) due to their adaptation to a more specialized, near-obligate endoparasitic lifestyle, and this might also result in a gradual loss of sexual reproduction.

On the other hand, copious production of asexual spores is crucial for the pathogenic cycle of *D. coniospora*. Exhaustive searches for conidiogenesis-related genes in the genome of *D. coniospora* reaffirm the phenotypic observation that the development of conidiferous pegs and those for the formation, maturation and release of conidia in *D. coniospora* is similar to those by the fusaria^29^,^30^,^31^, but quite different from the complex phialide-bearing structures typically observed in the aspergilli (Table S5, Supplementary Results).

### Transcriptome

Since the growth of *D. coniospora* is exceedingly slow on standard media (several months on MEA or CMA)^14^,^15^, we used a specialized agar medium rich in proteins and lipids (liver and kidney medium, see Materials and Methods) to provide sufficient quantities of viable material for transcriptomic analyses, conducted with combined triplicate samples each for the mycelial, early conidiogenesis, and conidia growth phases. A nematode infection transcriptome was also recorded on *C. elegans* as a host by combining daily samples over eight days post-infection, since the low conidial production of *D. coniospora* on lab media precluded more extensive time-scape sampling. Gene expression trends observed in RNAseq for select test genes were validated by quantitative real-time PCR (qRT-PCR), and the transcriptomic data were used to complement and curate gene predictions in the genome. Although proper comparison of infective growth on a host vs. saprophytic growth on artificial media was difficult for *D. coniospora* due to its near-obligate endoparasitic lifestyle, the transcriptome datasets still showed that expression of the *D. coniospora* genome is highly dynamic and reflects the constraints and demands of the given life stage, as discussed in the following sections. Approximately 9% of the genes (862) were differentially expressed (defined as larger than four-fold change in expression between at least two of the three *in vitro* growth stages and p-value < 0.05), with genes involved in carbohydrate, lipid and amino acid transport/metabolism, defense mechanisms, secondary metabolism, and translation, ribosomal structure and biogenesis showing the largest plasticity in the different life stages (Table S6).

### Global evolutionary and functional analysis of predicted genes

#### Phylogeny

**Figure 3:**
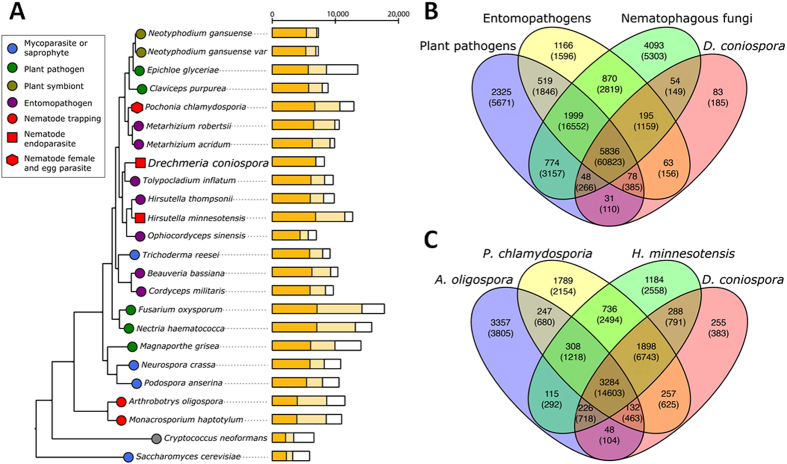
Global comparisons of the deduced proteome of *D. coniospora.* (**A**) Phylogenomic analysis of 24 fungi with varied lifestyles. Different life-strategies are indicated by colored symbols. Dark-yellow bars: number of proteins with orthologs in *D. coniospora;* light-yellow bars: number of proteins with orthologs in species other than *D. coniospora*; white bars: number of proteins with no orthologs. (**B**) Venn diagram showing orthologous groups shared between *D. coniospora* and fungi representing three selected life-strategies. Plant pathogens: *Nectria haematococca*, *F. oxysporum*, and *Claviceps purpurea*; entomopathogens: *T. inflatum, M. robertsii,* and *B. bassiana*; nematophagous fungi: *A. oligospora*, *P. chlamydosporia*, and *H. minnesotensis*. Numbers: count of orthologous protein groups. Numbers in parentheses: counts of proteins. (**C**) Venn diagram showing orthologous groups shared between the near-obligate nematode endoparasite *D. coniospora* with nematophagous fungi representing various infection strategies. *A. oligospora*: nematode trapping fungus; *P. chlamydosporia*: nematode female and egg parasite; and *H. minnesotensis*: facultative nematode endoparasite.

We compared the predicted proteome of *D. coniospora* with those of 23 fungi representing various life-strategies: saprophytes and mycoparisites, plant pathogens or symbionts, entomopathogens, and nematode pathogens. From the resulting 19,426 orthologous groups, 285 that consisted of one-to-one orthologs in all 24 species were used to reconstruct phylogenies. The resulting phylogenomic tree (Fig. 3A) is generally consistent with previously published standard multigene or whole genome phylogenetic analyses of Hypocreales and other model ascomycetes^24^,^26^. The analyses assign *D. coniospora* to Ophiocordycipitaceae, and reveal that this lineage shared an entomopathogenic ancestor. The topology of the phylogenetic tree shows that the three main nematophagous lifestyles (endoparasitism as in *D. coniospora* and *H. minnesotensis,* female and egg parasitism as in *P. chlamydosporia,* and nematode trapping as with *A. oligospora* and *Mo. haptotylum*) are polyphyletic in origin. The ability to infect nematodes and utilize them as a nutrient source likely have evolved independently and repeatedly in different fungal lineages.

To study the evoltuionary processes underlying lifestyle adaptation in more detail, we inferred a phylogenetic trees for each orthologous group and assigned evolutionary events (inventions, duplications and losses) to each node in these trees (Fig. S5, Table S7). This analysis shows that diversification and specialization during repeated lifestyle switches in this group involved mainly gene losses, with a more limited evolution of new gene inventions. Notably, losses are over-represented, while duplications and new inventions are strongly under-represented in the common ancestor of *D. coniospora* and *T. inflatum*, suggesting that genome simplification, perhaps supported by active RIP, is the dominant dynamics in this lineage. This is supported by the relatively low repeat content of the *T. inflatum* genome (estimated at 1.2%)^24^. In contrast, the lineage leading to *H. minnesotensis*, as well as that facultative endoparasite itself, has experienced an increase in the number of gene duplications and much fewer gene losses (Fig. S5), perhaps reflecting the retention of the saprophytic trophic mode.

#### Comparative genomics of parasitic lifestyles

We have compared the predicted proteome of *D. coniospora* with three subsets of fungi representing selected parasitic life strategies, including plant pathogens such as *Nectria haematococca*, *F. oxysporum*, and *Claviceps purpurea*; entomopathogens such as *T. inflatum, M. robertsii,* and *B. bassiana*; and nematophagous fungi such as *A. oligospora*, *P. chlamydosporia*, and *H. minnesotensis*. The analysis supports the existence of a “core genome”, represented by a set of 5,836 orthologous groups shared by these selected fungi (Fig. 3B).

In spite of their very different hosts, the hypocrealean plant and insect parasites included in this analysis adopt infection cycles that are broadly comparable to that of *D. coniospora*. These similar infectious mechanisms may be reflected by the large number of orthologous groups that is shared by *D. coniospora* with these fungi (6,251, Fig. 3B). On the other hand, the large number (4,162) of orthologous groups that are present in at least two life-strategy groups but absent in *D. coniospora*, are likely to represent functionalities that were lost due to degradation of the saprophytic lifestyle in *D. coniospora*. Outside the “core genome”, *D. coniospora* shares more orthologous groups with the model insect pathogens (336 groups) than with the plant pathogens (157 groups, Fig. 3B), in agreement with the higher similarity of these hosts. Strikingly, the number of orthologous groups shared with the entomopathogens outside the “core genome” is also higher than the one that is common with the representative nematophagous fungi (entomopathogens: 336 *vs*. nematophagous fungi: 297, Fig. 3B). This suggests that *D. coniospora* inherited its infection apparatus from its entomopathogenic ancestors and tuned these mechanisms to adapt to its new host. Nevertheless, the common genes present in these four nematode parasites, and especially those that are missing from the selected representatives of the other life-strategy groups, may provide a list of host-specific genes for nematode recognition, adhesion and/or digestion. Surprisingly, there are only a very limited number of genes that are present exclusively in the nematophagous fungi but not in the other three groups (56 *D. coniospora* proteins in 54 orthologous groups, Table S7D). 33 of these proteins do not have an identifiable Pfam domain. Most of the rest are predicted to be hydrolytic enzymes, transferases, transporters or regulatory proteins that may be involved in the digestion and uptake of host materials (Table S7D). In addition, *D. coniospora* also features 143 proteins in 83 groups that do not have orthologs in any of the other 23 fungal species analyzed. Most of these proteins (70.6%) do not have identifiable Pfam domains (Table S7E). Within those with an identifiable Pfam domain, surface or surface binding proteins comprise the largest portion (6 domains, 13 proteins), followed by transcriptional factors or regulators (3 domains, 4 proteins), hydrolytic enzymes (2 domains, 3 proteins) and toxins (2 domains, 9 proteins). While most of the *D. coniospora*-specific proteins were found to be expressed at low levels, three proteins were substantially upregulated during nematode infection, including a surface protein (DCS_00041, GLEYA-domain containing protein), a protein without a Pfam domain (DCS_05566) and a protein with a glycine-rich domain (DCS_06014) (Table S7E).

To further dissect genomic adaptations to nematophagy, we compared the predicted proteomes of *D. coniospora* (a near-obligate endoparasite that is also able to infect nematode eggs in experimental settings)^32^, *H. minnesotensis* (a facultative endoparasite of nematode juveniles), *P. chlamydosporia* (a female and egg parasite that may also infect L2 juveniles)^33^, and *A. oligospora* (a nematode trapping fungus that can degrade plant material and may also colonize roots). The nematode trapping fungus features a very large number (3,357) of orthologous groups without representatives in the other three nematode parasites, and a small number (406) orthologous groups aside from the “core genome” that are shared with *D. coniospora* (Fig. 3C). These numbers reflect the larger phylogenetic distance of *A. oligospora* from the hypocrealean nematode parasites, but also its specialized adaptations to capture its host^19^. The female and egg parasite *P. chlamydosporia* and the facultative endoparasite *H. minnesotensis* harbor 1,789 and 1,184 orthologous protein groups without representative in the other nematode parasites. They share similar numbers of orthologous protein groups outside the “core genome” with *D. coniospora* (2,287 and 2,412 groups, respectively, for *P. chlamydosporia* and *H. minnesotensis*), in spite of the much closer taxonomic position of *H. minnesotensis* with *D. coniospora* and their shared endoparasitic life strategy. Finally, *D. coniospora* features a very limited number (255) protein orthogroups not present in the three other nematode parasites (Fig. 3C). Taken together, these findings indicate that these representatives of the three main nematophagous parasitic strategies developed their own sets of host-specific genes is in spite of the common host, in accordance with the proposed polyphyletic origin of nematophagy in Ascomycetes.

#### Adaptation to a reduced lifestyle repertoire leads to proteome contraction

**Figure 4:**
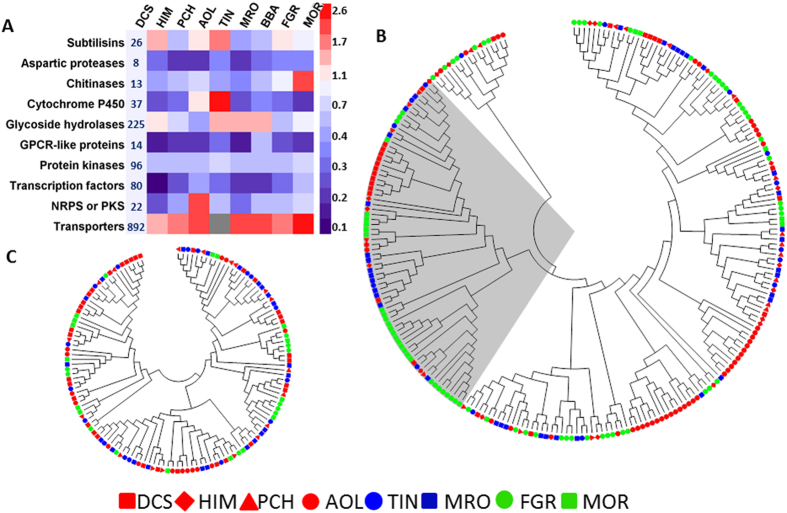
Protein family contractions and expansions in *D. coniospora*. (**A**) For each protein family, the number of family members encoded in the genome of *D. coniospora* was divided by the number of family members encoded in the comparator fungus. The heat map shows the resulting ratios. The DCS column shows the numbers of proteins in the indicated families encoded in the *D. coniospora* genome. Grey color: no data available. (**B**) Homologous clustering of subtilisin-like serine proteases and (**C**) chitinases from eight fungi pathogenic to different hosts. The shaded area marks the pr1C subtilisin-like serine protease subfamily. AOL: *A. oligospora* (nematode trapping fungus); BBA: *B. bassiana* (entomopathogen); DCS: *D. coniospora* (near-obligate nematode endoparasite); FGR: *F. graminearum* (phytopathogen); HIM: *Hirsutella minnesotensis* (facultative nematode endoparasite); MRO: *M. robertsii* (entomopathogen); MOR: *Ma. oryzae* (phytopathogen); PCH: *P. chlamydosporia* (nematode female and egg parasite); TIN: *T. inflatum* (entomopathogen).

Adaptation to an obligate nematode endoparasitic lifestyle sets *D. coniospora* on an evolutionary trajectory where genes for the utilization of a wide variety of plant-based nutrients, and genes for survival in a wide range of environmental conditions (including those encountered as a free living saprophyte or as a phytopathogen) may no longer be advantageous. The resulting loss of these genes further restricts lifestyle choices for the fungus and increases its reliance on the nematode prey for survival, culminating in an essentially obligate endoparasitic lifestyle restricted to nematode hosts. The complete genome of *D. coniospora* is predicted to encode 8,281 proteins, with 3,965 mapped to the KOG eukaryotic orthologous groups of proteins and 1,672 assigned to KEGG metabolic pathways (Fig. S6, Table S8). These numbers are significantly smaller than what has been reported for most other Ascomycetes that are facultative saprophytes (Table S8, S9). Various families of proteases, hydrolases and cytochrome P450s involved in carbohydrate, lipid and amino acid metabolism, and plant biomass degradation are especially depleted in *D. coniospora* as compared to facultative parasitic fungi with diverse life-strategies (Figs 4A and S7, Tables S10 and S11, Supplementary Results). In addition, the contractions of protein families involved in secondary metabolism, xenobiotic degradation and signal transduction (the transcription factors, protein kinases, and GPCR-like proteins) may result from the reduced exposure of the fungus to various environments outside the body of its prey (Fig. 4A, Table S7). In a stark contrast to the contraction of many protein families, the number of transporters encoded in the *D. coniospora* genome is remarkably high (Fig. 4A, Supplementary Results), which may reflect the heightened dependence of *D. coniospora* on host nutrients, and the acute need of this pathogen to protect itself from host-derived defense substances.

In spite of the overall simplification of the proteome, 4,371 *D. coniospora* proteins (50.4%) still lack significant matches to gene functional annotation databases (Pfam, KEGG, and GO, E value < 2.5e^−5^). This is a much larger portion than that found in typical ascomycete genomes (approximately 33%)^24^, and highly exceeds that in the facultative nematode endoparasite, *H. minnesotensis* (14.0%)^7^. Surprisingly, 1,288 predicted proteins even lack clear orthologs (E-value < 2.5e^−5^) in the NCBI non-redundant protein sequences (nr) database. In comparison, *M. acridum* and *M. robertsii* feature 434 and 615 unique proteins, respectively^26^. The abundance of unique or not easily annotatable proteins in the background of a reduced predicted proteome suggests an interesting evolutionary dynamics where genome contraction and the invention of novel or fast-evolving proteins may occur simultaneously during the adaptation of the fungus to its specialized lifestyle.

### Adaptations to a nematode endoparasitic lifestyle

#### Stress and pheromone sensing and signaling

**Figure 5:**
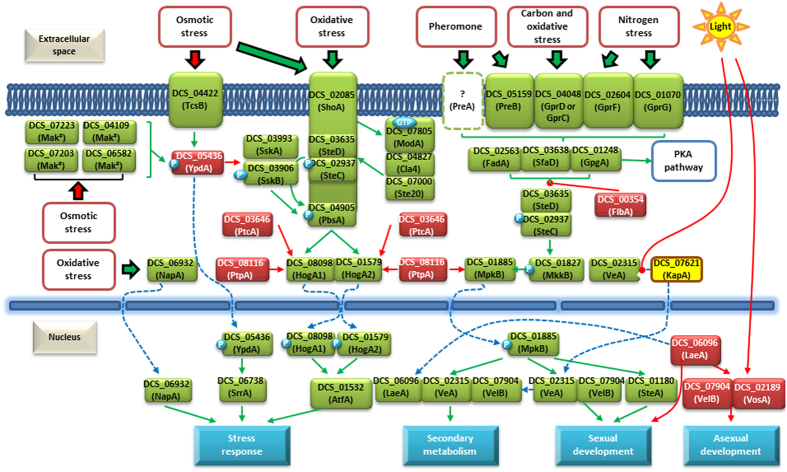
Stress and pheromone sensing and signaling pathways in *D. coniospora*. The pathways were reconstructed using an *As. nidulans* model^34^,^81^. ^#^orthologs of the Mak1-3 sensor histidine kinases present in *Schizosaccharomyces pombe*. Green arrow: activation; red arrow: repression; blue dashed arrow: formation of protein complexes or transport through membranes; yellow box: facilitation of membrane transport.

Similarity searches against a collection of verified stress and pheromone response elements^34^ allowed the reconstruction of these signaling pathways in *D. coniospora* (Fig. 5, Table S13, Supplementary Results), revealing important differences with the archetypical pathways described in *Aspergillus nidulans*^34^,^35^,^36^,^37^,^38^. Thus, the *D. coniospora* genome encodes four orthologs of the *S. pombe* Mak1/2/3-type oxidative stress sensor kinases^39^: this may reflect the importance of responding to free radical attacks launched by the immune system of the host. Infection by *D. coniospora* elicits a rapid innate immune response in *C. elegans* through multiple MAPK cascades and STAT-like transcription factors^40^,^41^,^42^,^43^. It is possible that the fungal-derived MAPK paralogs also interfere with the signaling pathways of the host: infection with *D. coniospora* led to the down-regulation of several M13 peptidase classes that act on small signaling peptides, and to the enrichment of smaller peptides and proteins relative to those induced by bacterial infections^43^. Further experiments should shed light on the functional partition and/or neofunctionalization of the *D. coniospora* MAPKs and their potential effects on nematode innate immunity and signaling pathways.

Remarkably, this fungus also encodes two HogA-type MAPKs that may be involved in the response to high osmolarity^44^, although a HogA paralog in *As. nidulans* was seen to be dispensable in osmoadaptation^45^. Surprisingly, *D. coniospora* features only one of the two G-protein-coupled pheromone receptors that are necessary for normal levels of ascospore and cleistotechia formation in *As. nidulans* (Fig. 5). Elimination of the genes for both of these receptors results in the complete absence of self-fertilization in that fungus^46^. Considering the genomic evidence for the existence of a cryptic, heterothallic sexual state in *D. coniospora* (see above) and the retention of a PreB (GprA) ortholog, the significance of the absence of a PreA ortholog remains obscure.

#### Small secreted proteins (SSPs), pathogen-host interaction (PHI) proteins, and pathogenicity islands

SSPs are candidate effectors that may manipulate the host. The *D. coniospora* genome encodes 312 SSPs that cluster into 257 families, relatively few compared to the facultative nematode endoparasite *H. minnesotensis* (494 SSPs) and the nematode-trapping fungus *Mo. haptotylum* (695 SSPs). To escape recognition by the host and the elicitation of host-defense responses, effectors evolve at a fast pace^47^. Thus, half of the *D. coniospora* SSP families (124 of 257) are species-specific: at least some of these may contribute to the unique strategies of *D. coniospora* to infect its host and to suppress host immunity. Additional 45 *D. coniospora* SSP families are classified as ‘sparse’, and show a scattered distribution on the species tree with more ‘sparse’ SSP families shared amongst nematophagous and entomopathogenic fungi (Fig. S8). The majority of the *D. coniopora* SSPs do not have clear orthologs with known functions, and only 28% contain identifiable functional domains (Table S14). The most frequently detected Pfam domains in SSPs were associated with surface proteins, toxins, protective proteins and hydrolytic enzymes (Table S14). These may be involved in mediating contact or communication between the fungus and its environment^48^; attacking the host^49^; protecting against oxidative stress^50^; or digesting the nematode cuticle. Most predicted SSPs are selectively transcribed during one or more growth stages (Supplementary Results). Remarkably, 210 SSPs were expressed during growth with the nematode prey. 22 of these were upregulated by >2-fold compared to other life stages, with the majority encoding predicted proteins or hydrolytic enzymes. These results suggest a dynamic interaction between SSPs and the host defense system. Interestingly, the immune response system of *C. elegans* also features rapidly evolving genes encoding small proteins that may be part of poorly understood regulatory pathways governing small peptide signaling^43^, thus indicating a potential “small arms” race between host and pathogen.

14.3% of the *D. coniospora* genome encodes orthologs of the genes in the pathogen-host interaction (PHI) database^51^, a proportion comparable to that of other pathogenic fungi closely related to *D. coniospora*, such as *M. robertsii* and *M. acridum*^26^ and much higher than that of the nematode endoparasite, *H. minnesotensis* (9.2%)^7^. 1,129 of these 1,768 genes correspond to PHI entries whose products are known virulence factors or affect cell viability (Fig. S9). There are ~300 pathogenicity clusters containing up to 12 consecutive PHI genes and/or small secreted proteins, scattered along the three chromosomes outside of the centromeres and the rDNA region (Fig. 2). None of these clusters exceed 100 kb each, although 14 clusters are larger than 50 kb. Some clusters are co-regulated (*e.g.*, genes DCS_05926 to DCS_05934 or DCS_07355 to DCS_07363), but the genes in the majority of these clusters do not show a common expression profile. Taken together, the evolution of *D. coniospora* may have involved the gradual accumulation of pathogenicity-related genes, instead of the acquisition of large pathogenicity islands as seen in *Fusarium* spp.^52^.

#### Surface proteins

Adhesion of *D. coniospora* conidia to specific regions of the nematode body has been suggested to be mediated by surface proteins recognizing carbohydrates or peptides on the nematode cuticle^14^,^15^. Thus, putative surface proteins such as lectins, agglutinin-like surface (ALS) proteins, hydrophobins, adhesins, and CFEM-domain or GLEYA-domain containing proteins (Table S15) encoded in the *D. coniospora* genome are candidates for host surface recognition and adhesion. The contribution of lectins to host surface adhesion in nematophagous fungi is controversial^14^, although addition of various lectins impaired the attachment of *H. minnesotensis* to *C. elegans*^7^. Compared to the nematode-trapping fungi (69 in average)^7^, the lectin family is contracted in *H. minnesotensis* (40 representatives), and further drastically simplified in *D. coniospora* (only three lectin-coding genes and three additional enzymes also containing lectin domains). In contrast to *H. minesotensis*^7^, the *D. coniospora* lectins all displayed low expression levels during the conidia and infection stages (Table S15C), suggesting that lectins may not serve as major mediators for nematode host attachment for the obligate endoparasite. The *D. coniospora* genome shows an expansion of the large cell-surface glycoproteins of the ALS-domain protein family, and the carbohydrate-binding GLEYA-domain surface protein family^53^. While ALS-domain proteins are implicated in adhesion to host surfaces in *Candida albicans*^54^, none of the 10 ALS-encoding genes were transcribed at a high level in *D. coniospora* (Table S15B). However, one (DCS_00041) of the nine GLEYA-domain proteins was both upregulated and expressed at a high level during conidiogenesis and nematode infection. A protein with a GLEYA domain was also over-represented in the proteome of the nematode-trapping knobs of *Mo. haptotylum*^53^. Promisingly, high-level transcription of the *D. coniospora* adhesin DCS_00989 was also associated with conidiogenesis and infection (Table S15B). While this protein lacks orthologs in other nematophagous fungi, it is orthologous to Mad1 of *M. robertsii* that is involved in the specific adhesion of the entomopathogen to the cuticle of the insect prey^55^. Amongst the three hydrophobins encoded in the *D. coniospora* genome (Table S15B), DCS_04895 is specific to the nematophagous fungi in Fig. 3B and is highly expressed in mycelia. Another hydrophobin (DCS_08052) is selectively expressed during conidiogenesis. Taken together, the distribution of surface proteins amongst entomopathogenic and nematophagous fungi, and their expression patterns suggest that instead of a single surface protein, a combination of these proteins determines the adhesion mechanisms as well as host ranges of these fungi.

#### Hydrolytic enzymes

Subtilases, metalloproteases and acid phosphatases have been implicated in *D. coniospora* in the softening of the nematode cuticle that precedes penetration by appressoria^11^,^16^. In general, hydrolytic enzymes are under-represented in the *D. coniospora* genome, perhaps as a consequence of the loss of the saprophytic and phytopathogenic trophic modes (Fig. 4A). Nevertheless, subtilisin-like serine proteases (subtilases) and metalloproteases are well represented and even expanded in *D. coniospora*, as opposed to *H. minnesotensis* (Fig. 4A, Table S7B). Subtilases are effective virulence and pathogenicity factors for different hosts^56^,^57^,^58^, disrupting the integrity of the cuticle of nematode and insect hosts alike, and are also important for the penetration of plant surface barriers^18^,^19^,^26^. Correspondingly, subtilases from eight pathogenic fungi did not show a selective clustering according to the host (Fig. 4B). Most *D. coniospora* subtilases fall into the pr1C subfamily also prevalent in the phytopathogen *F. graminearum* and the entomopathogen *M. robertsii* (Fig. 4B). Two pr1C subfamily enzymes (DCS_07079 and DCS_05830) were upregulated in *D. coniospora* conidia, and another two were specifically induced (albeit at moderate levels) in the presence of the nematode (DCS_06133 and DCS_01914, Table S15B). Another two pr1C and one pr1A subtilase (DCS_05134, DCS_03317 and DCS_01961, respectively) were preferentially transcribed during the mycelial stage. DCS_01961 is orthologous to SPM1, a validated pathogenicity factor of *Ma. oryzae*, and to VCP1 of *P. chlamydosporia* that removes the outer proteinaceous vitelline layer of the nematode egg^18^,^59^. It is also orthologous to a subtilisin in *H. minnesotensis* (HIM_09336) that is upregulated during the nematode penetration stage^7^. The domain architectures of the *D. coniospora* subtilases are highly conserved, but their *N*- and *C*-termini are variable, suggesting that these proteases are partitioned to different compartments. Together with their life stage-dependent expression, this suggests that some subtilases may be involved in host penetration, while others may function when the developing conidiophores erupt from the host. For nematophagous fungi such as *D. coniospora*, breaching the cuticle wall through cutinases and proteases is essential for infection while other proteases may be targeted to specific organs of the host for nutrient supply^15^,^58^.

Other *D. coniospora* enzyme families potentially involved in cuticle degradation (such as chitinases, acid phosphatases and metalloproteases) or those taking part in the decomposition, detoxification and biosynthesis of various compounds^26^ (such as dehydrogenases, monooxygenases, and cytochrome P450s) all show overall contraction with selective expansion of certain subfamilies that presumably support the endoparasitic lifestyle of the fungus (Fig. 4A,C, Table S15, Supplementary Results).

#### Secondary metabolism

Secondary metabolites produced by *D. coniospora* may function as virulence factors killing the nematode; stress response elements mitigating host defenses; and antibiotics defending the nematode carcass from other microorganisms. *D. coniospora* harbors 17 secondary metabolite biosynthetic gene clusters organized around genes encoding 11 nonribosomal peptide synthetases (NRPS), 8 polyketide synthases (PKS) and 3 NRPS-PKS hybrids (Supplementary Results, Fig. S10, Table S16). These modest numbers suggest that the secondary metabolome of *D. coniospora* underwent substantial contraction upon the degradation of the saprophytic and phytopathogenic trophic modes (Fig. 4A). This is especially striking when compared to the facultative endoparasite *H. minnesotensis* where the number of secondary metabolic gene clusters underwent a very noteworthy expansion (94 clusters)^7^. In particular, the *D. coniospora* genome does not encode a canonical PKS-NRPS^60^,^61^ (Supplementary Results), and features fewer PKSs compared to closely related fungi, e.g., *P. chlamydosporia* and *H. minnesotensis* (15 PKSs each)^7^,^18^, *B. bassiana* (13 PKSs)^62^, and *M. robertsii* (24 PKSs)^62^. Remarkably, *D. coniospora* even lacks orthologs to PKSs producing melanin and other spore pigments in Ascomycetes. This simplification was unexpected as melanin pigments are common virulence factors that help mitigate ROS from the host^63^ (see below).

Nonribosomal peptide siderophores are important for iron homeostasis, but also frequently serve as virulence determinants^64^. *D. coniospora* features a conserved coprogen-type siderophore cluster whose transcription was elevated during the mycelial growth phase. Extracellular coprogens are central to siderophore-assisted iron acquisition (SAIA)^65^,^66^. *D. coniospora* also features a biosynthetic gene cluster for a ferricrocin-type siderophore that may be important for the intracellular storage and sequestration of excess iron to avoid ROS formation. The coprogen and ferricrocin NRPS genes (DcNRPS6 and DcNRPS9, respectively) show identical module and domain compositions (Fig. S10) to the appropriate siderophore synthetases of fungi^60^. Further iron acquisition mechanisms by reductive iron assimilation and heme (but not heme-protein) utilization are discussed in the Supplementary Results.

We predict that a cluster encoding DcPKSs 3, 4 and 5 may be responsible for the production of an as-yet unidentified benzenediol lactone^67^,^68^. Benzenediol lactones display a wide range of biological activities including immune system modulatory effects^67^,^68^. In the absence of melanin, this benzenediol lactone, the siderophores, and perhaps other unidentified factors regulated by the Mak paralog sensor kinases discussed in a previous section may together participate in a network to sense, respond to and mitigate attacks by the host immune system on the fungus.

**Figure 6:**
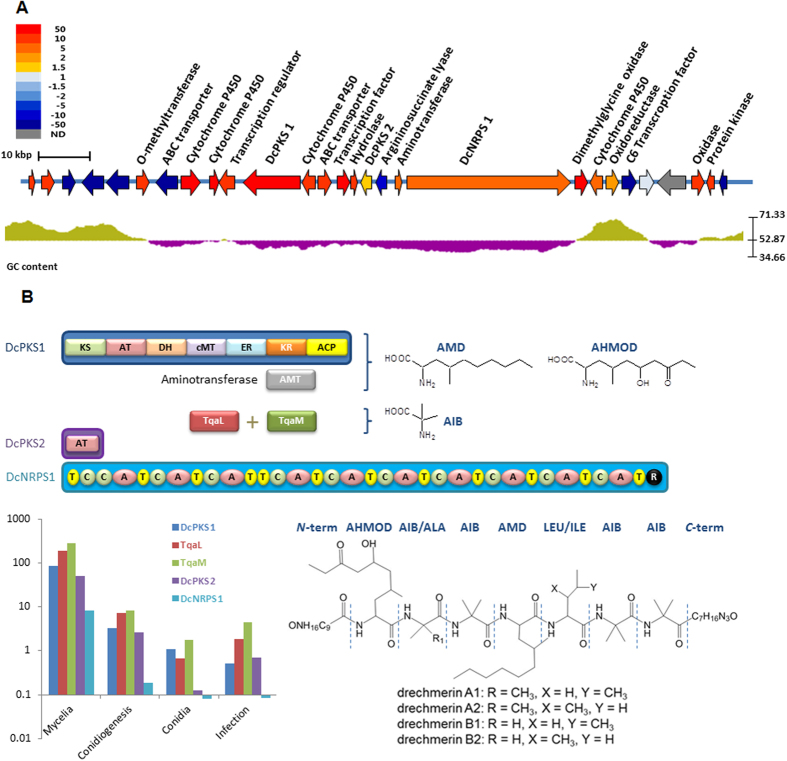
The *D. coniospora* peptaibiotic gene cluster and proposed biosynthetic pathway of drechmerins. (**A**) Genes in the genomic locus are color-coded to show their relative changes in expression in the mycelial growth stage as compared to conidia (measured by qRT-PCR and calculated by the 2^−ΔΔC^_T_ method^82^). This result corroborates NGS transcriptomic sequencing results (Table S18B). GC content is shown for a sliding window of 40 bp. (**B**) Proposed model for the assembly of drechmerin A and transcript abundance (in FPKM) of key genes during the different growth phases. The proposed structures of the most abundant drechmerin congeners are also shown (see also Table S19 and Fig. S14). Analytical data are insufficient to elucidate the structures of the *N*- and *C*- termini, however, accurate mass determination of molecular formulae for the full peptides constrain these blocking groups to the formulae shown. MS data in hand cannot constrain the residue occupying the position *C*-terminal to the AMD unit to either LEU or ILE. The drechmerin A and B fractions could consist of either or both of these possible isomers. Domain abbreviations: KS: ketoacyl synthase; AT: acyltransferase; DH: dehydratase; cMT: *C-*methyltransferase; ER: enoyl reductase; KR: ketoacyl reductase; ACP: acyl carrier protein; C: condensation; A: adenylation; T: thiolation; R: reductive release domain. Structure abbreviations: AIB: α-aminoisobutyric acid; LEU: leucine; ILE: isoleucine; AHMOD: (2*S*,4*S*)-2-amino-6-hydroxy-4-methyl-8-oxodecanoic acid; AMD: 2-amino-4-methyldecanoic acid.

Phylogenetic analyses indicated that DcNRPS1 may be involved in the biosynthesis of a peptaibiotic-type nonribosomal peptide (Fig. S11). Genes clustered with DcNRPS1 also encode further enzymes for the biosynthesis, transport and transcriptional regulation of this predicted peptaibiotic. The DcNRPS1 cluster shows a lower GC content compared to flanking genomic regions (Fig. 6A). While these flanking sequences exhibit synteni with the *M. acridum* and *M. robertsii* genomes, the DcNRPS1 cluster itself is missing from those entomopathogens. The DcNRPS1 locus and its flanking sequences also lack synteny with the *H. minnesotensis* genome, and DcNRPS1 itself has no ortholog in the facultative endoparasite^7^. These analyses suggest a heterologous origin for this cluster. *D. coniospora* fermentations yielded a series of >20 closely related linear non-ribosomal peptide analogues which we named “drechmerins”. The structures of the dominant drechmerin analogues were determined by mass spectrometry (MS) and tandem mass spectrometry (MS/MS) (Fig. 6B, Supplementary Results, Fig. S14), and shown to contain the non-canonical amino acid analogues AIB (α-aminoisobutyric acid), AHMOD ([2*S*,4*S*]-2-amino-6-hydroxy-4-methyl-8-oxodecanoic acid) and AMD (2-amino-4-methyldecanoic acid). The proposed biosynthetic pathway of drechmerins, including that of AHMOD and AMD on DcPKS1, are detailed in the Supplementary Results. Drechmerin-containing crude extracts showed antibacterial, antifungal and nematicidal activity (Supplementary Results). Considering that the drechmerin biosynthetic cluster is highly transcribed during mycelial growth while repressed in other growth stages, this peptaibiotic may serve as an aggressive virulence factor contributing to the killing of the nematode prey, and/or may defend the nematode carcass from invading fungi or bacteria (Fig. 6B). Destruxins and other toxins from closely related Hypocrealean entomopathogens have also been postulated to serve similar roles, providing yet another example of evolutionary convergence for a requisite biological need^62^,^69^.

## Conclusions

The *D. coniospora* genome complements our understanding of fungal nematophagy by revealing genomic adaptations to endoparasitism, the third major nematode parasitic strategy in fungi in addition to female and egg parasitism (*P. chlamydosporia*^18^) and nematode trapping (*A. oligospora*^19^). A high quality finished assembly revealed a 32.5 Mb genome organized in only three inferred chromosomes, at least one of which is a result of a chromosomal fusion. Global syntheny with the closely related insect pathogen *T. inflatum* was detected. Phylogenomic analyses assign *D. coniospora* to Ophiocordycipitaceae, and reveal that this lineage shared an entomopathogenic ancestor. The *D. coniospora* genome features a relatively high repeat content including regional centromeres, telomeres, an rDNA island, and a large number of species-specific repeat regions. The genome is molded by transposable elements and an active repeat-induced point mutation (RIP) genome defense system. While a teleomorph has not been identified for *D. coniospora*, genomic evidence suggests the existence of a cryptic sexual cycle with heterotallic mycelia.

Comparison with the facultative nematode endoparasite *H. minnesotensis*^7^ reveals that adaptation of *D. coniospora* to the near-obligate nematode endoparasitic trophic mode of this fungus involved both genome contraction and limited new gene inventions. The *D. coniospora* genome features only 8,281 protein-encoding genes as a result of significant reduction of many gene families, including cytochrome P450s, hydrolytic enzymes, and regulators. While central elements of stress signaling pathways are well conserved in *D. coniospora*, stress sensors and transcriptional regulators underwent category-specific simplifications. These losses led to the degeneration of the saprophytic fitness of this organism. On the other hand, orthologs of pathogen-host interaction proteins are easily identifiable in *D. coniospora*, and the genome also contains a large number of putative small secreted proteins, many of which appear species-specific. While hydrolytic enzyme families are generally contracted in *D. coniospora*, some subfamilies of these enzymes underwent expansions. These enzymes may be involved in host penetration, and together with a significantly expanded repertoire of transporters, may contribute to the utilization of the nematode prey as a nutrient source. *D. coniospora* acquires iron from its host by producing hexadentate siderophores, but may also utilize reductive iron assimilation. In addition, genomic evidence for the utilization of heme (but not heme-proteins) was also obtained. The secondary metabolome of the near-obligate endoparasite is also simplified, but still includes 17 gene clusters organized around PKS, NRPS and NRPS-PKS genes. One of these clusters is responsible for the biosynthesis of drechmerins, novel peptaibiotics that show antibiotic, antifungal and nematicidal activities. Transcriptomic analyses comparing the mycelial, condiogenesis, conidial, and nematode infection stages of *D. coniospora* provided support for a dynamically adapting transcriptome and highlighted genes preferentially expressed in these different life stages. Thus, our data emphasize the basic malleability of the Hypocrealean genome, with *D. coniospora* eliminating genes advantageous for saprophytic lifestyle, modulating and adapting elements that its cohort species utilize for entomopathogenesis, and expanding protein families necessary for its nematode endoparasitic lifestyle.

## Materials and Methods

### Fungal strain and growth conditions

An axenic culture of *Panagrellis redivivus* was infected with spores of *D. coniospora* ARSEF 2468 (equivalent to CBS 615.82). The strain used in these studies, *Drechmeria coniospora* ARSEF 6962 was obtained by re-isolating the fungus from a single axenically-grown *C. elegans* nematode that had been infected by a single spore. *D. coniospora* ARSEF 6962 was maintained on liver/kidney agar plates (100 g/L homogenized pork liver; 100 g/L homogenized pork kidney; 0.5% NaCl; 1.5% agar) at 25 °C for 8 days. Conidia were collected by flooding the plates with sterile water or 0.85% NaCl; pH 7.0. Peptaibiotics were produced in liquid SDYC medium (40 g/L dextrose, 10 g/L neopeptone, 10 g/L yeast extract, 10 g/L casamino acids) at 25 °C with shaking at 180 rpm for 4 weeks.

### Genome sequencing, assembly, and optical mapping

Two Illumina (insert size: 0.5 kb and 3 kb, respectively) and one Roche 454 paired-end (8 kb insert) whole genome shotgun libraries were constructed. Short reads generated from these libraries were assembled using Velvet, and the contigs were joined into scaffolds with Illumina meta-pair reads and 454 pair-end reads. PacBio continuous long reads (CLR) were used for scaffolding and filling gaps with PBjelly. The finished chromosome-length pseudomolecules were constructed by anchoring and orienting the final sequence scaffolds onto the whole genome physical map of *D. coniospora* generated by optical mapping. Optical maps were prepared using Argus (OpGen) according to the methods described previously^70^. Briefly, high molecular weight DNA was prepared by embedding *D. coniospora* protoplasts in low melting temperature agarose plugs, followed by treatment with lysing solutions. The genomic DNA was recovered after thoroughly rinsing the plugs in TE followed by melting the plugs at 42 °C and subsequent treatment with β-agarase. The high molecular weight DNA was then immobilized as individual molecules on Optical Chips, digested with the restriction enzyme *Bgl*II (New England Biolabs), fluorescently stained with a staining kit (OpGen) and positioned onto an automated fluorescent microscope system for image capture and fragment size measurement. The resulting collections of high resolution single-molecule restriction maps were assembled to produce whole genome ordered restriction maps.

### Gene prediction and annotation

Coding genes were predicted using an RNA-guided annotation strategy with a modified PASA pipeline and compensated using transcriptomic data. Preliminary models generated with AUGUSTUS, GlimmerHMM, GeneMark and SNAP were merged by EVM (Evidence Modeler). All predicted gene models were functionally annotated based on their sequence similarities to genes and proteins in the NCBI nucleotide (nt), non-redundant (nr), UniProt/Swiss-Prot protein databases, and the conserved protein domain database InterProScan. All genes were classified using the Gene Ontology (GO), eukaryotic orthologous groups (KOG) and KEGG metabolic pathways. Repeat sequences were masked throughout the genome using Repeat Masker (version 3.2.9) and the RepBase library (version 16.08). Selected gene family members were identified using the following criteria: coverage and identity both above 50%, and E score <10-5. Small secreted proteins (SSPs) in *D. coniospora* were identified by surveying the genome for proteins that span 50–300 amino acids, contain a signal peptide (SignalP), and are not targeted to the membrane (TMHMM). Pathogenicity islands were detected by mapping at least three genes encoding predicted SSPs and/or orthologs of proteins in the PHIbase to within 15 kb windows on the chromosomes. The genome sequence assembly and the predicted gene and protein sequences were submitted to NCBI database, and appear under the accession number LAYC00000000 with locus tags in the format of DCS_00000.

### Transcriptome sequencing and analysis

Three biological replicates of *D. coniospora* in mycelia (day 4), early conidiogenesis (day 6), and conidia (day 8) life stages were collected from *in vitro* growth of *D. coniospora* on liver/kidney medium supporting morphogenesis and development. Infection samples were collected over the course of 8 days post-infection by growing *D. coniospora* on the live nematode, *C. elegans*. These four periods have been monitored by optical microscopy. Total RNA of these samples were extracted using the Trizol extraction method (Invitrogen). Poly-A mRNA was isolated with oligo-dT-coupled beads from 40 μg total RNA of each sample. After shearing, first strand cDNA was synthesized using random hexamers as primers and the Superscript II reverse transcriptase. After end repair and addition of a 3′-dA overhang, the cDNA was ligated with the Illumina paired-end adapter oligonucleotide mixture, and size-selected by gel purification to obtain 200 bp fragments. After 16 PCR cycles the libraries were sequenced using Illumina GAIIx and the paired-end sequencing module. Tophat was used to map mRNA reads to the *D. coniospora* genome assembly, and Cufflinks were used to calculate the expected fragments per kilobase of transcript per million mapped reads (FPKM) as a measure of expression levels for each transcript. Differential expression is defined as larger than two-fold (unless otherwise stated) change in expression between two of the three *in vitro* growth stages and a p-value < 0.05 (Fisher’s exact test).

Quantitative real-time PCR (qRT-PCR) was performed using a fourth biological replicate from each of the growth stages. This replicate was different from those used for transcriptome sequencing, and was used to confirm the transcription profiles of 50 genes over the four growth phases, including the genes predicted to have important functions and mentioned in the main text. cDNA samples were amplified with the SYBR Green PCR Master Mix (TaKaRa BIO Inc.) using an ABI PRISM 9700 Real Time PCR System (Applied Biosystems). The comparative CT (threshold cycle) method was used to determine the average fold change of mRNA by comparing the CT of the target gene to that of the reference gene (18S rDNA), as described previously^71^.

### Orthology and phylogenetic analysis

To define orthologous groups for the 24 selected species, we first constructed a network connecting putative homologs in which edges are weighted according to relative sequence similarity. This network was then clustered into broad families. For each family, we inferred a gene tree and predicted duplication events. We split into distinct orthologous groups those families for which a duplication event was inferred to occur in the last common ancestor of our set of 24 species (see Supplemental Materials and Methods for more detail). Of the initial 19,426 families, 285 consisted of 1:1 orthologs, and these were used to infer a species tree. First, a multiple sequence alignment (MSA) was constructed for each family using Clustal Omega^72^. These were trimmed using trimAl (-strictplus)^73^, the trimmed alignments were concatenated into a single alignment using a custom python script, and a tree was inferred using RAxML with 100 bootstrap replicates^74^. The set of gene trees was also used to study evolutionary dynamics in different parts of the species tree. To avoid inference of a large number of evolutionary events due to possible mistakes in the gene tree, we first calculated branch support for all gene trees inferred previously with the Shimodaira-Hasegawa test (RAxML -m PROTGAMMAIWAG -f J)^75^,^76^ and then used NOTUNG 2.8 (–threshold 80 –rearrange) to rearrange branches with support <80 such that the number of evolutionary events is minimized^5^. We used strict tree reconciliation with the species tree inferred based on 285 families that consists of 1:1 orthologs as implemented in ete2 to infer duplications and losses. Finally, the relative frequencies of three types of evolutionary events (invention, duplication, loss) were plotted on the branches of the species tree.

Phylogenetic analysis of NRPSs, hybrid NRPS-PKSs and AMP-binding enzymes was based on their adenylation (A) domains. MAFFT v 7.037b was used to align 559 A domains extracted from 295 proteins belonging to 33 taxa, mostly Ascomycota. The dataset included 70 enzymes whose secondary metabolite end products are known. A domains were identified using the PF00501 Hidden Markov Model from PFAM (http://pfam.sanger.ac.uk/). Cluster analysis was performed with FastTree 2.1.7 and the JTT amino acid substitution model. Branch support was calculated with the Shimodaira-Hasegawa test.

### Peptaibiotic isolation, structural analysis and bioactivity evaluations

*D. coniospora* fermentation broths were extracted against half volumes of dichloromethane, the extracts were dried *in vacuo*, and reconstituted in methanol. The extracts were fractionated using repetitive preparative HPLC, with mixtures of acetonitrile and H_2_O modified with TFA (0.1%) at a flow rate of 10 mL/min on a Shimadzu Shim-pack PREP ODS(H) Kit column (250 mm × 20 mm id., 5 μm particle size). Low resolution ESI-MS spectra were acquired on a Waters-Micromass ZQ-2000 spectrometer operated in positive ion mode using capillary and cone voltages of 3.6 kV and 40–150 V, respectively. HRESIMS and MS-MS spectra were acquired on a Waters XEVO G2 instrument employing collision energy of 50V. For accurate mass measurements the instrument was calibrated using NaI as a calibrant through the range m/z 150–1900. Final accurate mass estimates were made by combining 28–30 continuum scans, centering using the automatic peak detection algorithm provided by MassLynx 4.1 and applying lock-mass correction using leucine enkephalin (556.2771 Da for [M + H]^+^) as the internal lock standard. Additional ESI-MS/MS data were acquired on an ABI-SCIEX Q-trap-2000 spectrometer operated in positive ion mode using enhanced product ion scans at 1000 amu/sec through the range of m/z 50-1300. Declustering potential and collision energy were set at 160 V and 70 V, respectively. For all MS analyses samples were dissolved in MeOH and directly infused into the source by syringe-pump at a flow rate of 5μL/min.

Methylene-chloride extracts of the *D. coniospora* fermentation broths, as well as purified fractions, were used to determine antibacterial, antifungal, and nematicidal activities. Bacterial strains were maintained on nutrient agar, while fungal strains were cultivated on potato dextrose agar. Disk diffusion assays were performed as described^77^.

*Pratylenchus penetrans* axenic cultures were maintained on alfalfa sprout cultures and harvested using filtration through filter paper suspended over a wire mesh plate in sterile water overnight, and concentrated by centrifugation at 100 × g^78^. *Globodera rostochiensis* juveniles were hatched from cysts by soaking them in potato root diffusate for 1 week, and harvested by centrifugation as above^79^. We used a bioassay system similar to that described by Sanchez de Viala *et al.*^80^ using 24-well tissue culture plates in which nematodes are plated at approximately 20–30 nematodes/well, using 6 replicates per treatment. Test substances were dissolved in acetone and diluted in deionized water to give a final acetone concentration of 2%. Plates were incubated in the dark at 25 °C, and mortality was scored at 24, 48, and 72 h using an inverted microscope at 100 and 200× magnifications. Negative controls using water only and acetone at final formulation dosage showed mortality of 10% or less at 72 h. Assays were scored daily for non-motility and potential mortality, as evidenced by the darkening of the nematode interior, or by the lack of movement after use of a microprobe.

## Additional Information

**Accession codes:** This Whole Genome Shotgun project has been deposited at DDBJ/EMBL/GenBank under the accession LAYC00000000. The version described in this paper is version LAYC01000000.

**How to cite this article**: Zhang, L. *et al.* Insights into Adaptations to a Near-Obligate Nematode Endoparasitic Lifestyle from the Finished Genome of *Drechmeria coniospora. Sci. Rep.* **6**, 23122; doi: 10.1038/srep23122 (2016).

## Supplementary Material

Supplementary Information

Supplementary Tables

## Figures and Tables

**Figure 1:**
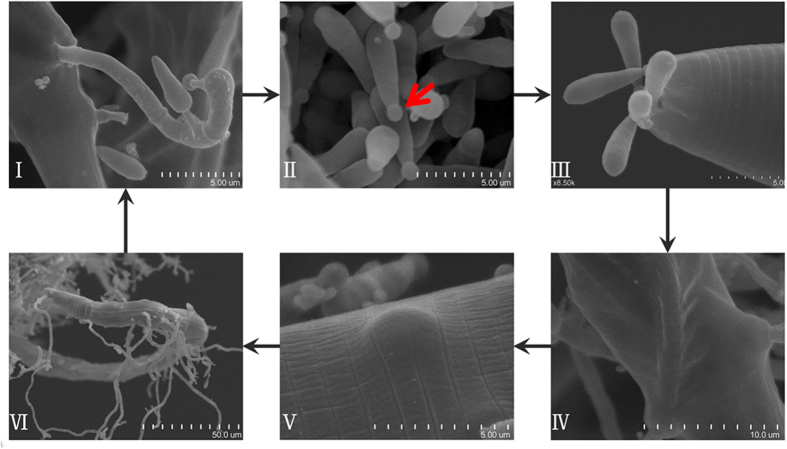
The infection cycle of *D. coniospora*. Scanning electron micrographs of *D. coniospora* infecting *C. elegans* are shown with scale bars. (**I**) Teardrop-shaped conidia form on individual pegs of the conidiophores on the external surface of the host. (**II**) Conidial maturation involves the development of one spherical adhesive knob (*red arrow*) at the distal end of each conidium, after release from the conidiiferous peg and separation from other spores (i.e. conidiogenesis and conidial maturation are spatially separated^13^). The conidia will remain dormant until attached to a new prey. (**III)** Conidia specifically adhere near the chemosensory organs on the head and the posterior region of the nematode^9^,^14^,^15^. (**IV**) Penetration of the nematode cuticle involves a combination of enzymatic action and mechanical force via appressoria, followed by vigorous growth of the trophic hyphae that invade the pseudocoel^6^,^12^,^15^. Invasion through the oesophagus or other natural openings of the nematode has not been observed^12^. **(V)** Death of the prey sets in after a short biotrophic phase. New conidiophores develop from bulbs at the tips of trophic hyphae inside the cadaver, tightly oppressed to the internal surface of the cuticle, preventing leakage of host nutrients. (**VI**) Conidiophores continue to develop while the whole nematode is expended by the fungus, yielding copious amounts (up to 5,000–10,000) of conidia from a single cadaver^12^.

**Table 1:** *D. coniospora* genome sequencing and assembly.

Sequencing Features	Value
Fold coverage	457.9×
N50 length of scaffolds (bp)*	4,137,305
N90 length of scaffolds (bp)*	1,535,228
Number of Ns in the assembly (per 10 kb)	190
Genome size (Mb)	32.5
Number of chromosomes	3
(G + C) percentage	55.0%
Exon (G + C) percentage	61.0%
Total length of coding sequences (Mb)	12.8
Repeat content	12.5%
tRNA genes	125
Nonrepetitive intergenic DNA	27%
Average gene size (kb)	2.3
Average number of exons per gene	3.8
Average number of introns per gene	2.0
Average intron length (bp)	42.4
Number of protein-encoding genes	8,281
Conserved hypothetical proteins	3,766 (47.1%)

^*^Measured before assembly into chromosomes.
