# Supplementary material for: Insights into Adaptations to a Near-Obligate Nematode Endoparasitic Lifestyle from the Finished Genome of Drechmeria coniospora
Source: Sci Rep. 2016 Mar 15;6:23122. doi: 10.1038/srep23122 (PMC4792172; doi:10.1038/srep23122)
Supplement: Supplementary Information [file srep23122-s1.pdf]

Supplementary Figures

Insights into adaptations to a near-obligate nematode endoparasitic lifestyle from the finished genome of *Drechmeria coniospora*

Liwen Zhang, Zhengfu Zhou, Qiannan Guo, Like Fokkens, Márton Miskei, István Pócsi, Wei Zhang, Ming Chen, Lei Wang, Yamin Sun, Bruno G. G. Donzelli, Donna M. Gibson, David R. Nelson, Jian-Guang Luo, Martijn Rep, Hang Liu, Shengnan Yang, Jing Wang, Stuart B. Krasnoff, Yuquan Xu*, István Molnár*, and Min Lin*

Contents

Figure S1. Long-range syntenic relationship between the *D. coniospora* and *T. inflatum* genomes 3

Figure S2. Distribution of species-specific repeats in the *D. coniospora* genome 4

Figure S3. Repeat-induced point mutations in two retrotransposon families 5

Figure S4. Nucleotide sequence alignment of 11 DDE transposases 6

Figure S5. Gene inventions, duplications and losses mapped to the phylogeny of selected fungi 7

Figure S6. KOG (EuKaryotic Orthologous Groups) functional classification of the predicted *D. coniospora* proteome[4] 8

Figure S7. Selected metabolic pathways that are depleted in *D. coniospora* as compared to five closely related facultative parasitic fungi 9

Figure S8. Analysis of the occurrence of *D. coniospora* SSP families in a set of reference fungal genomes 10

Figure S9. Predicted pathogen-host interaction (PHI) proteins in *D. coniospora* with verified participation in pathogenicity or cell viability 12

Figure S10. Domain structures of NRPS and PKS encoded in the genome of *D. coniospora* 13

Figure S11. Phylogenetic tree of the adenylation domains of the NRPSs and NRPS-PKSs of *D. coniospora* and other related fungi 14

Figure S12. Distribution of the number of cysteines in small proteins in *D. coniospora* 15

Figure S13. Homologous clustering of cytochrome P450s from *D. coniospora* (marked with red dots) and *F. oxysporum* (marked with green dots) 16

Figure S14. Mass spectrometric characterization of the structures of the main drechmerin congeners 22

Figure S15. Transcription levels of the NRPS, PKS and NRPS-PKS genes of *D. coniospora* during the four life stages 24

Figure S16. MultiGeneBlast alignments of selected secondary metabolite biosynthetic clusters in *D. coniospora* with syntenic clusters in other fungi 27

References 28

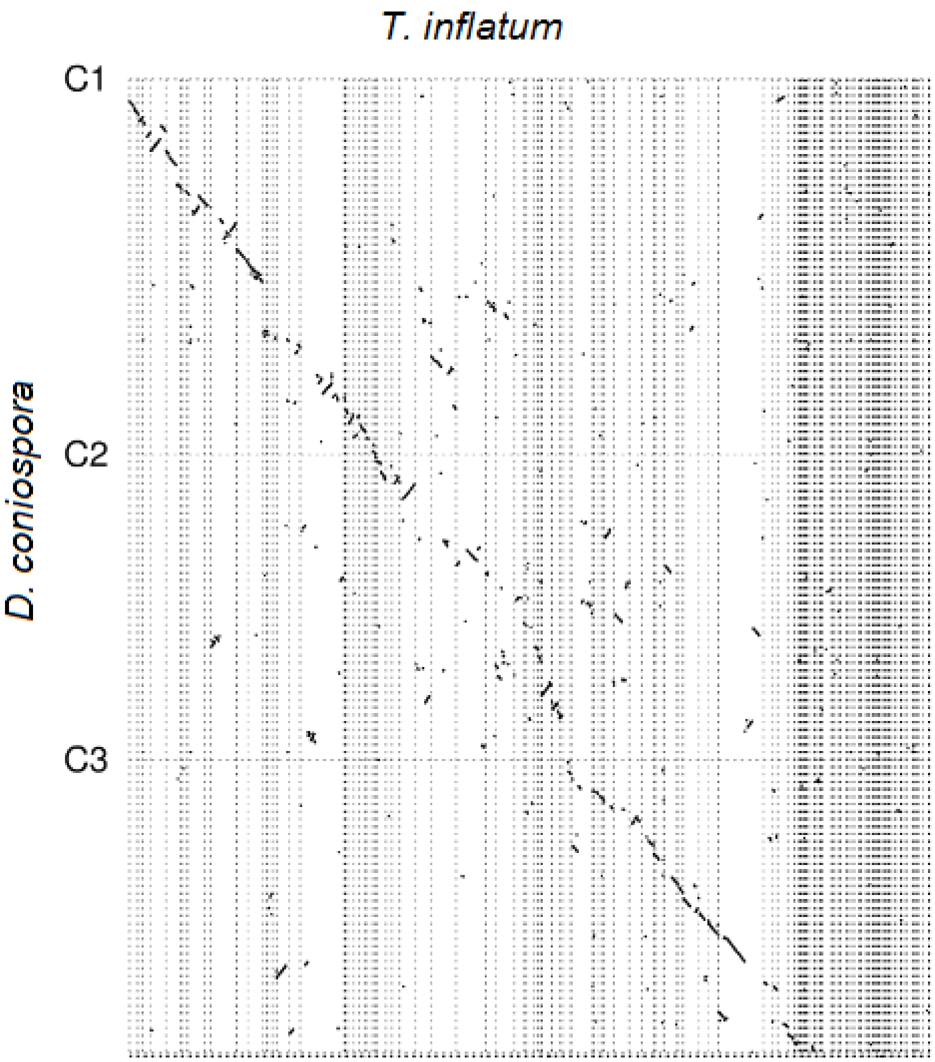

Figure S1. Long-range syntenic relationship between the *D. coniospora* and *T. inflatum* genomes

Y axis: The three chromosomes of *D. coniospora*; X axis: *T. inflatum* scaffolds oriented using the *D. coniospora* genome as a reference.

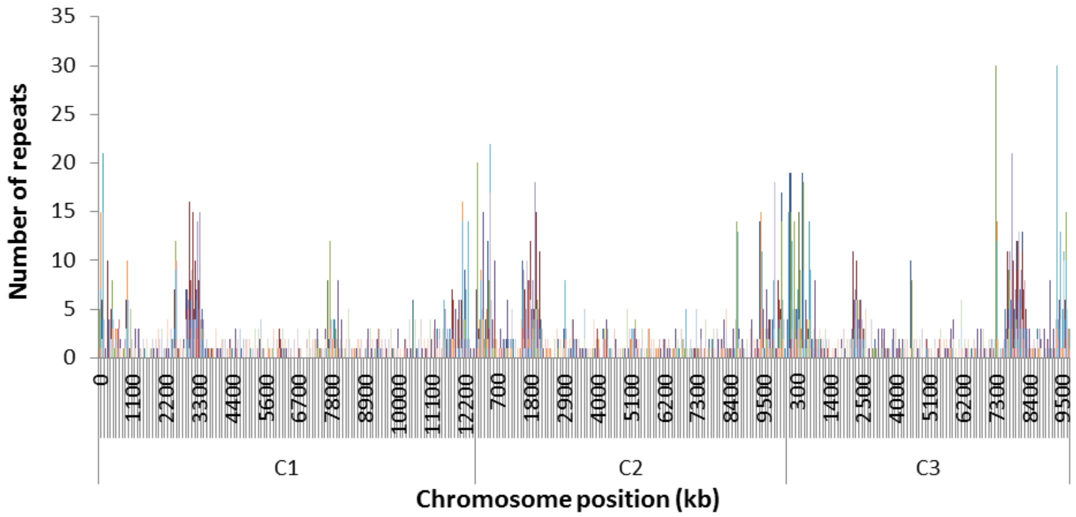

Figure S2. Distribution of species-specific repeats in the *D. coniospora* genome

Colors represent different repeat families.

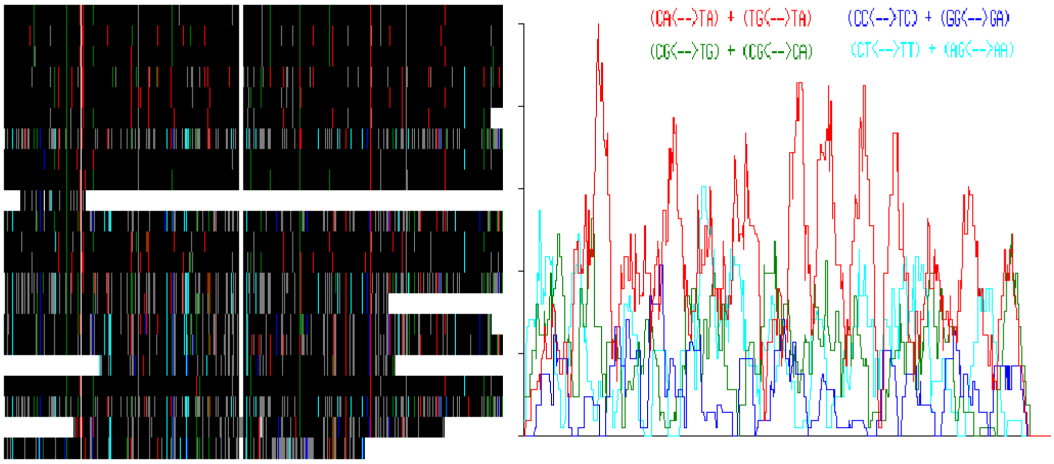

Retrotransposon DDE

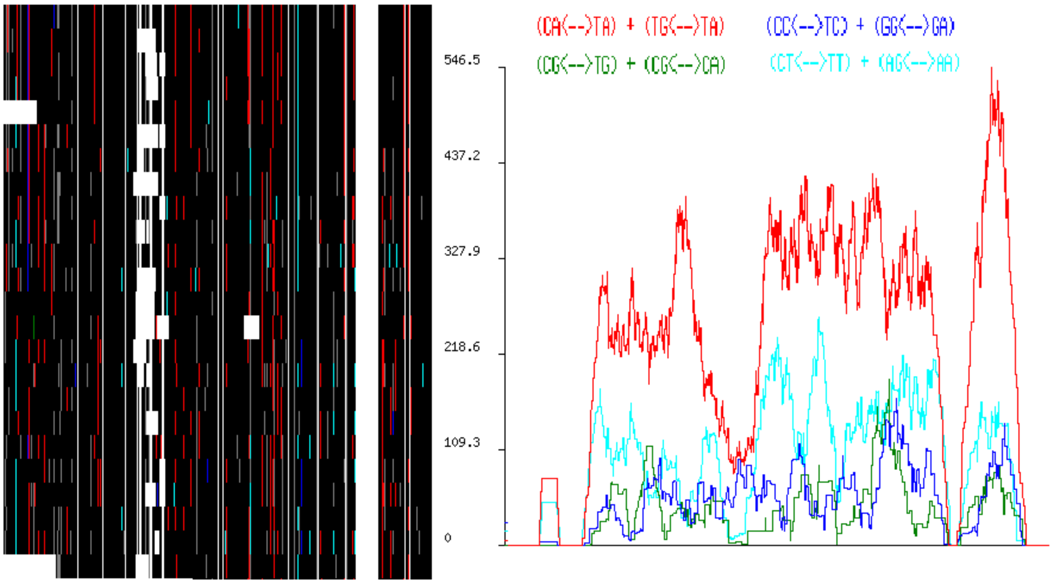

Retrotransposon *copia*

Figure S3. Repeat-induced point mutations in two retrotransposon families

Panels on the left: Repeat-induced point mutations (RIP) in the DDE and *copia* transposon families, visualized by RIPCAL[1-3]. Each lane represents one copy of a DDE or *copia* element. The nucleotide sequences of the concatenated DDE or *copia* elements are plotted on the X coordinate. Black color represents identical nucleotides. Colored bars represent selected nucleotide polymorphisms (see below); other nucleotide changes are shown in gray. Gaps are shown in white.

Panels on the right: Dinucleotide preferences in the retrotransposon families DDE (upper panel) and *copia* (lower panel). Multiple genomic regions corresponding to repeat units of the retrotransposons were concatenated.

Nucleotide polymorphisms are colored as a function of the type of RIP mutation observed. Red, CpA ⟷ TpA or TpG ⟷ TpA mutations; dark blue, CpC ⟷ TpC or GpG ⟷ GpA mutations; pale blue, CpT ⟷ TpT or ApG ⟷ ApA mutations; green, CpG ⟷ TpG or CpG ⟷ CpA mutations. CpA ⟷ TpA or TpG ⟷ TpA mutations clearly dominate in both retrotransposon families.

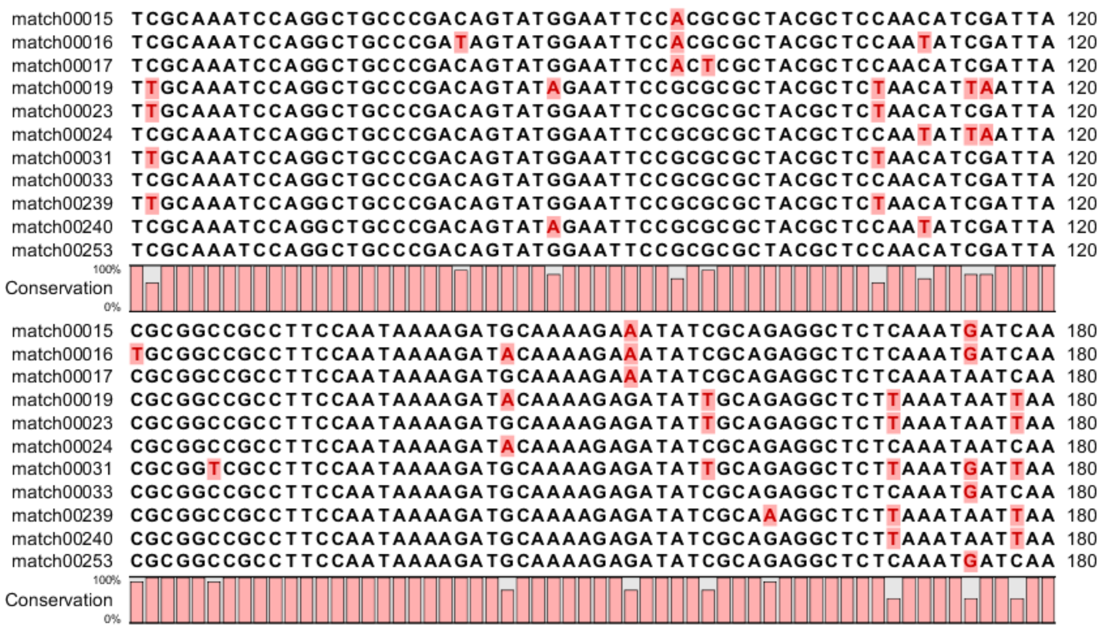

Figure S4. Nucleotide sequence alignment of 11 DDE transposases

Only partial sequences are shown with similarities exceeding 90%. Point mutations observed are either C to T or G to A (shown in *red*).

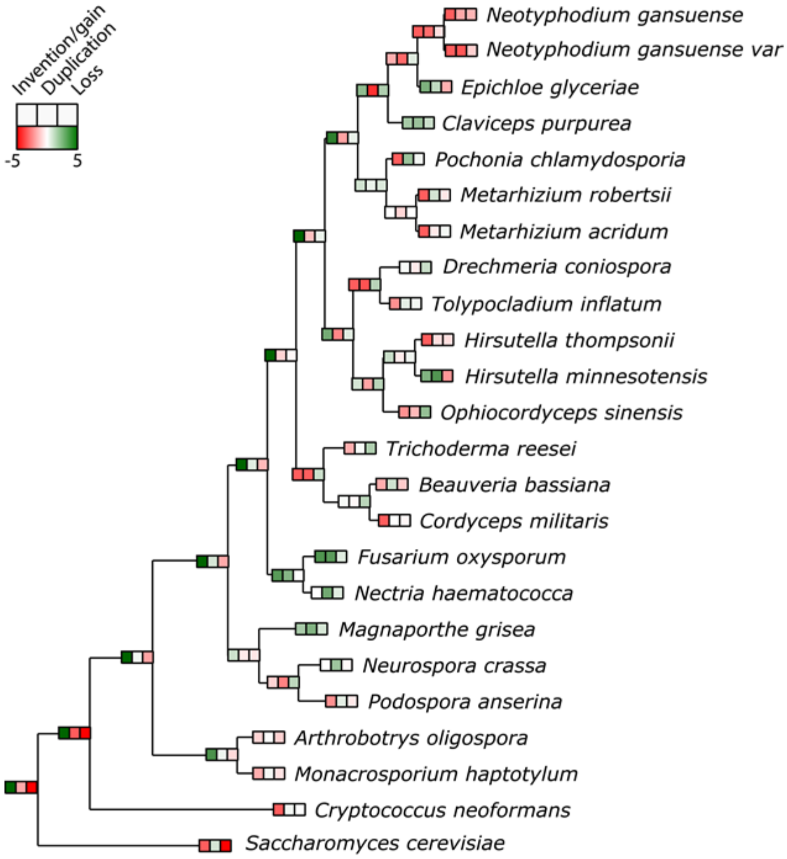

Figure S5. Gene inventions, duplications and losses mapped to the phylogeny of selected fungi

Evolutionary events were inferred by reconciliation of individual orthologous gene trees with the species tree and subsequently mapped to the different branches of the phylogenic tree. Each branch shows three boxes, one for each type of events: inventions or gene gain; duplications; and loss events. The colors of the boxes indicate whether more or less of this type of event occurred on this particular branch than in the rest of the tree: boxes are colored according to log2 (number of the type of event on this branch divided by the median of this type of events in the tree), with 4 and -4 as maximum and minimum values, respectively.

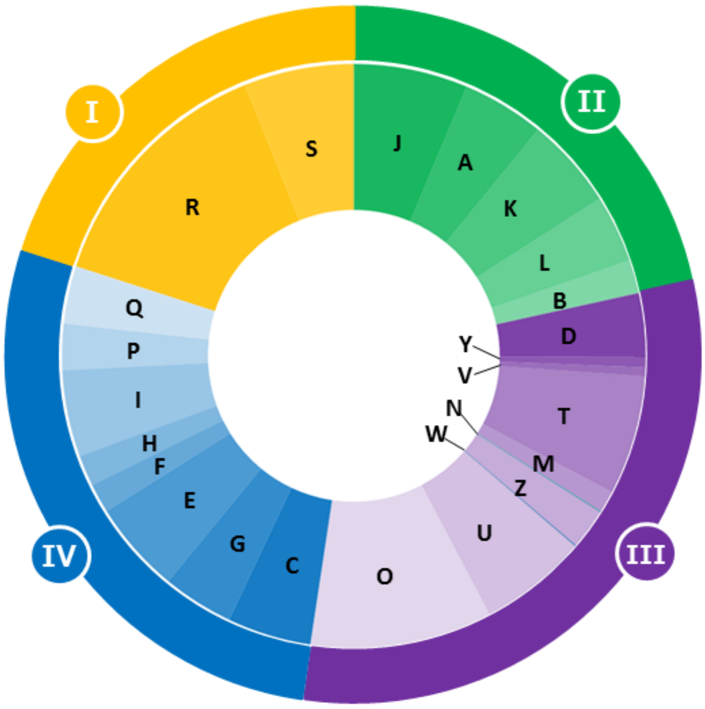

Figure S6. KOG (EuKaryotic Orthologous Groups) functional classification of the predicted *D. coniospora* proteome[4]

The inner and outer circles represent functional category and class, respectively. I, Information storage and processing; II, Cellular processes and signaling; III, Metabolism; IV, Poorly characterized; J, Translation, ribosomal structure and biogenesis; A, RNA processing and modification; K, Transcription; L, Replication, recombination and repair; B, Chromatin structure and dynamics; D, Cell cycle control, cell division, chromosome partitioning; Y, Nuclear structure; V, Defense mechanisms; T, Signal transduction mechanism; M, Cell wall/membrane/envelope biogenesis; N, Cell motility; Z, Cytoskeleton; W, Extracellular structures; U, Intracellular trafficking, secretion, and vesicular transport; O, Posttranslational modification, protein turnover, chaperones; C, Energy production and conversion; G, Carbohydrate metabolism; E, Amino acid metabolism; F, Nucleotide metabolism; H, Coenzyme metabolism; I, Lipid metabolism; P, Inorganic ion metabolism; Q Secondary metabolism; R, Prediction only; S, Function unknown.

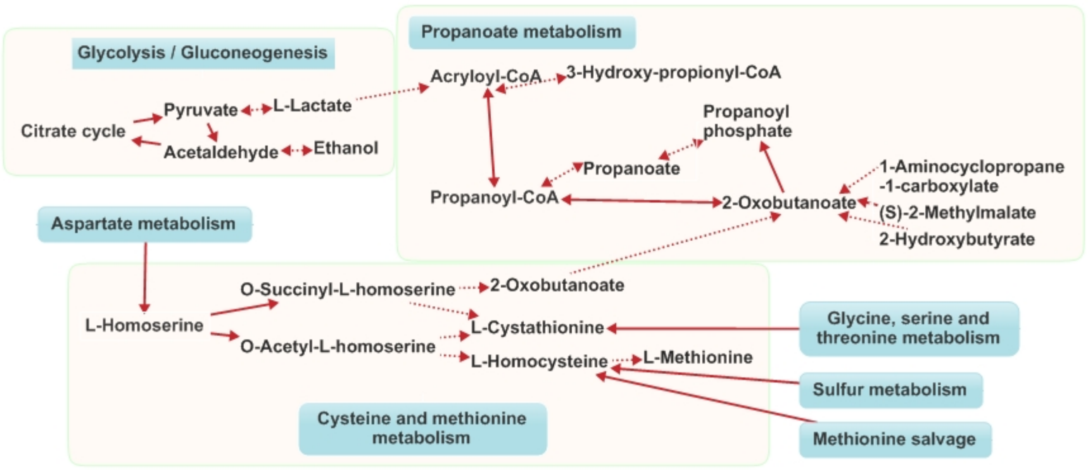

Figure S7. Selected metabolic pathways that are depleted in *D. coniospora* as compared to five closely related facultative parasitic fungi

The insect pathogens *M. anisopliae, M. acridum*, and *B. bassiana*; the nematode-trapping fungus *Ar. oligospora*; and the plant pathogen *F. oxysporum* were selected for comparison. Dotted arrows indicate metabolic steps proposed to be missing in *D. coniospora*.

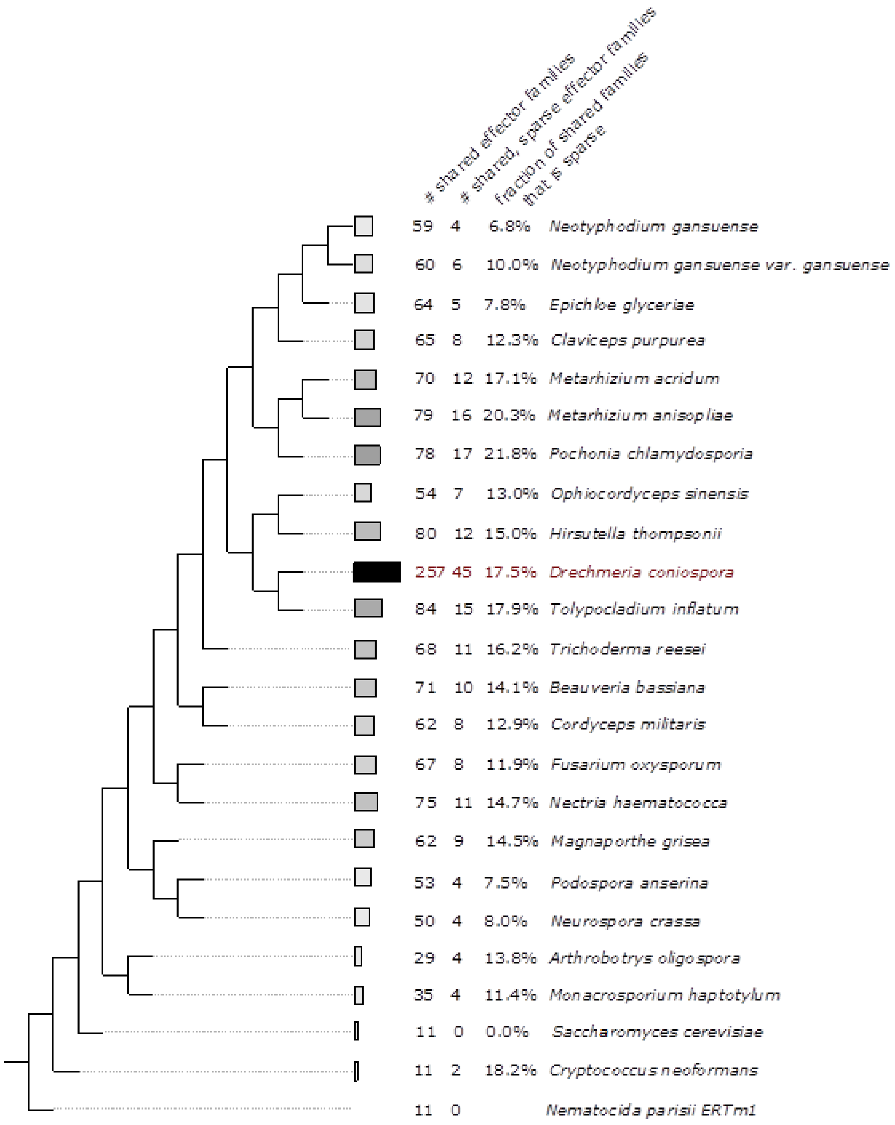

Figure S8. Analysis of the occurrence of *D. coniospora* SSP families in a set of reference fungal genomes

Of the 257 SSP families that are predicted in *D. coniospora*, 133 families also occur in other species. The widths of the bars are proportional to the numbers of SSP families that are shared by the fungi shown with *D. coniospora*. 45 of the shared SSP families that occur in *D. coniospora* and other fungi are considered ‘sparse’ families. To determine which SSP families are sparse, the ancestral species is first inferred in which a given SSP family first appeared. Next, the number of species in which that family is present is compared to the number of species under the node of invention (that is, the maximum number of species this family could occur in if it was completely retained during speciation). If an SSP family occurs in less than a third of the species it could have occurred in, the family is considered to be ‘sparse’. The fraction of sparse SSP families present in a fungal species is indicated by the grey scale of the bar, ranging from black in *D. coniospora*, in which the sparse SSP families were defined and are thus all present, to white in *S. cerevisiae* where no sparse families are found. The numbers of shared SSP families and the numbers of shared sparse SSP families are also

shown, together with the fraction of shared families that are ‘sparse’ vs. those that are simply shared.

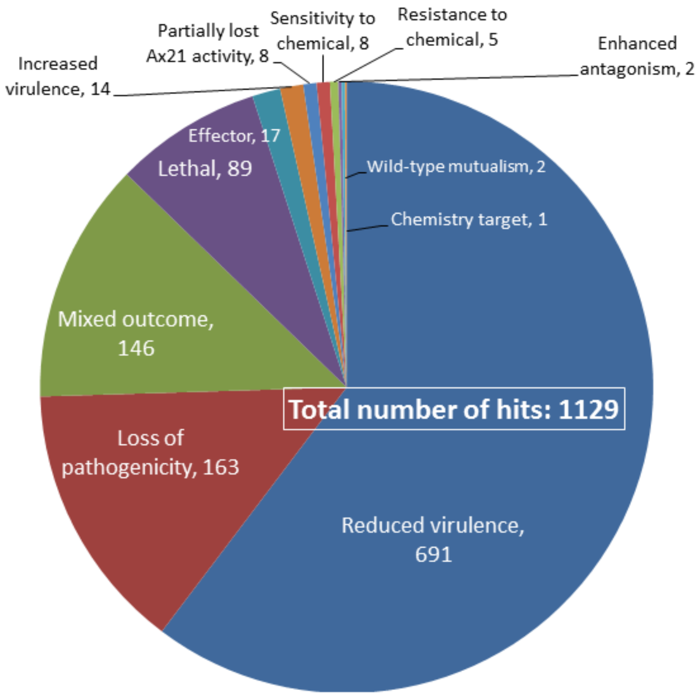

Figure S9. Predicted pathogen-host interaction (PHI) proteins in *D. coniospora* with verified participation in pathogenicity or cell viability

D. coniospora proteins orthologous to selected proteins in the Pathogen-Host Interaction (PHI) database[5,6] are shown. Only those PHI proteins were considered that affect cell viability or pathogenicity when modified or deleted.

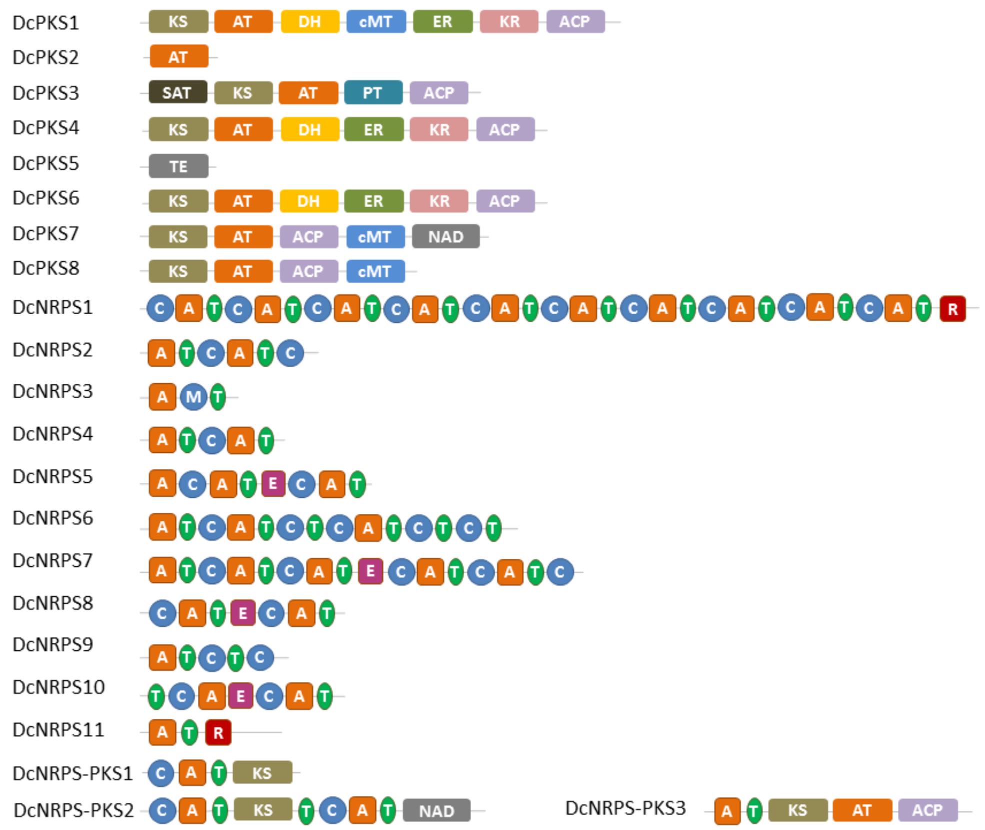

Figure S10. Domain structures of NRPS and PKS encoded in the genome of *D. coniospora*

A, adenyltransferase; ACP, acyl carrier protein; AT: acyl transferase; C, condensation domain; cMT: *C*-methyltransferase; DH: dehydratase; E, epimerase; ER: enoyl reductase; KR, ketoacyl reductase; KS, ketoacyl synthase; M, methyltransferase; NAD, NAD-binding domain; PT, product templating domain; R, reductive release domain; SAT, starter unit acyltransferase; T, thiolation domain; TE, thioesterase.

Figure S11. Phylogenetic tree of the adenylation domains of the NRPSs and NRPS-PKSs of *D. coniospora* and other related fungi

See attached PDF file. Clade annotation follows Bushley *et al*.[7] Chet, *Co. heterostrophus*. Branch support was estimated with the Shimodaira-Hasegawa test[8].

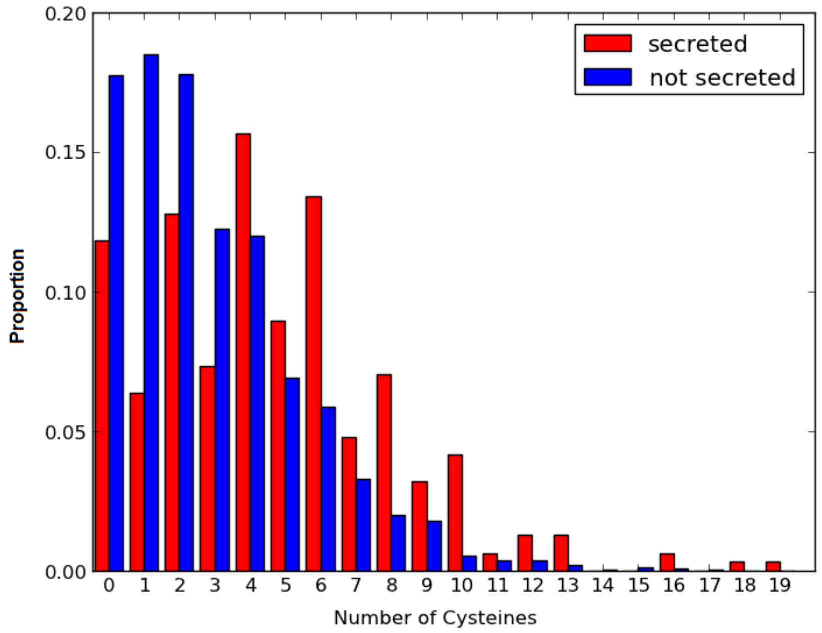

Figure S12. Distribution of the number of cysteines in small proteins in *D. coniospora*

The 312 *D. coniospora* SSPs were compared to proteins of similar size. The result shows that SSPs in *D. coniospora* contain more cysteines (P <2e-19, Kolmogorov-Smirnov test). In addition, effector proteins often have an even number of cysteines, allowing the formation of disulfide bonds. Indeed, the *D. coniospora* SSPs more frequently contain an even number of cysteines when compared to other small proteins (P <4e-07, hypergeometric test).

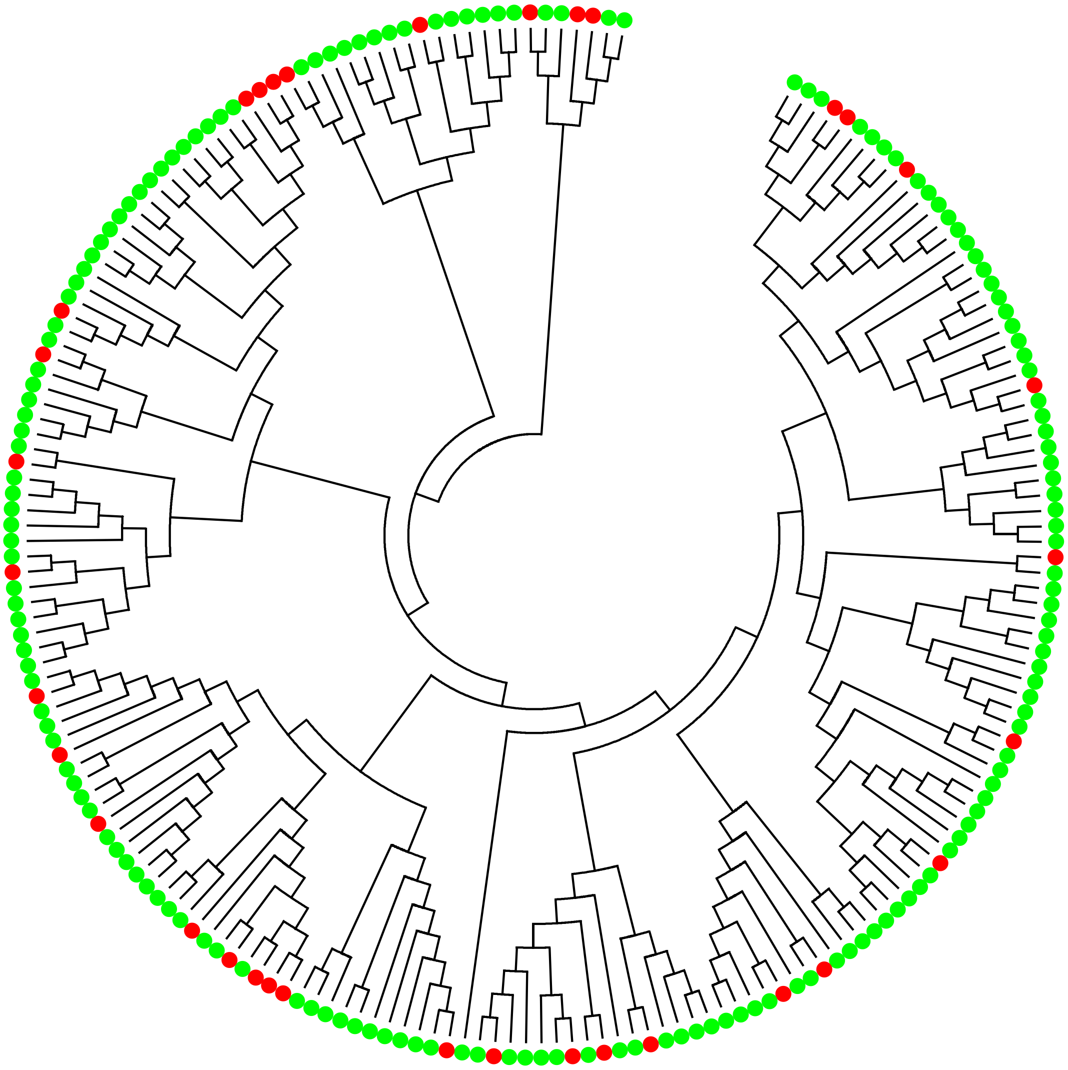

Figure S13. Homologous clustering of cytochrome P450s from *D. coniospora* (marked with red dots) and *F. oxysporum* (marked with green dots)

A.

$C_{10}H_{16}NO_2$---AHMOD---AIB---AIB---AMD---Leu/Ile---AIB---AIB---$C_7H_{16}N_3O$
Drechmerin A: $C_{61}H_{111}N_{11}O_{12}$
Calculated 1190.8492 for $[M+H]^+$ and 1212.8311 for $[M+Na]^+$

$C_{10}H_{16}NO_2$---dehydro-AHMOD---AIB---AIB---AMD---Leu/Ile---AIB---AIB---$C_7H_{16}N_3O$
Dehydro-drechmerin A: $C_{61}H_{109}N_{11}O_{11}$
Calculated 1172.8386 for $[M+H]^+$ and 1194.8206 for $[M+Na]^+$

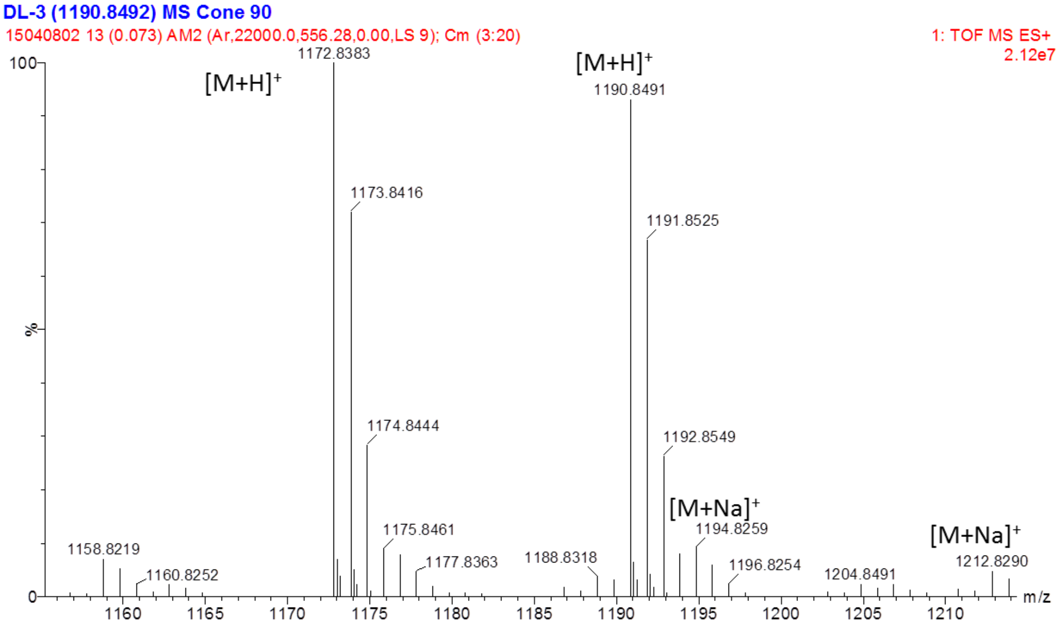

B.

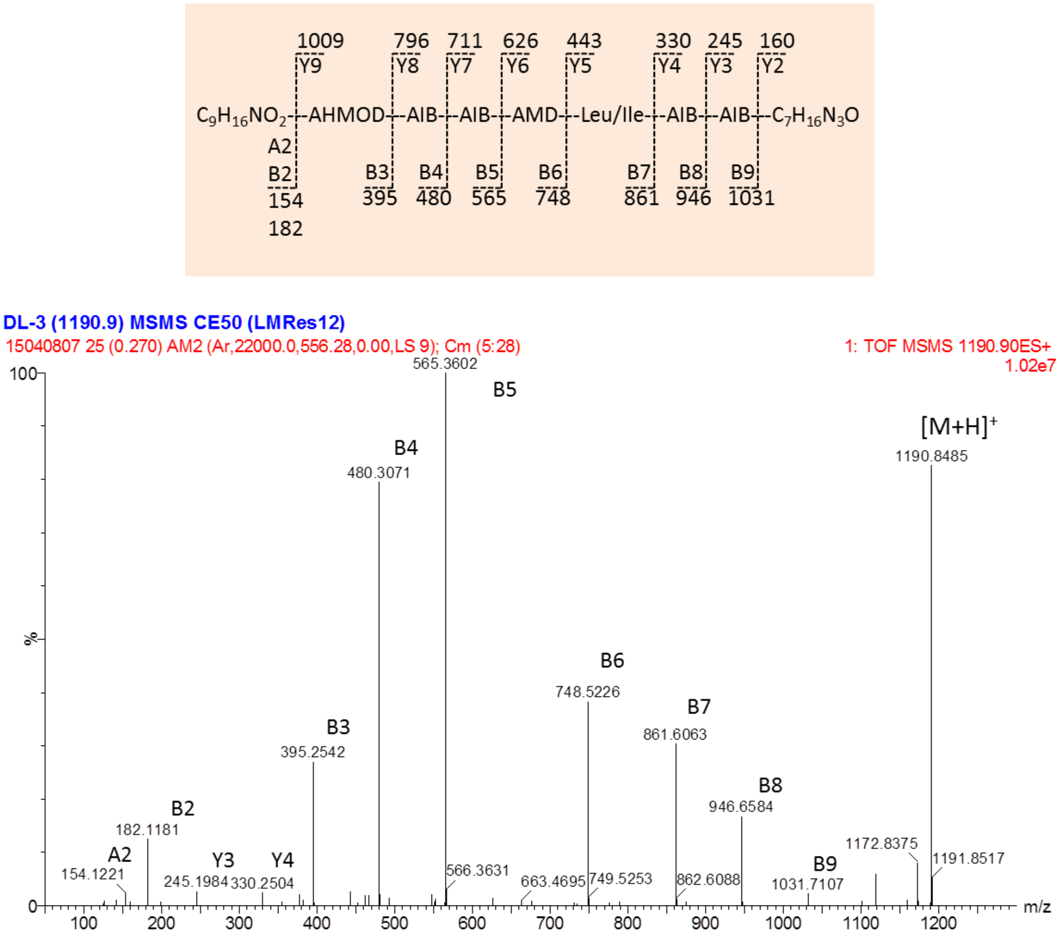

1009 796 711 626 443 330 245 160
Y9 Y8 Y7 Y6 Y5 Y4 Y3 Y2
C9H16NO2--AHMOD--AIB--AIB--AMD--Leu/Ile--AIB--AIB--C7H16N3O
A2
B2 B3 B4 B5 B6 B7 B8 B9
154 395 480 565 748 861 946 1031
182
DL-3 (1190.9) MSMS CE50 (LMRes12)
15040807 25 (0.270) AM2 (Ar,22000.0,556.28,0.00,LS 9); Cm (5:28)
1: TOF MSMS 1190.90ES+
1.02e7
565.3602
B5
B4
480.3071
[M+H]+
1190.8485
B6
748.5226
B7
861.6063
B3
395.2542
B8
946.6584
B2
182.1181
1172.8375
A2
Y3
Y4
1191.8517
B9
154.1221
245.1984
330.2504
566.3631
663.4695
749.5253
862.6088
1031.7107
%
m/z

C.

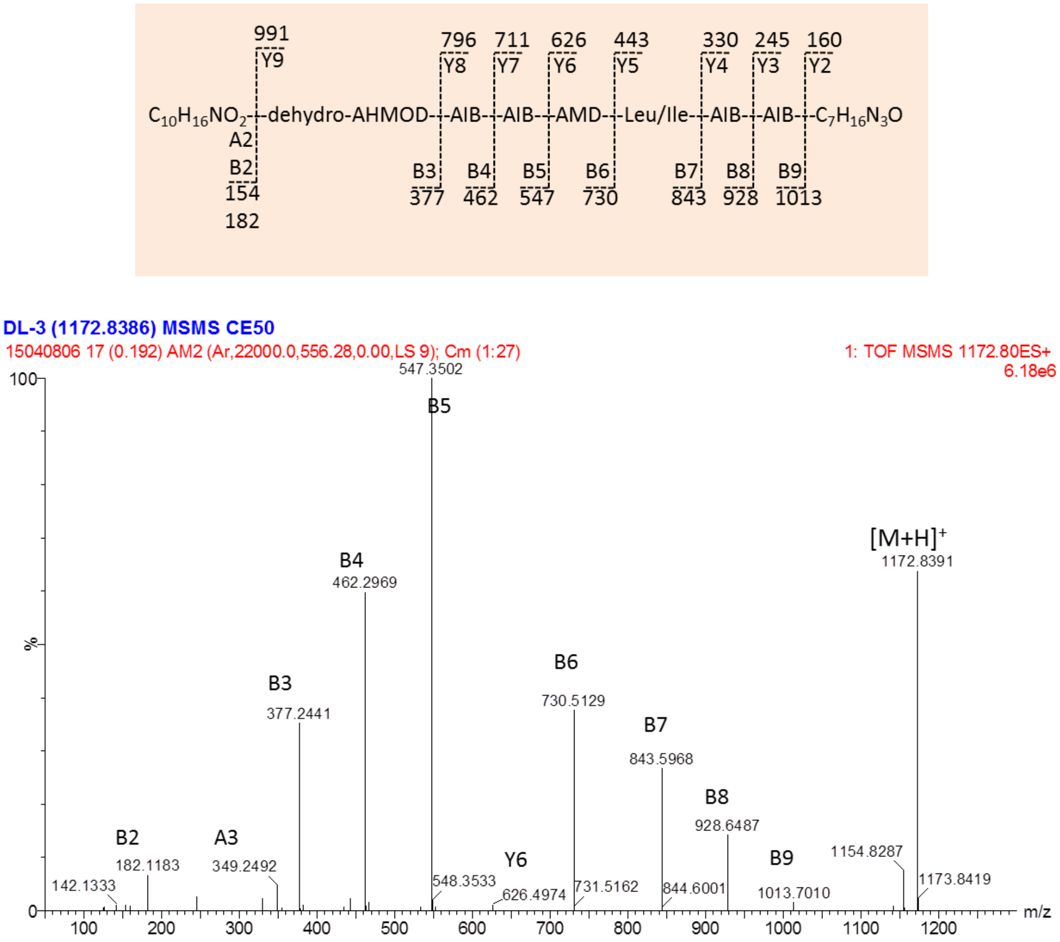

991
Y9
796 711 626 443 330 245 160
Y8 Y7 Y6 Y5 Y4 Y3 Y2
C10H16NO2--dehydro-AHMOD--AIB--AIB--AMD--Leu/Ile--AIB--AIB--C7H16N3O
A2
B2
154
182
B3 B4 B5 B6 B7 B8 B9
377 462 547 730 843 928 1013
DL-3 (1172.8386) MSMS CE50
15040806 17 (0.192) AM2 (Ar,22000.0,556.28,0.00,LS 9); Cm (1:27)
1: TOF MSMS 1172.80ES+
6.18e6
547.3502
B5
B4
462.2969
B3
377.2441
B6
730.5129
B7
843.5968
B8
928.6487
B9
1013.7010
[M+H]+
1172.8391
1154.8287
1173.8419
B2
182.1183
A3
349.2492
142.1333
548.3533
Y6
626.4974
731.5162
844.6001
%
m/z

D.

$C_{10}H_{16}NO_2$---AHMOD---ALA---AIB---AMD---Leu/Ile---AIB---AIB---$C_7H_{16}N_3O$
Drechmerin B: $C_{60}H_{109}N_{11}O_{12}$
Calculated 1176.8335 for $[M+H]^+$ and 1198.8155 for $[M+Na]^+$

$C_{10}H_{16}NO_2$---dehydro-AHMOD---ALA---AIB---AMD---Leu/Ile---AIB---AIB---$C_7H_{16}N_3O$
Dehydro-drechmerin B: $C_{60}H_{107}N_{11}O_{11}$
Calculated 1158.8230 for $[M+H]^+$ and 1180.8049 for $[M+Na]^+$

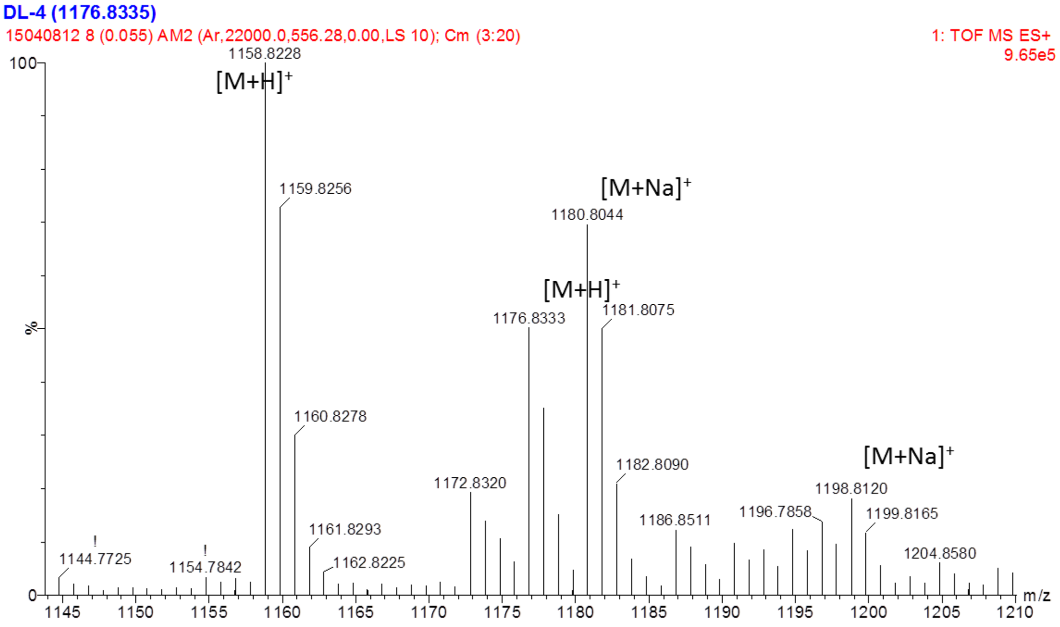

E.

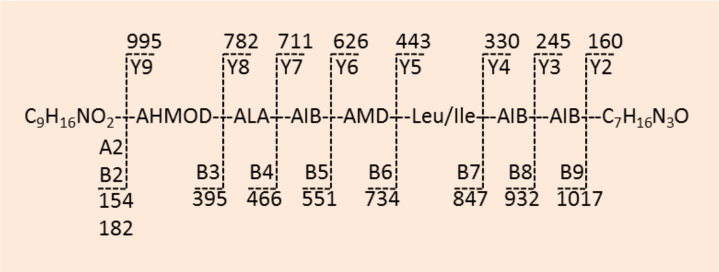
995 782 711 626 443 330 245 160
Y9 Y8 Y7 Y6 Y5 Y4 Y3 Y2
C9H16NO2--AHMOD--ALA--AIB--AMD--Leu/Ile--AIB--AIB--C7H16N3O
A2 B2 B3 B4 B5 B6 B7 B8 B9
154 395 466 551 734 847 932 1017
182

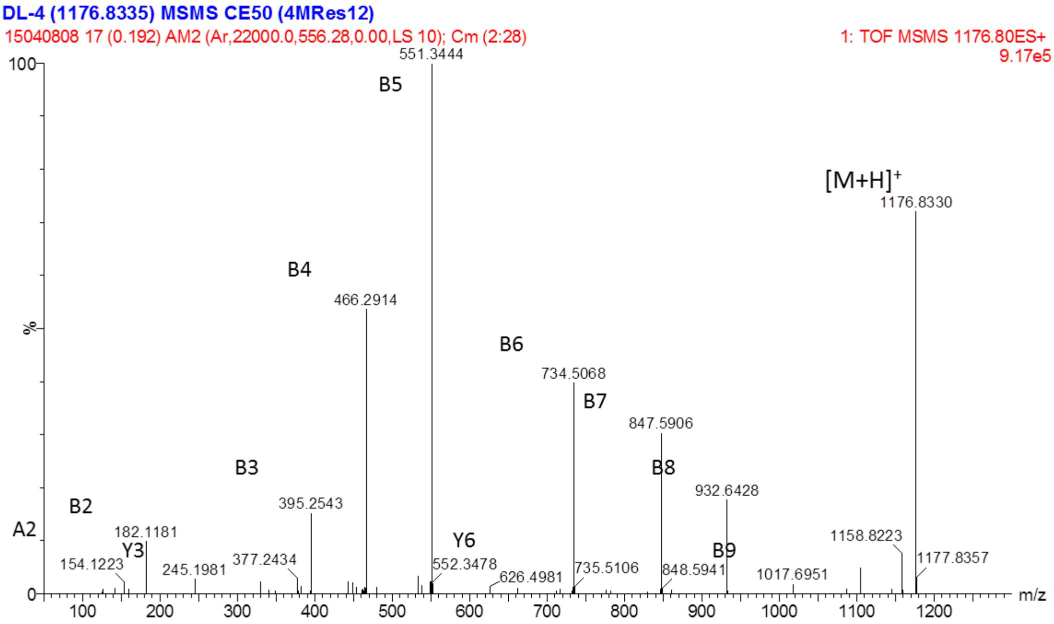
DL-4 (1176.8335) MSMS CE50 (4MRes12)
15040808 17 (0.192) AM2 (Ar,22000.0,556.28,0.00,LS 10); Cm (2:28)
1: TOF MSMS 1176.80ES+
9.17e5
B5
551.3444
[M+H]+
1176.8330
B4
466.2914
B6
734.5068
B7
847.5906
B3
395.2543
B8
932.6428
B2
A2
182.1181
Y3
154.1223
245.1981
377.2434
Y6
552.3478
626.4981
735.5106
848.5941
B9
1017.6951
1158.8223
1177.8357
%
m/z

F.

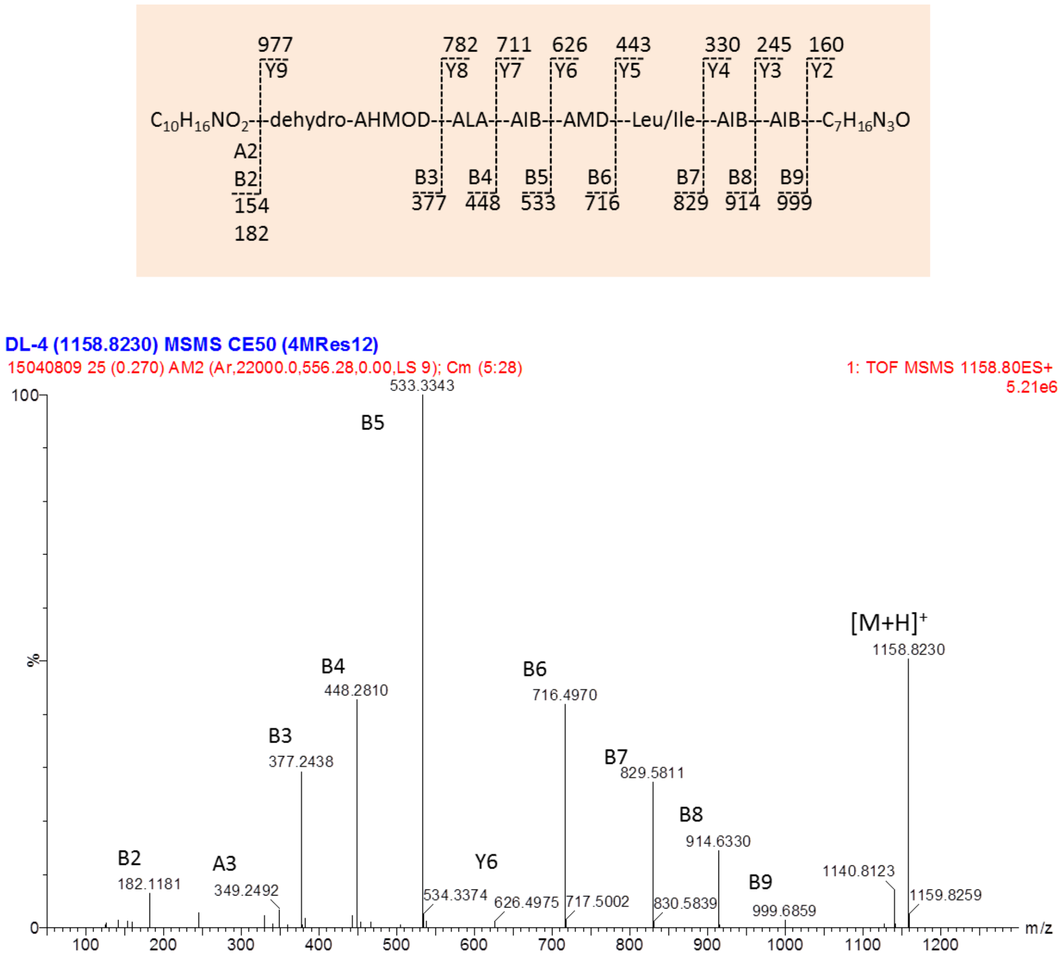

Figure S14. Mass spectrometric characterization of the structures of the main drechmerin congeners

A. Accurate MS spectrum (XEVO G2) of drechmerin A showing pseudomolecular ions for both drechemerin A (calculated: 1190.8492 for $[M+H]^+$ and 1212.8311 for $[M+Na]^+$) and dehydro-drechmerin A, the product resulting from dehydration of the AHMOD unit (calculated: 1172.8386 for $[M+H]^+$ and 1194.8206 for $[M+Na]^+$). **B.** Accurate MS/MS spectrum (XEVO G2) showing product ions of the drechmerin A parent ion at *m/z* 1190.8. **C.** Accurate MS/MS spectrum (XEVO G2) showing product ions of dehydro-drechmerin A parent ion at *m/z* 1172.8. **D.** Accurate MS spectrum showing pseudomolecular ions for drechemerin B (calculated: 1176.8335 for $[M+H]^+$ and 1198.8155 for $[M+Na]^+$) and dehydro-drechemerin B, the product resulting from dehyration of the AHMOD unit, (calculated: 1158.8230 for $[M+H]^+$ and 1180.8049 for $[M+Na]^+$). **E.** Accurate MS/MS spectrum (XEVO G2) showing product ions of drechmerin B parent ion at 1176.80 Da. **F.** Accurate MS/MS spectrum (XEVO G2) showing product ions of dehydro-drechmerin B parent ion at 1158.8 Da. Fragment ions on panels B, C, E and F are labelled using standard nomenclature[9]. "Y" ions that were too small to be annotated at the scale shown are listed in Table S22.

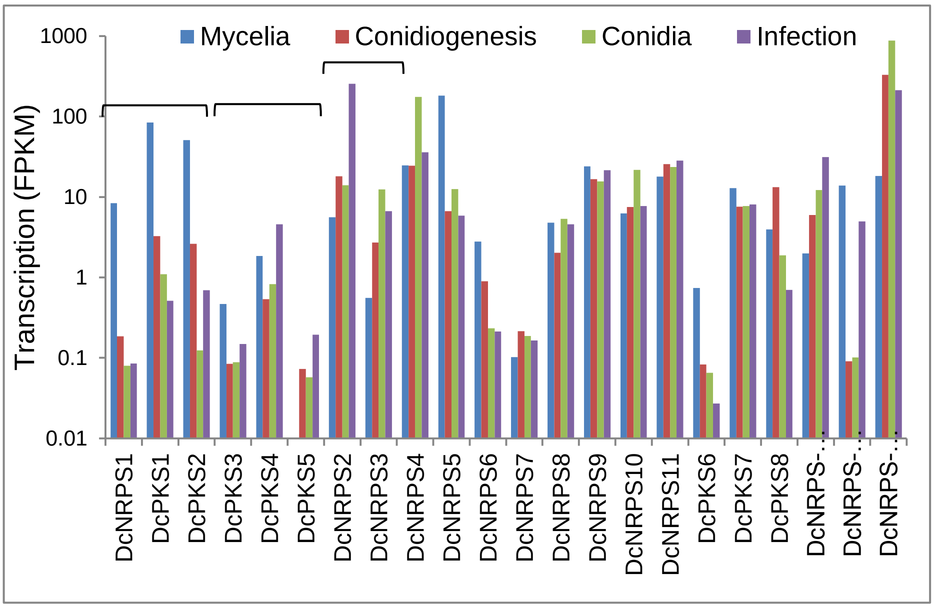

Figure S15. Transcription levels of the NRPS, PKS and NRPS-PKS genes of *D. coniospora* during the four life stages

The genes that are parts of the same putative gene cluster are marked with black brackets.

A.

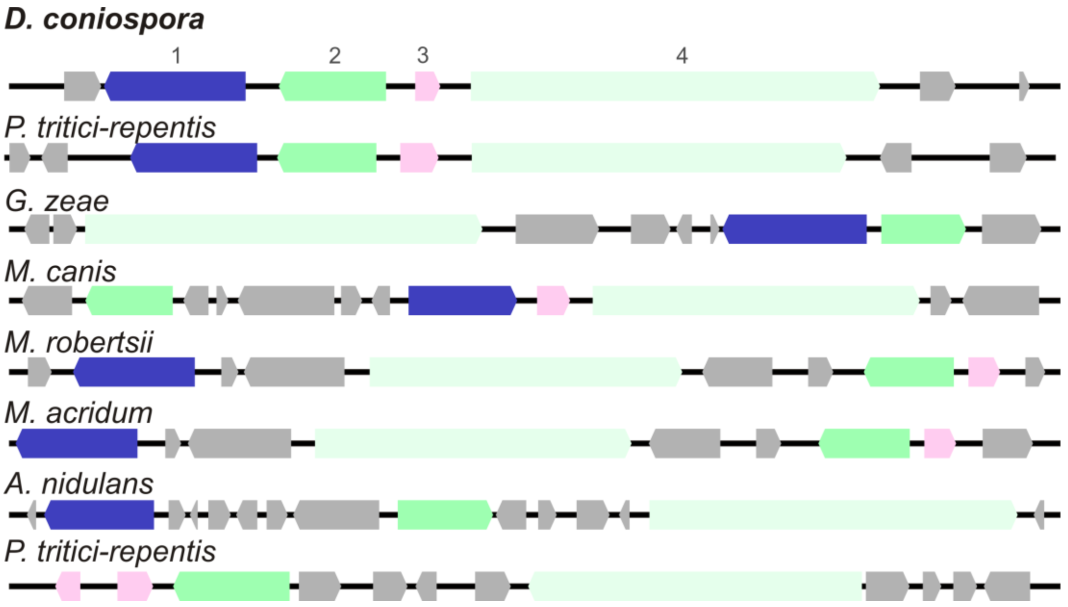
D. coniospora
1
2
3
4
P. tritici-repentis
G. zeae
M. canis
M. robertsii
M. acridum
A. nidulans
P. tritici-repentis

B.

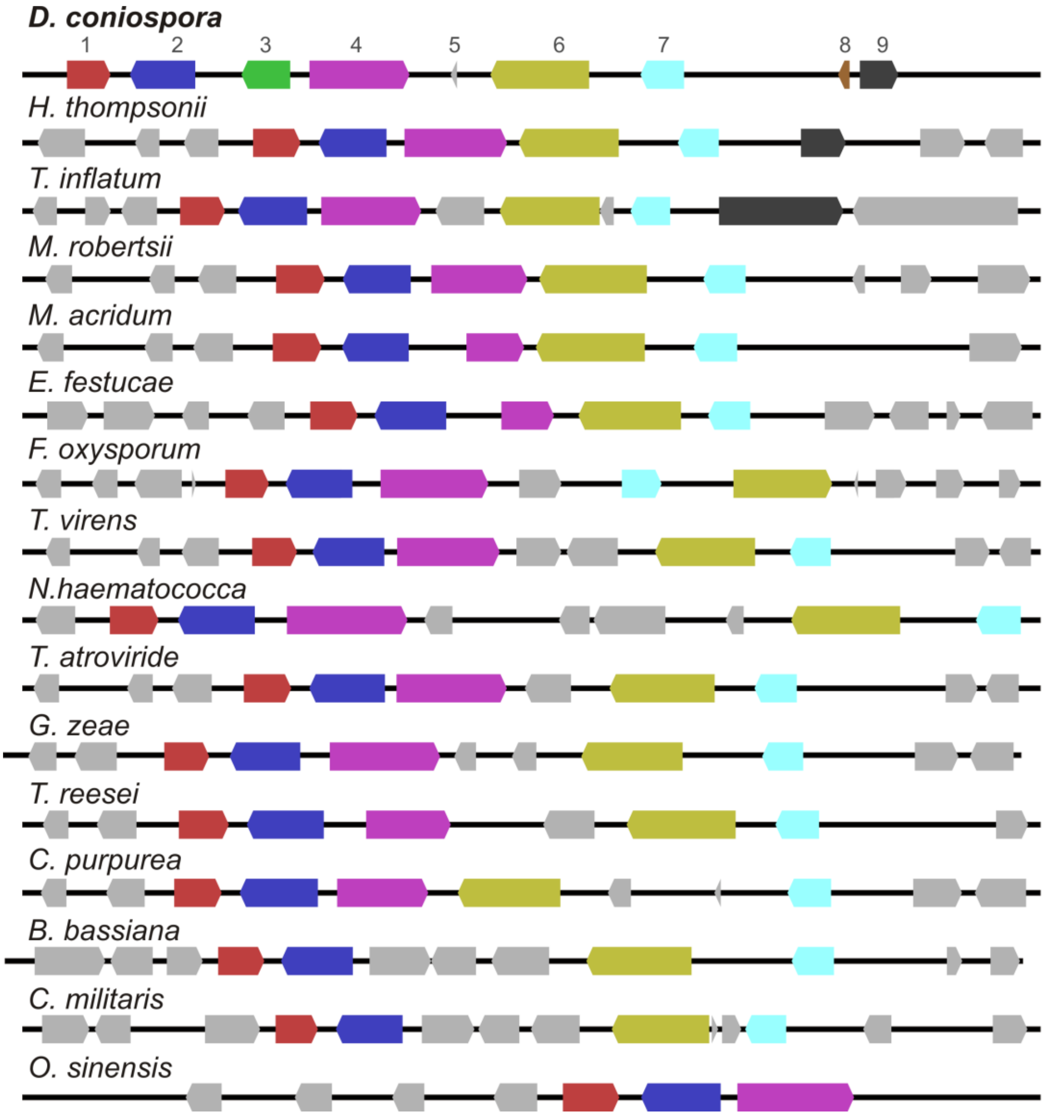

D. coniospora
1
2
3
4
5
6
7
8
9
H. thompsonii
T. inflatum
M. robertsii
M. acridum
E. festucae
F. oxysporum
T. virens
N.haematococca
T. atroviride
G. zeae
T. reesei
C. purpurea
B. bassiana
C. militaris
O. sinensis

C.

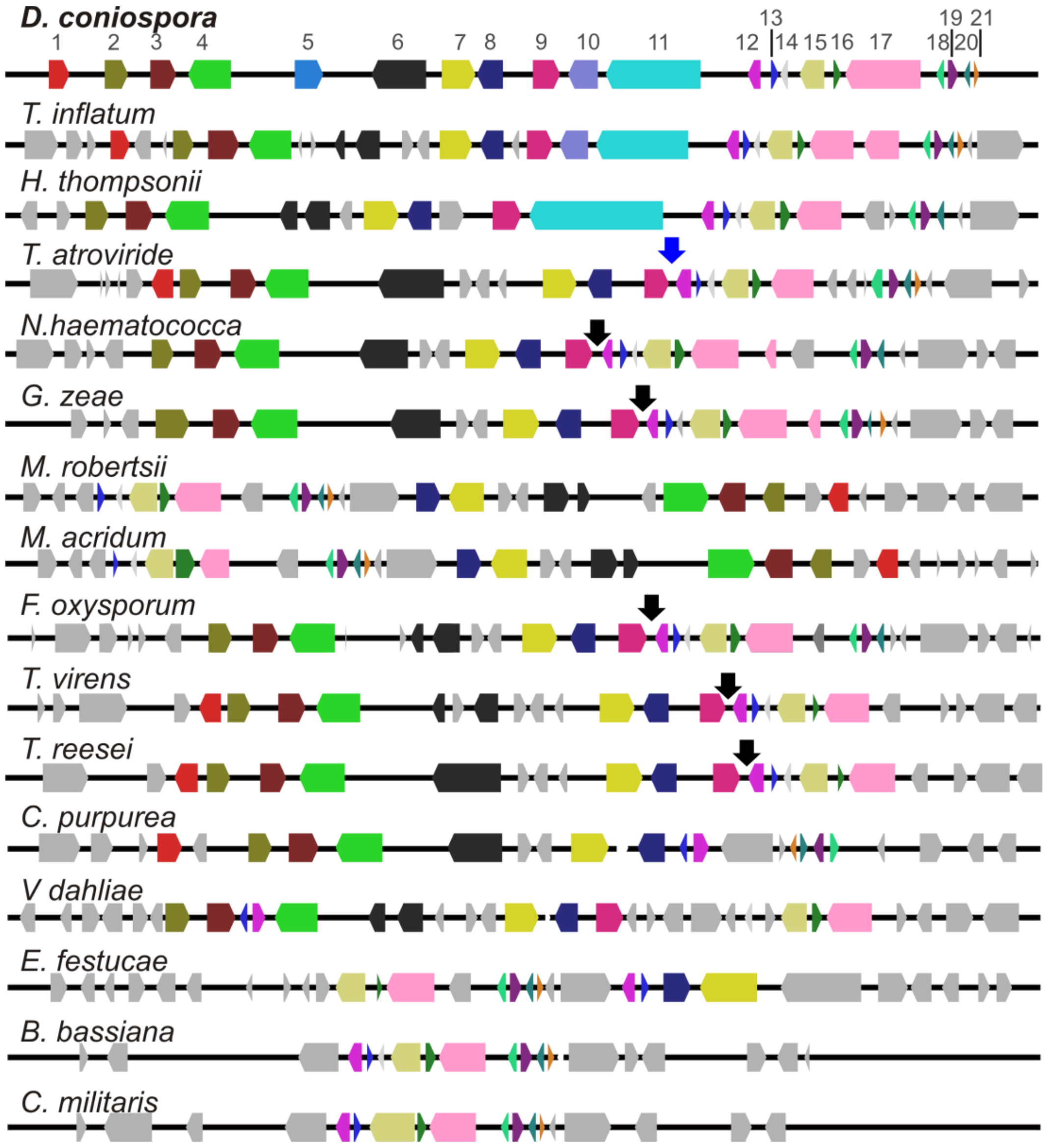

Figure S16. MultiGeneBlast alignments of selected secondary metabolite biosynthetic clusters in *D. coniospora* with syntenic clusters in other fungi

A. The DcNRPS7 locus (1: DCS_00044; 2: DCS_0045; 3: DCS_0046; 4: DCS_0047=DcNRPS7). **B.** The DcNRPS11 locus (1-9: DCS_03471-DCS_03479; where 6: DCS_03476=DcNRPS11). **C.** The DcNRPS4 locus (1-21: DCS_07691- DCS_07711; where 11: DCS_07701=DcNRPS4). Fat arrows indicate the putative insertion point of the DcNRPS4 cluster.

References

1 Hane, J. K. & Oliver, R. P. RIPCAL: a tool for alignment-based analysis of repeat-induced point mutations in fungal genomic sequences. **9**, doi:10.1186/1471-2105-9-478 (2008).

2 Gao, Q. A. *et al.* Genome sequencing and comparative transcriptomics of the model entomopathogenic fungi *Metarhizium anisopliae* and *M. acridum*. *PLoS Genet.* **7**, 18, doi:e100126410.1371/journal.pgen.1001264 (2011).

3 Zhang, Y. *et al.* Specific adaptation of Ustilaginoidea virens in occupying host florets revealed by comparative and functional genomics. **5**, doi:10.1038/ncomms4849 (2014).

4 Muller, J. *et al.* eggNOG v2.0: extending the evolutionary genealogy of genes with enhanced non-supervised orthologous groups, species and functional annotations. **38**, D190-D195, doi:10.1093/nar/gkp951 (2010).

5 Winnenburg, R. *et al.* PHI-base: a new database for pathogen host interactions. *Nucleic acids research* **34**, D459-464, doi:10.1093/nar/gkj047 (2006).

6 Winnenburg, R. *et al.* PHI-base update: additions to the pathogen-host interaction database. *Nucleic Acids Res* **36**, D572-D576, doi:10.1093/nar/gkm858 (2008).

7 Bushley, K. E. & Turgeon, B. G. Phylogenomics reveals subfamilies of fungal nonribosomal peptide synthetases and their evolutionary relationships. *BMC Evol Biol* **10**, doi:10.1186/1471-2148-10-26 (2010).

8 Shimodaira, H. & Hasegawa, M. *Multiple Comparisons of Log-Likelihoods with Applications to Phylogenetic Inference*.

9 Roepstorff, P. & Fohlman, J. Proposal for a common nomenclature for sequence ions in mass spectra of peptides. *Biomedical mass spectrometry* **11**, 601, doi:10.1002/bms.1200111109 (1984).

10 Lai, Y. L. *et al.* Comparative genomics and transcriptomics analyses reveal divergent lifestyle features of nematode endoparasitic fungus *Hirsutella minnesotensis*. *Genome Biol. Evol.* **6**, 3077-3093, doi:10.1093/gbe/evu241 (2014).

11 Bushley, K. E. *et al.* The genome of *Tolypocladium inflatum*: Evolution, organization, and expression of the cyclosporin biosynthetic gene cluster. *PLoS Genet* **9**, doi:10.1371/journal.pgen.1003496 (2013).

12 Ward, E. *et al.* The Pochonia chlamydosporia Serine Protease Gene vcp1 Is Subject to Regulation by Carbon, Nitrogen and pH: Implications for Nematode Biocontrol. *Plos One* **7**, doi:10.1371/journal.pone.0035657 (2012).

13 Yang, J. K. *et al.* Genomic and proteomic analyses of the fungus *Arthrobotrys oligospora* provide insights into nematode-trap formation. *Plos Pathog* **7**, e1002179, doi:10.1371/journal.ppat.1002179 (2011).

14 Xiao, G. *et al.* Genomic perspectives on the evolution of fungal entomopathogenicity in *Beauveria bassiana*. **2**, 483, doi:10.1038/srep00483 (2012).

15 Schardl, C. L. *et al.* Plant-symbiotic fungi as chemical engineers: multi-genome analysis of the clavicipitaceae reveals dynamics of alkaloid loci. *PLoS Genet.* **9**, e1003323, doi:10.1371/journal.pgen.1003323 (2013).

16 Zheng, P. *et al.* Genome sequence of the insect pathogenic fungus Cordyceps militaris, a valued traditional Chinese medicine. *Genome Biol.* **12**, R116, doi:10.1186/gb-2011-12-11-r116 (2011).

17 Loftus, B. J. *et al.* The genome of the basidiomycetous yeast and human pathogen Cryptococcus neoformans. *Science (New York, N.Y.)* **307**, 1321-1324, doi:10.1126/science.1103773 (2005).

18 Ma, L. J. *et al.* Comparative genomics reveals mobile pathogenicity chromosomes in Fusarium. *Nature* **464**, 367-373, doi:10.1038/nature08850 (2010).

19 Dean, R. A. *et al.* The genome sequence of the rice blast fungus Magnaporthe grisea. **434**, 980-986, doi:10.1038/nature03449 (2005).

20 Pattemore, J. A. *et al.* The genome sequence of the biocontrol fungus Metarhizium anisopliae and comparative genomics of Metarhizium species. **15**, 660, doi:10.1186/1471-2164-15-660 (2014).

21 Meerupati, T. *et al.* Genomic mechanisms accounting for the adaptation to parasitism in nematode-trapping fungi. *PLoS Genet.* **9**, 20, doi:10.1371/journal.pgen.1003909 (2013).

22 Coleman, J. J. *et al.* The genome of Nectria haematococca: contribution of supernumerary chromosomes to gene expansion. *PLoS Genet.* **5**, e1000618, doi:10.1371/journal.pgen.1000618 (2009).

23 Hu, X. *et al.* Genome survey uncovers the secrets of sex and lifestyle in caterpillar fungus. *Chin. Sci. Bull.* **58**, 2846-2854, doi:10.1007/s11434-013-5929-5 (2013).

24 Larriba, E. *et al.* Sequencing and functional analysis of the genome of a nematode egg-parasitic fungus, *Pochonia chlamydosporia*. *Fungal genetics and biology : FG & B* **65**, 69-80, doi:http://dx.doi.org/10.1016/j.fgb.2014.02.002 (2014).

25 Clutterbuck, A. J. Genomic evidence of repeat-induced point mutation (RIP) in filamentous ascomycetes. *Fungal Genet. Biol.* **48**, 306-326, doi:10.1016/j.fgb.2010.09.002 (2011).

26 Treu, L. *et al.* The impact of genomic variability on gene expression in environmental Saccharomyces cerevisiae strains. *Environmental microbiology* **16**, 1378-1397, doi:10.1111/1462-2920.12327 (2014).

27 Martinez, D. *et al.* Genome sequencing and analysis of the biomass-degrading fungus Trichoderma reesei (syn. Hypocrea jecorina). **26**, 553-560, doi:10.1038/nbt1403 (2008).

Supplementary Tables

Insights into adaptations to a near-obligate nematode endoparasitic lifestyle from the finished genome of *Drechmeria coniospora*

Liwen Zhang, Zhengfu Zhou, Qiannan Guo, Like Fokkens, Márton Miskei, István Pócsi, Wei Zhang, Ming Chen, Lei Wang, Yamin Sun, Bruno G. G. Donzelli, Donna M. Gibson, David R. Nelson, Jian-Guang Luo, Martijn Rep, Hang Liu, Shengnan Yang, Jing Wang, Stuart B. Krasnoff, Yuquan Xu*, István Molnár*, and Min Lin*

Contents

Table S1. Statistics of the *D. coniospora* genome assembly 33

Table S2. Comparison of the features of the *D. coniospora* genome with those of closely related ascomycetes and other nematophagous fungi.. 34

Table S3. Repetitive elements in the *D. coniospora* genome 35

Table S4. Length distribution of the repetitive elements in the *D. coniospora* genome 36

Table S5. *D. coniospora* genes orthologous to those that are involved in the asexual sporulation of *A. nidulans* and *Fusarium spp.*.. 37

Table S6. Distribution of differentially expressed genes in KOG categories amongst *in vitro* growth stages... 38

Table S7. Comparative analysis of the predicted proteomes of fungi with various econutritional lifestyles 39

Table S8. Functional classification of *D. coniospora* and *M. robertsii* proteins using KEGG Metabolic Pathway annotations.. 40

Table S9. Characteristics of fungal genomes used in this study... 41

Table S10. Comparison of the numbers of genes encoding plant biomass degradation enzymes in *D. coniospora* with those of selected plant or insect pathogenic fungi 43

Table S11. The category of selected genes present in five facultative endoparasites but missing from the *D. coniospora* genome 44

Table S12. Number of predicted *D. coniospora* proteins featuring various PFAM domains (number > 3).. 46

Table S13. Stress response proteins encoded in the *D. coniospora* genome 47

Table S14. Functional domain annotation of *D. coniospora* SSPs...................................... 48

Table S15. *D. coniospora* protein families potentially involved in pathogenicity 49

Table S16. NRPS, PKS and NRPS-PKS genes and the corresponding gene clusters in *D. coniospora*.. 50

Table S16A. *D. coniospora* NRPS, PKS and NRPS-PKS genes and their transcript abundances in different life stages.. 50

Table S16B. *D. coniospora* secondary metabolite biosynthetic loci and the transcript abundances of the genes (in FPKM) in different life stages.. 50

Table S17. Cytochrome P450s (CYPs) of *D. coniospora* and their expression in different life stages... 51

Table S18. *D. coniospora* orthologs of proteins involved in iron metabolism in other fungi 53

Table S19. Accurate MS/MS data for drechmerin congeners (see Fig S14) 54
References 57

Table S1. Statistics of the *D. coniospora* genome assembly

Feature	NGS	NGS + PacBio	NGS + PacBio + Optical mapping
Largest scaffold length	1,751,344	9,383,697	12,323,145
No. of large scaffolds	483	37	3
Bases in large scaffolds	32,725,659	32,192,957	32,800,123
N50 scaffold	27	3	N/A
N50 length	398,529	4,137,305	N/A
N90 scaffold	95	8	N/A
N90 length	81,670	1,535,228	N/A
GC content	55.1	55.0	55.0
N%	3.9	1.9	1.9

N/A, not applicable

Table S2. Comparison of the features of the *D. coniospora* genome with those of closely related ascomycetes and other nematophagous fungi

Feature	DCS	HIM[10]	TIN[11]	PCH[12]	AOL[13]	MRO[2]	MAC[2]
Fold coverage	457.9	128	ND	136	36.6	100	107
Contig N50 (kb)	N/A	ND	ND	ND	576	ND	ND
Scaffold number	3	967	101	901	215	176	241
Scaffold N50 (Mb)	N/A	0.3824	1.5	0.225	2.0	2	0.329
Genome size (Mb)	32.5	51.4	30.4	41.2	40.1	39.0	38.05
Predicted proteins	8,281	12,702	9,998	12,122	11,479	10,582	9,849
GC content (%)	55.0	52.1	58	49.9	44.5	51.5	49.9
Repetitive DNA (%)	12.5	35.99	1.2	0.5	0.5	1.0	1.5
Gene density (gene/Mb)	254.8	247.1	329.4	294	286.5	271.3	259.2
Total length of genes (Mb)	12.8	20.6	16.7	17.3	19.4	ND	16.5
Total length of exons (Mb)	12.1	18.95	15.0	ND	17.2	ND	14.6
Nonrepetitive intergenic DNA (%)	27	ND	ND	ND	ND	ND	ND
Average gene size (kb)	2.3	1.624	1.7	1.4	1.7	ND	1.7
Average number of introns per gene	2.0	ND	2.2	ND	2.2	ND	1.7
Average intron length (bp)	42	130	77	ND	89	ND	112
tRNA genes	125	145	112	45	145	141	122

N/A, not applicable. ND, data not available. DCS, *D. coniospora*; HIM, *H. minnesotensis*; TIN, *T. inflatum*; PCH, *P. chlamydosporia*; AOL, *A. oligospora*; MRO, *M. robertsii*; MAC, *M. acridum*.

Table S3. Repetitive elements in the *D. coniospora* genome

**Type**	**Number of elements**	**Total length (bp)**	**Percent of genome**
**Retrotransposons (Class I)**			
DDE	55	48,537	0.15%
gypsy	253	220,389	0.67%
Copia	211	325,032	0.99%
LINE	98	102,075	0.31%
LTR_Roo	1	447	0.00%
**DNA transposons (Class II)**			
cacta	75	7,914	0.02%
hAT	8	2,688	0.01%
helitron	2	1,434	0.00%
mariner	16	7,818	0.02%
mariner_ant1	10	5,166	0.02%
MuDR	2	819	0.00%
**DCS-specific***	9,113	2,999,343	9.14%
**Total interspersed repeats**		3,722,973	11.34%
Small RNA	0	0	0.00%
Satellites	3	432	0.00%
Simple repeats	4,753	300,081	0.91%
Low complexity	790	25,147	0.08%
**Total repeated sequences**		4,048,633	12.52%

* Repetitive sequences found only in *D. coniospora*.

Table S4. Length distribution of the repetitive elements in the *D. coniospora* genome

Repeats	Repeat class/family	Number of repeats	Total length (bp)	Percent of genome (%)
> 5 kb	DCS-specific*	89	522,342	1.59
2 - 5 kb	LINE	5	13,944	0.04
	LTR	2	4,727	0.01
	rRNA	2	6,255	0.02
	DCS-specific*	341	1,065,309	3.25
**Sum**		**350**	**1,090,235**	**3.32**
1 - 2 kb	LINE	14	17,851	0.05
	LTR	9	12,463	0.04
	rRNA	1	1,795	0.01
	Simple repeat	1	1,382	0.00
	DCS-specific*	423	605,046	1.84
**Sum**		**448**	**638,537**	**1.95**
500 - 1000 bp	DNA	7	4,243	0.01
	LINE	45	30,072	0.09
	LTR	9	7,373	0.02
	rRNA	1	517	0.00
	Simple repeat	10	6,377	0.02
	DCS-specific*	966	526,041	1.60
**Sum**		**1,038**	**574,623**	**1.75**
200 - 500 bp	DNA	67	20,056	0.06
	LINE	113	34,524	0.11
	Low complexity	8	2,157	0.01
	LTR	69	18,997	0.06
	RC/Helitron	1	304	0.00
	Satellite	7	1,975	0.01
	Simple repeat	118	30,462	0.09
	DCS-specific*	1,580	475,652	1.45
**Sum**		**1,963**	**584,127**	**1.78**
**Total**		**3,888**	**3,409,864**	**10.40**

* Repetitive sequences found only in *D. coniospora*.

Table S5. ***D. coniospora*** **genes orthologous to those that are involved in the asexual sporulation of** ***A. nidulans*** **and** ***Fusarium spp*****.**

See attached Excel file.

Table S6. Distribution of differentially expressed genes in KOG categories amongst *in vitro* growth stages

Type	Class	Differentially expressed	Total #	%
**INFORMATION STORAGE AND PROCESSING**		**163**	**927**	**17.6**
	Translation, ribosomal structure and biogenesis	79	272	29.0
	RNA processing and modification	16	196	8.2
	Transcription	34	218	15.6
	Replication, recombination and repair	19	161	11.8
	Chromatin structure and dynamics	15	80	18.8
**CELLULAR PROCESSES AND SIGNALING**		**204**	**1,327**	**15.4**
	Cell cycle control, cell division, chromosome partitioning	16	152	10.5
	Nuclear structure	1	24	4.2
	Defense mechanisms	7	21	33.3
	Signal transduction mechanisms	56	284	19.7
	Cell wall/membrane/envelope biogenesis	8	52	15.4
	Cell motility	1	3	33.3
	Cytoskeleton	15	96	15.6
	Extracellular structures	2	3	66.7
	Intracellular trafficking, secretion, and vesicular transport	18	258	7.0
	Posttranslational modification, protein turnover, chaperones	80	434	18.4
**METABOLISM**		**328**	**1,187**	**27.6**
	Energy production and conversion	58	200	29.0
	Carbohydrate transport and metabolism	47	169	27.8
	Amino acid transport and metabolism	63	220	28.6
	Nucleotide transport and metabolism	16	70	22.9
	Coenzyme transport and metabolism	15	74	20.3
	Lipid transport and metabolism	61	206	29.6
	Inorganic ion transport and metabolism	23	110	20.9
	Secondary metabolites biosynthesis, transport and catabolism	45	138	32.6
**POORLY CHARACTERIZED**		**167**	**862**	**19.4**
	General function prediction only	136	600	22.7
	Function unknown	31	262	11.8

Differential expression was defined as larger than four-fold change in expression between at least two of the three *in vitro* growth stages selected (mycelia, early conidiogenesis, and conidia).

Table S7. Comparative analysis of the predicted proteomes of fungi with various lifestyles

See attached Excel file.

Table S8. Functional classification of *D. coniospora* and *M. robertsii* proteins using KEGG Metabolic Pathway annotations

KEGG Metabolic Pathway	*D. coniospora*		*M. robertsii*	
	Number of proteins	% of proteome	Number of proteins	% of proteome
1.0 Global and overview maps	652	7.52	858	8.11
1.1 Carbohydrate metabolism	191	2.20	256	2.42
1.2 Energy metabolism	132	1.52	148	1.40
1.3 Lipid metabolism	107	1.23	152	1.44
1.4 Nucleotide metabolism	93	1.07	107	1.01
1.5 Amino acid metabolism	179	2.06	255	2.41
1.6 Metabolism of other amino acids	57	0.66	83	0.78
1.7 Glycan biosynthesis and metabolism	67	0.77	76	0.72
1.8 Metabolism of cofactors and vitamins	94	1.08	122	1.15
1.9 Metabolism of terpenoids and polyketides	34	0.39	49	0.46
1.10 Biosynthesis of other secondary metabolites	20	0.23	39	0.37
1.11 Xenobiotics biodegradation and metabolism	46	0.53	108	1.02
1.12 Chemical structure transformation maps	0	0.00	0	0.00

Table S9. Characteristics of fungal genomes used in this study

Species	NCBI ID	Genome Size (Mb)	Number of genes	GC content (%)	Repeat content (%)	Reference
***Drechmeria coniospora* ARSEF 6962**		32.8	8,281	55.0	12.5	This work
Arthrobotrys oligospora ATCC 24927	10671	40.0	11,479	44.5	0.5	[13]
Beauveria bassiana ARSEF 2860	910	33.7	10,364	51.5	2.0	[14]
Claviceps purpurea 20.1	11202	32.1	8,979	51.6	4.7	[15]
Cordyceps militaris CM01	10687	32.3	9,651	51.4	3.0	[16]
Cryptococcus neoformans var. neoformans JEC21	61	18.9	6,594	48.5	5.0	[17]
Epichloe glyceriae E277	6775	49.3	13,509	45.0	37.6	
Fusarium oxysporum f. sp. lycopersici strain 4287	707	59.9	17,735	48.5	28.1	[18]
Hirsutella minnesotensis 3608	11566	51.4	12,702	52.1	35.99	[10]
Hirsutella thompsonii MTCC6686	16933	36.4	9,798	52.6	NA	
Magnaporthe grisea W97-11	181181	37.9	11,109	51.6	9.7	[19]
Metarhizium acridum CQMa 102	2443	38.0	9,849	49.9	4.42	[20]
Metarhizium robertsii ARSEF 23	2190	39.2	10,583	51.5	2.12	[20]
Monacrosporium haptotylum CBS 200.50	17457	40.4	10,959	45.3	0.05	[21]
Nectria haematococca mpVI 77-13-4	537	54.43	15,707	50.8	5.1	[22]
Neotyphodium gansuense E7080	6832	39.6	7,306	44.4	36.1	

Neotyphodium gansuense var. inebrians E818	174039	29.7	7,291	47.0	13.7	
Neurospora crassa OR74A	19	39.2	10,813	49.9	3.2	
Ophiocordyceps sinensis CO18	11415	78.5	6,972	46.2	37.98	[23]
Pochonia chlamydosporia 123	11596	41.2	12,122	49.9	0.5	[24]
Podospora anserina S mat+	10832	35.5	10,548	52.2	5.0	[25]
Saccharomyces cerevisiae S288c	15	11.6	5,906	38.2	NA	[26]
Tolypocladium inflatum NRRL8044	11753	30.2	9,621	58	1.2	[11]
Trichoderma reesei QM6a	323	33.4	9,129	52.8	NA	[27]

Model / well-studied non-pathogen: *Saccharomyces cerevisiae, Neurospora crassa,* and *Podospora anserina*

Mycoparasite / saprophyte: *Trichoderma reesei*

Insect pathogen: *Hirsutella thompsonii, Tolypocladium inflatum, Ophiocordyceps sinensis, Beauveria bassiana, Cordyceps militaris, Metarhizium acridum,* and *Metarhizium robertsii*

Plant pathogen: *Claviceps purpurea, Epichloe glyceriae, Fusarium oxysporum, Nectria haematococca,* and *Magnaporthe grisea*

Plant symbiont: *Neotyphodium gansuense* and *Neotyphodium gansuense var. inebrians*

Nematode-trapping fungus: *Arthrobotrys oligospora* and *Monacrosporium haptotylum*

Nematode egg/cyst parasite: *Pochonia chlamydosporia*

NA, data not available.

Table S10. Comparison of the numbers of genes encoding plant biomass degradation enzymes in *D. coniospora* with those of selected plant or insect pathogenic fungi

Plant biomass material	Protein family	Description	DCS	FGR	MOR	MRO
Hemicellulose	GH10	Glycoside hydrolase family 10	1	10	6	0
Hemicellulose and pectin	GH43	Glycoside hydrolase family 43	2	34	18	1
Pectin	PL1	Pectin lyase family 1	1	18	2	0
	PL3	Pectin lyase family 3	0	14	1	0
	PL4	Pectin lyase family 4	3	6	1	0
Crystalline cellulose	CBM1	Carbohydrate-binding module family 1	5	6	13	4
	GH6	Glycoside hydrolase family 6	0	2	3	0
	GH7	Glycoside hydrolase family 7	0	4	5	0
Lignin	AA3	Cellobiose dehydrogenases; GMC oxidoreductases; alcohol oxidases; pyranose oxidases	18	34	15	17

DCS: *D. coniospora;* FGR: *F. graminearum*; MOR: *Ma. oryzae*; MRO: *M. robertsii*

Table S11. The category of selected genes present in five facultative endoparasites but missing from the *D. coniospora* genome

Gene category	KEGG#	EC#	Definition
**Peroxisome**			
PEX12, PAF3	K13345	-	Peroxin-12
PXMP4, PMP24	K13350	-	Peroxisomal membrane protein 4
SCP2, SCPX	K08764	EC:2.3.1.176	Sterol carrier protein 2
SOD1	K04565	EC:1.15.1.1	Superoxide dismutase, Cu-Zn family
katE, CAT, *catB*, *srpA*	K03781	EC:1.11.1.6	Catalase
PEX2, PXMP3	K06664	-	Peroxin-2
**Purine metabolism**			
uraH, *pucM*, *hiuH*	K07127	EC:3.5.2.17	5-hydroxyisourate hydrolase
rpoA	K03018	EC:2.7.7.6	DNA-directed RNA polymerase subunit alpha
	K03787	EC:3.1.3.5	5'-nucleotidase
	K00601	EC:2.1.2.2	Phosphoribosylglycinamide formyltransferase
URE	K01427	EC:3.5.1.5	Urease
purD	K11788	EC:6.3.4.13 6.3.3.1	Phosphoribosylamine - glycine ligase
purM		EC:6.3.4.13 6.3.3.1	Phosphoribosyl formylglycinamidine cyclo-ligase
**Propanoate metabolism**			
paaF, *echA*	K01692	EC:4.2.1.17	Enoyl-CoA hydratase
LDH, *ldh*	K00016	EC:1.1.1.27	L-lactate dehydrogenase
ackA	K00925	EC:2.7.2.1	Acetate kinase
	K01505	EC:3.5.99.7	1-aminocyclopropane-1-carboxylate deaminase
prpE	K01908	EC:6.2.1.17	Propionyl-CoA synthetase
**Fatty acid degradation**			
paaF, *echA*	K01692	EC:4.2.1.17	Enoyl-CoA hydratase
ADH1_7	K13953	EC:1.1.1.1	Alcohol dehydrogenase 1/7
	K14338	EC:1.14.14.1	Unspecific monooxygenase
**Cysteine and methionine metabolism**			
ldh	K00016	EC:1.1.1.27	L-lactate dehydrogenase
mmuM	K00547	EC:2.1.1.10	Homocysteine S-methyltransferase
metX	K00641	EC:2.3.1.31	Homoserine O-acetyltransferase
metB	K01739	EC:2.5.1.48	Cystathionine gamma-synthase

**Tyrosine metabolism**			
ADH1_7	K13953	EC:1.1.1.1	Alcohol dehydrogenase 1/7
HGD, *hmgA*	K00451	EC:1.13.11.5	Homogentisate 1,2-dioxygenase
TYR	K00505	EC:1.14.18.1	Tyrosinase
**Tryptophan metabolism**			
paaF, *echA*	K01692	EC:4.2.1.17	Enoyl-CoA hydratase
	K14338	EC:1.14.14.1	Unspecific monooxygenase
katE, CAT, *catB*, *srpA*	K03781	EC:1.11.1.6	Catalase
**Valine, leucine and isoleucine degradation**			
paaF, *echA*	K01692	EC:4.2.1.17	Enoyl-CoA hydratase
mmsB	K00020	EC:1.1.1.31	3-hydroxyisobutyrate dehydrogenase
**Oxidative phosphorylation**			
ATPF0A, *atpB*	K02142	EC:3.6.3.14	F-type H+-transporting ATPase, subunit A
ccoN	K02267	EC:1.9.3.1	Cytochrome c oxidase, cbb3-type, subunit I
	K03938	EC:1.6.5.3 1.6.99.3	NADH dehydrogenase
	K03955	EC:1.6.5.3 1.6.99.3	NADH dehydrogenase
**Sulfur metabolism**			
cysJ	K00380	EC:1.8.1.2	Sulfite reductase (NADPH) flavoprotein alpha-component
SUOX	K00387	EC:1.8.3.1	Sulfite oxidase
metB	K01739	EC:2.5.1.48	Cystathionine gamma-synthase
**Nitrogen metabolism**			
nirB	K00362	EC:1.7.1.4	Nitrite reductase (NADH) large subunit
NRT, *narK*, *nrtP*, *nasA*	K02575	-	MFS transporter, NNP family, nitrate/nitrite transporter
NR	K10534	EC:1.7.1.3	Nitrate reductase (NAD(P)H)
**Glycerolipid metabolism**			
AKR1A1, *adh*	K00002	EC:1.1.1.2	Alcohol dehydrogenase (NADP+)
DAK1, DAK2	K00863	EC:2.7.1.29	Dihydroxyacetone kinase
**Metabolism of xenobiotics by cytochrome P450 (2)**			
ADH1_7	K13953	EC:1.1.1.1	Alcohol dehydrogenase 1/7
GST	K00799	EC:2.5.1.18	Glutathione S-transferase
GST	K00799	EC:2.5.1.18	Glutathione S-transferase

Three insect pathogens (*Metarhizium robertsii*, *Metarhizium acridum*, and *Beauveria bassiana*), the nematode-trapping fungus *Arthrobotrys oligospora*, and the plant pathogen *Fusarium oxysporum* were selected for comparison. Genes highlighted in *grey* encode proteins that participate in more than one KEGG metabolic pathway.

Table S12. Number of predicted *D. coniospora* proteins featuring various PFAM domains (number > 3)

See attached Excel file.

Table S13. Stress response proteins encoded in the *D. coniospora* genome

See attached Excel file.

Table S14. Functional domain annotation of *D. coniospora* SSPs

**Domain description**	**Occurrences in SSPs**	**Occurrences in *D. coniospora***	**P- value***
PF12296.3, Hydrophobic surface binding protein A	6	21	5.3 e-05
PF06766.6, Fungal hydrophobin	5	5	0.00
PF09132.5, BmKX	4	4	0.00
PF07846.6, Metallothionein family	4	4	0.00
PF05572.8, Pregnancy-associated plasma protein-A	3	8	9.3 e-03
PF05730.6, CFEM domain	3	17	4.0 e-04
PF09056.6, Prokaryotic phospholipase A2	2	2	0.00
PF14273.1, Domain of unknown function (DUF4360)	2	2	0.00
PF00379.18, Insect cuticle protein	2	2	1.3 e-04
PF03717.10, Penicillin-binding Protein dimerisation domain	2	2	0.00
PF01470.12, Pyroglutamyl peptidase	2	3	6.1 e-03
PF01828.12, Peptidase A4 family	2	3	1.3 e-04
PF07876.7, Stress responsive A/B Barrel Domain	2	4	2.1 e-02
PF13405.1, EF-hand domain	2	6	2.1 e-02
PF02199.10, Saposin A-type domain	2	8	5.1 e-04

85 putative SSPs were annotated based on the presence of one or more Pfam domains. Only those Pfam domains that occur more than once are shown.

* P value is determined by the hypergeometric test, considering only the proteins with an identified Pfam domain.

Table S15. ***D. coniospora*** **protein families potentially involved in pathogenicity**

See attached Excel file.

Table S16. NRPS, PKS and NRPS-PKS genes and the corresponding gene clusters in *D. coniospora*

Table S16A. *D. coniospora* NRPS, PKS and NRPS-PKS genes and their transcript abundances in different life stages

Name	Gene ID	Length (bp)	Transcript abundances (FPKM)			
			Mycelia	Conidiogenesis	Conidia	Infection
DcPKS1	DCS_04392	8,746	84.1	3.3	1.1	0.5
DcPKS2	DCS_04399	1,523	50.8	2.6	0.1	0.7
DcPKS3	DCS_02243	5,537	0.5	0.1	0.1	0.1
DcPKS4	DCS_02241	7,618	1.9	0.5	0.8	4.6
DcPKS5	DCS_02240	1,191	0	0.1	0.1	0.2
DcPKS6	DCS_01787	7,446	0.7	0.1	0.1	0.0
DcPKS7	DCS_05930	7,900	12.9	7.6	7.7	8.1
DcPKS8	DCS_02466	2842	3.9	13.2	1.9	0.7
DcNRPS1	DCS_04402	36,563	8.4	0.2	0.1	0.1
DcNRPS2	DCS_02860	7,780	5.6	18.1	14.0	255.1
DcNRPS3	DCS_02863	3,272	0.6	2.7	12.5	6.7
DcNRPS4	DCS_07701	7,700	24.7	24.5	176.1	36.0
DcNRPS5	DCS_03520	9,821	182.4	6.6	12.5	5.9
DcNRPS6	DCS_02295	14,656	2.8	0.9	0.2	0.2
DcNRPS7	DCS_00047	18,731	0.1	0.2	0.2	0.2
DcNRPS8	DCS_01405	10,148	4.8	2.0	5.3	4.6
DcNRPS9	DCS_05475	5,992	24.1	16.6	15.6	21.5
DcNRPS10	DCS_06786	9,267	6.2	7.5	21.7	7.7
DcNRPS11	DCS_03476	4,645	17.9	25.7	23.5	28.3
DcNRPS-PKS1	DCS_04285	5,546	2.0	6.0	12.2	31.5
DcNRPS-PKS2	DCS_06362	11,519	13.9	0.1	0.1	5.0
DcNRPS-PKS3	DCS_03296	1916	18.2	330.2	879.4	211.6

Table S16B. *D. coniospora* secondary metabolite biosynthetic loci and the transcript abundances of the genes (in FPKM) in different life stages

See attached Excel file.

Table S17. Cytochrome P450s (CYPs) of *D. coniospora* and their expression in different life stages

CYP clade	Gene ID	Transcript abundances (FPKM)			
		Mycelia	Conidiogenesis	Conidia	Infection
50	DCS_01702	0.17	0.20	0.04	0.00
	DCS_04293	77.90	180.79	105.72	424.48
51	DCS_04389	421.80	10.76	1.75	0.77
	DCS_07591	35.45	41.75	61.86	86.19
52	DCS_02865	6.09	27.01	39.57	26.09
	DCS_03434	15.87	152.07	116.55	146.67
	DCS_06205	43.73	33.97	10.26	11.97
53	DCS_01685	9.92	35.85	64.31	48.79
	DCS_01815	10.16	13.43	13.61	10.67
	DCS_03406	68.60	10.87	24.46	13.18
	DCS_04355	5.76	1.88	1.44	0.40
	DCS_05025	43.14	45.25	28.71	18.98
	DCS_03361	24.11	30.07	26.28	16.52
	DCS_07818	53.42	20.39	10.72	10.31
54	DCS_06091	15.26	14.18	14.73	25.73
	DCS_07966	31.04	76.00	77.82	32.26
	DCS_01008	10.53	9.72	16.84	14.76
	DCS_01367	62.59	26.93	22.86	13.19
	DCS_00046	0.12	0.07	0.23	0.38
	DCS_04393	248.01	7.60	0.60	0.94
	DCS_06802	35.74	20.80	26.79	22.28
55	DCS_00079	1105.22	622.22	68.48	13.65
56	DCS_02392	40.44	1.81	2.86	0.94
	DCS_04106	9.50	3.22	1.00	1.81
57	DCS_02704	5.58	1.87	5.12	1.38
	DCS_04260	21.92	7.27	6.67	7.74
60	DCS_01922	232.65	39.47	22.40	17.20
61	DCS_03086	0.16	0.15	0.05	0.40

	DCS_00834	78.59	104.75	139.75	148.84
	DCS_07539	65.24	7.19	4.33	1.82
62	DCS_04885	45.68	29.31	45.79	41.38
64	DCS_04618	7.57	32.28	46.74	109.87
65	DCS_04387	51.97	6.51	2.28	1.34
69	DCS_01698	8.92	46.13	16.66	2.05

Transcript abundances are shown in FPKM.

Table S18. *D. coniospora* orthologs of proteins involved in iron metabolism in other fungi

See attached Excel file.

Table S19. Accurate MS/MS data for drechmerin congeners (see Fig S14)

Drechmerin congener	Fragment	Calculated	Observed	Δ ppm
Drechmerin A	$[M+H]^+$	1190.8492	1190.8485	-0.6
	B9	1031.7120	1031.7107	-1.3
	B8	946.6593	946.6584	-0.9
	B7	861.6065	861.6063	-0.2
	B6	748.5224	748.5226	0.2
	B5	565.3601	565.3602	0.1
	B4	480.3074	480.3071	-0.5
	B3	395.2546	395.2542	-1.0
	B2	182.1181	182.1181	0.0
	A2	154.1232	154.1221	-7.1*
	Y9	1009.7389	1009.7364	-2.5
	Y8	796.6024	796.6021	-0.4
	Y7	711.5497	711.5508	1.6
	Y6	626.4969	626.4965	-0.6
	Y5	443.3346	443.3339	-1.5
	Y4	330.2505	330.2504	-0.3
	Y3	245.1978	245.1984	2.6
	Y2	160.1450	160.1444	-3.7
Dehydro-drechmerin A	[M+H]+	1172.8386	1172.8391	0.4
	B9	1013.7015	1013.7010	-0.5
	B8	928.6487	928.6487	0.0
	B7	843.5959	843.5968	1.0
	B6	730.5119	730.5129	1.4
	B5	547.3496	547.3502	1.2
	B4	462.2968	462.2969	0.2
	B3	377.2440	377.2441	0.2
	B2	182.1181	182.1183	1.1
	A2	154.1232	154.1225	-4.5
	Y9	991.7283	991.7288	0.5
	Y8	796.6024	796.6014	-1.3

	Y7	711.5497	711.5501	0.6
	Y6	626.4969	626.4974	0.8
	Y5	443.3346	443.3342	-0.9
	Y4	330.2505	330.2509	1.2
	Y3	245.1978	245.1987	3.9
	Y2	160.1450	160.1443	-4.3
Drechmerin B	$[M+H]^+$	1176.8335	1176.8330	-0.5
	B9	1017.6964	1017.6951	-1.3
	B8	932.6436	932.6428	-0.9
	B7	847.5909	847.5906	-0.3
	B6	734.5068	734.5068	0.0
	B5	551.3445	551.3444	-0.1
	B4	466.2917	466.2914	-0.7
	B3	395.2546	395.2543	-0.8
	B2	182.1181	182.1181	0.0
	A2	154.1232	154.1223	-5.8*
	Y9	995.7233	995.7271	3.9
	Y8	782.5868	782.5899	4.0
	Y7	711.5497	711.5499	0.3
	Y6	626.4969	626.4981	1.9
	Y5	443.3346	443.3350	0.9
	Y4	330.2505	330.2505	0.0
	Y3	245.1978	245.1981	1.4
	Y2	160.1450	160.1440	-6.2*
Dehydro-drechmerin B	[M+H]+	1158.8230	1158.8230	0.0
	B9	999.6858	999.6859	0.1
	B8	914.6331	914.6330	-0.1
	B7	829.5803	829.5811	1.0
	B6	716.4962	716.4970	1.1
	B5	533.3339	533.3343	0.7
	B4	448.2811	448.2810	-0.3
	B3	377.2440	377.2438	-0.6

B2	182.1181	182.1181	0.0
A2	154.1232	154.1225	-4.5
Y9	977.7127	977.7172	4.6
Y8	782.5868	782.5884	2.1
Y7	711.5497	711.5487	-1.3
Y6	626.4969	626.4975	1.0
Y5	443.3346	443.3345	-0.2
Y4	330.2505	330.2507	0.6
Y3	245.1978	245.1984	2.6
Y2	160.1450	160.1443	-4.3

*, >5 ppm differences between observed and calculated masses are due to error expected for masses outside the calibration range (m/z 170-1900). Fragment nomenclature follows Roepstorff and Fohlman[9].

References

1 Lai, Y. L. *et al.* Comparative genomics and transcriptomics analyses reveal divergent lifestyle features of nematode endoparasitic fungus *Hirsutella minnesotensis*. *Genome Biol. Evol.* **6**, 3077-3093, doi:10.1093/gbe/evu241 (2014).

2 Bushley, K. E. *et al.* The genome of *Tolypocladium inflatum*: Evolution, organization, and expression of the cyclosporin biosynthetic gene cluster. *PLoS Genet* **9**, doi:10.1371/journal.pgen.1003496 (2013).

3 Ward, E. *et al.* The Pochonia chlamydosporia Serine Protease Gene vcp1 Is Subject to Regulation by Carbon, Nitrogen and pH: Implications for Nematode Biocontrol. *Plos One* **7**, doi:10.1371/journal.pone.0035657 (2012).

4 Yang, J. K. *et al.* Genomic and proteomic analyses of the fungus *Arthrobotrys oligospora* provide insights into nematode-trap formation. *Plos Pathog* **7**, e1002179, doi:10.1371/journal.ppat.1002179 (2011).

5 Gao, Q. A. *et al.* Genome sequencing and comparative transcriptomics of the model entomopathogenic fungi *Metarhizium anisopliae* and *M. acridum*. *PLoS Genet.* **7**, 18, doi:e100126410.1371/journal.pgen.1001264 (2011).

6 Xiao, G. *et al.* Genomic perspectives on the evolution of fungal entomopathogenicity in *Beauveria bassiana*. **2**, 483, doi:10.1038/srep00483 (2012).

7 Schardl, C. L. *et al.* Plant-symbiotic fungi as chemical engineers: multi-genome analysis of the clavicipitaceae reveals dynamics of alkaloid loci. *PLoS Genet.* **9**, e1003323, doi:10.1371/journal.pgen.1003323 (2013).

8 Zheng, P. *et al.* Genome sequence of the insect pathogenic fungus Cordyceps militaris, a valued traditional Chinese medicine. *Genome Biol.* **12**, R116, doi:10.1186/gb-2011-12-11-r116 (2011).

9 Loftus, B. J. *et al.* The genome of the basidiomycetous yeast and human pathogen Cryptococcus neoformans. *Science (New York, N.Y.)* **307**, 1321-1324, doi:10.1126/science.1103773 (2005).

10 Ma, L. J. *et al.* Comparative genomics reveals mobile pathogenicity chromosomes in Fusarium. *Nature* **464**, 367-373, doi:10.1038/nature08850 (2010).

11 Dean, R. A. *et al.* The genome sequence of the rice blast fungus Magnaporthe grisea. **434**, 980-986, doi:10.1038/nature03449 (2005).

12 Pattemore, J. A. *et al.* The genome sequence of the biocontrol fungus Metarhizium anisopliae and comparative genomics of Metarhizium species. **15**, 660, doi:10.1186/1471-2164-15-660 (2014).

13 Meerupati, T. *et al.* Genomic mechanisms accounting for the adaptation to parasitism in nematode-trapping fungi. *PLoS Genet.* **9**, 20, doi:10.1371/journal.pgen.1003909 (2013).

14 Coleman, J. J. *et al.* The genome of Nectria haematococca: contribution of supernumerary chromosomes to gene expansion. *PLoS Genet.* **5**, e1000618, doi:10.1371/journal.pgen.1000618 (2009).

15 Hu, X. *et al.* Genome survey uncovers the secrets of sex and lifestyle in caterpillar fungus. *Chin. Sci. Bull.* **58**, 2846-2854, doi:10.1007/s11434-013-5929-5 (2013).

16 Larriba, E. *et al.* Sequencing and functional analysis of the genome of a nematode egg-parasitic fungus, *Pochonia chlamydosporia*. *Fungal genetics and biology : FG & B* **65**, 69-80, doi:http://dx.doi.org/10.1016/j.fgb.2014.02.002 (2014).

17 Clutterbuck, A. J. Genomic evidence of repeat-induced point mutation (RIP) in filamentous ascomycetes. *Fungal Genet. Biol.* **48**, 306-326, doi:10.1016/j.fgb.2010.09.002 (2011).

18 Treu, L. *et al.* The impact of genomic variability on gene expression in environmental Saccharomyces cerevisiae strains. *Environmental microbiology* **16**, 1378-1397, doi:10.1111/1462-2920.12327 (2014).

19 Martinez, D. *et al.* Genome sequencing and analysis of the biomass-degrading fungus Trichoderma reesei (syn. Hypocrea jecorina). **26**, 553-560, doi:10.1038/nbt1403 (2008).

20 Roepstorff, P. & Fohlman, J. Proposal for a common nomenclature for sequence ions in mass spectra of peptides. *Biomedical mass spectrometry* **11**, 601, doi:10.1002/bms.1200111109 (1984).

Supplementary Results

Insights into adaptations to a near-obligate nematode endoparasitic lifestyle from the finished genome of *Drechmeria coniospora*

Liwen Zhang, Zhengfu Zhou, Qiannan Guo, Like Fokkens, Márton Miskei, István Pócsi, Wei Zhang, Ming Chen, Lei Wang, Yamin Sun, Bruno G. G. Donzelli, Donna M. Gibson, David R. Nelson, Jian-Guang Luo, Martijn Rep, Hang Liu, Shengnan Yang, Jing Wang, Stuart B. Krasnoff, Yuquan Xu*, István Molnár*, and Min Lin*

Contents

Repetitive elements and transposons 61

Repeat-induced point mutations (RIPs) 62

Cryptic sexual cycle 63

Asexual sporulation 64

Adaptation to a reduced lifestyle repertoire leads to proteome contraction 65

Transporters 66

Pfam domain analysis 67

Regulatory circuits and stress response 67

Pathogenicity islands composed of small secreted proteins (SSPs) and pathogen-host interaction proteins 68

Hydrolytic enzymes 69

Iron acquisition 71

Secondary metabolism 73

Drechmerins: structure, biosynthesis and bioactivities 74

References 79

Repetitive elements and transposons

Duplication events associated with recombination, mutation and consequent diversification are major contributors to the evolution of fungal genomes, and facilitate their adaptation to environmental constraints[28]. The complete genome assembly of *D. coniospora* features a repeat sequence content of 12.5% (4.11 Mb). Although this number is higher than the proportion of repeats published for most other ascomycete genome assemblies (0.1–6.0%) except for that of *Hirsutella minnesotensis* (36%) (Table S2)[29], this discrepancy is probably caused by our successful coverage of the repeat-rich centromere and telomere regions.

Amongst the repeated elements, 731 transposons comprise 2.2% of the genome, with Type I retrotransposons dominating over Type II DNA transposons (618 vs. 113, respectively) (Table S3). The retrotransposons *gypsy* (253), *copia* (211) and LINE (98) are the most abundant types, followed by the DNA transposon *cacta* (75). Retrotransposons are enriched in centromere regions (average density: 162/Mb in centromeres vs. 11/Mb in the rest of the genome), while DNA transposons appear scattered along the chromosomes (Fig. 2). Simple repeats contribute another 0.3 Mb (0.9%) to the genome.

Strikingly, the large majority of repetitive sequences (almost 3 Mb, 74% of the total repeat content) are specific to *D. coniospora.* Long *D. coniospora*-specific repeats (>2 kb, 4.8% of the genome, Table S4) fall into 10 different families. These repeats concentrate in the centromeres and the terminal regions of the chromosomes, with centromeres on different chromosomes sharing the same set of homologous families (Figs. 2 and S2). Mid-sized *D. coniospora*-specific repeats (0.5-2 kb) constitute another 3.4% of the genome, with the majority of these elements also accumulating in the centromere and in the terminal regions.

Small *D. coniospora*-specific repeats (0.2-0.5 kb) occupy 1.5% of the genome and are scattered along the three chromosomes. Thus, the distributions of repeats along the *D. coniospora* chromosomes is non-homogenous, with centromeric and terminal regions harboring most of the species-specific repeats and retrotransposons, but contain relatively few coding sequences (Fig. 2).

Repeat-induced point mutations (RIPs)

The completed genome of *D. coniospora* shows clear evidence for an active repeat-induced point mutation (RIP) system. RIP is a sequence similarity-dependent genome defense system that operates during sexual reproduction; it introduces multiple C-to-T transitions in duplicated DNA sequences, promoting sequence diversification[21,25,30,31]. RIP also increases the frequency of stop codons, and may consequently truncate open reading frames. Orthologs of the RIP-associated cytosine methyltransferase genes *rid* and *dim-2* are both present in the *D. coniospora* genome (DCS_01041 and DCS_08015, respectively), while absent in the closely related insect-pathogen *Tolypocladium inflatum* and nematode egg/cyst parasite *Pochonia chlamydosporia*. Mutation of *rid* inactivates RIP in *Neurospora crassa*[32], and RID is also involved in sexual development in *Aspergillus nidulans*[33]. DIM-2 was found to act on RIP-affected sequences, but its mutation has no effect on RIP in *N. crassa*[25]. The presence of the *rid* gene has been considered a hallmark of RIP competency in sequenced fungal genomes[2,34].

In *D. coniospora*, RIP activity may be important to limit the activity of transposons. Thus,

local maxima of the TpA/ApT index, indicating enrichment of RIP-related mutations, clearly overlap with retrotransposons along the three chromosomes (Fig. 2). The (CpA+TpG)/(ApC+GpT) index that estimates the depletion of RIP targets reveals a similar trend (results not shown). Clear signs of RIP were evident in the retrotransposons *gypsy*, *copia* and DDE, with CpA-to-TpA and CpT-to-TpT mutations dominating (Fig. S3). Multiple sequence comparisons among 11 transposase genes with nucleotide sequence identities larger than 90% showed that single nucleotide polymorphisms were nearly exclusively C vs. T or G vs. A, suggesting recent RIP events (Fig. S4). In contrast, we found no evidence of RIP in the retrotransposon LINE and the DNA transposon *cacta*.

Cryptic sexual cycle

Sexual reproduction is an important way for fungi to exchange genetic material and is thus one of the drivers of their evolution. Ascomycete fungi may be heterothallic (outcrossing) or homothallic (self-crossing)[13]. Crossing requires two types of compatible mating-type (MAT) idiomorphs. Investigation of the *D. coniospora* genome revealed a *MAT1-1* idiomorph (DCS_00888) encoding a well-conserved *MAT1-1-1* ortholog (61% identity to GenBank: AGW27562.1, the *MAT1-1-1* of *T. inflatum*). The α-domain protein MAT1-1-1 is critical for *MAT1-1* identity and sexual development[35]. While DCS_00888 is upregulated during sporulation as compared to vegetative mycelia (FPKM values 3.2 *vs*. 0.3, respectively), the transcript abundance remains very low. Genes indicative of the *MAT1-2* idiomorph (including an HMG domain gene that is necessary to establish the *MAT1-2* identity[35]) were absent in the sequenced strain, suggesting that *D. coniospora* is heterothallic if a sexual

reproduction cycle exist. In addition, the *D. coniospora* genome also encodes a late sexual development protein (DCS_00280, 75% identity to GenBank: EFY96670.1 from *Metarhizium robertsii* ARSEF23[20]) that was significantly upregulated during sporulation, with the FPKM value increasing to 1618.2 as compared to 29.0 during the mycelial stage. The presence of a *MAT1-1* idiomorph and evidence of a recent RIP activity implicate that this organism may have a cryptic sexual state in nature and is likely to be heterothallic. This is in accordance with the closely related insect pathogens *T. inflatum*[11], *M. robertsii* and *M. acridum*[2] and *B. bassiana*[23], while in contrast to *O. sinensis*[23] which are homothallic.

Asexual sporulation

Copious production of asexual spores is crucial for the pathogenic cycle of *D. coniospora*. Considering the observed cell differentiation events that lead to the production of conidia[36], we expected that the *D. coniospora* genes necessary for the development of conidiferous pegs and those for the formation, maturation and release of conidia would be similar to those involved in the formation of conidiogeneous cells (phialide) and conidia by the fusaria[37,38], but quite different from those necessary for the complex phialide-bearing structures (foot cell, nonseptate stalk, vesicle, metulae) typically observed in the aspergilli[39,40]. Exhaustive searches for conidiogenesis-related genes in the genome of *D. coniospora* reaffirm this conclusion (Table S5). Just as in fusaria, no ortholog exists in *D. coniospora* for BrlA, a key regulator of vesicle development in *A. nidulans*[41]. Similarly, orthologs of the *F. graminearum* regulators AbaA (involved in differentiation from vegetative hyphae to conidia) and WetA (related to conidia maturation)[37] are clearly detectable in *D. coniospora*, but these

predicted proteins are only distantly related to AbaA and WetA of *A. nidulans*[41]. The conserved AbaA-WetA pathway is essential for conidiogenesis in both the aspergilli[41] and the fusaria[37].

Adaptation to a reduced lifestyle repertoire leads to proteome contraction

Although the gene density of *D. coniospora* at 253 genes/Mb is at the low end for related facultative insect and nematode pathogens[2,29] (Table S2), this density still indicates that inflation of noncoding DNA regions did not contribute significantly to the reduction of protein coding capacity. Generally, there are fewer proteins in each of the KEGG metabolic pathway categories in *D. coniospora* than in the insect pathogen *M. robertsii*[2] (Table S8). Proteins involved in carbohydrate, lipid and amino acid metabolism are especially depleted, suggesting a higher reliance of *D. coniospora* on host nutrients. Similar to the entomopathogenic members of Ophiocordycipitaceae[11,23] and Clavicipitaceae, *D. coniospora* features a much reduced repertoire of enzymes necessary for the degradation of plant biomass, reflecting the divergence from the plant pathogenic lifestyle (Table S10).

The limited viability of the fungus and its seriously compromised ability for morphogenesis and development under standard laboratory growth conditions may result from the loss of genes involved in (but not strictly required) for a saprophytic lifestyle. A systematic search for genes that are absent in the *D. coniospora* genome but conserved in five facultative parasitic fungi from diverse life strategy groups returned 501 genes. Many of these genes are involved in lipid, nucleotide and amino acid metabolic pathways (Table S11, Fig. S7),

reflecting the heightened reliance of the endoparasite on the utilization of nutrients from host tissues. Similarly, genes for enzymes involved in inorganic nitrogen assimilation during saprophytic growth (nitric oxide reductase and nitrate reductase) are missing from the *D. coniospora* genome. However, an ortholog (DCS_05106) of the nitrate-responsive regulatory protein NirA from *B. bassiana* (XP_008603679.1) is still present. This putative *nirA* is highly expressed during nematode infection (FPKM: 6,965, compared to average FPKM: 89.59 ± 17.02), and is also upregulated as compared to the spore stage (more than 8-fold), indicating an active response to nitrogen availability in the host.

Transporters

In a stark contrast to the contraction of many protein families, the number of transporters encoded in the *D. coniospora* genome is remarkably high (Fig. 4A). Many transporter clades, including the primary active transporters (310), the electrochemical potential-driven transporters (292) and the channels/pores (89) have undergone significant expansion, and an additional 117 incompletely characterized transport systems are also encoded in the genome of this nematode endoparasite. Amongst the primary active transporters, the ATP-binding cassette (ABC) family pumps are often involved in the defense of pathogens from host-produced toxins[2]. ABC transporters also expanded in *D. coniospora* genome (44), and all these pumps are orthologous to exporters catalogued in the pathogen-host interaction (PHI) database[6]. The expansion of these transporters may reflect the heightened dependence of *D. coniospora* on host nutrients, and the acute need of this pathogen to protect itself from host-derived defense substances.

Pfam domain analysis

Amongst the 2,924 *D. coniospora* proteins that could be annotated with 1,158 different Pfam domains, proteins with serine/threonine protein kinase-like domains (104), fungal transcription factor domains (60), RNA recognition motif domains (48), and Zn_2C_6-type fungal transcription factor domains (42) dominate, followed by proteins featuring various transporter family domains and hydrolytic enzyme domains (peptidase, glycoside hydrolase, etc.) (Table S12). The high frequency of putative regulatory network elements, catabolic enzymes, and transporters amongst the proteins with recognizable conserved motifs suggests that *D. coniospora* metabolism needs to be carefully fine-tuned to the acquisition of host nutrients.

Regulatory circuits and stress response

Lifestyle and host range switches may rely more on the variation of regulatory circuits while maintaining the overall framework of metabolism. Comparison of protein families among fungi from different lifestyles (Fig. 4A) also supports this conclusion. Thus, the numbers of transcription factors, protein kinases, and GPCR-like proteins involved in signal transduction are strongly contracted in *D. coniospora*, suggesting that this fungus might only respond to a reduced variety of extracellular signals.

Similarity searches against a curated collection of verified stress response elements[42] identified 916 putative stress response proteins (Table S13). 210 of these are shared by

insect and plant pathogens, suggesting that these may be involved in coping with the host environment in pathogens. However, other elements of stress sensing (*e.g.* the oxidative stress response histidine kinase TcsA) and transcriptional regulators downstream of stress signaling pathways (*e.g.* MsnA or RlmA in oxidative and cell wall integrity stress signaling) showed less clear similarities with *D. coniospora* proteins. Even more remarkably, 466 database entries (21.8 % of the database) were missing orthologs in the endoparasitoid. A large portion (103, 22.1%) of these missing stress proteins are transcription factors, suggesting that in order to adapt to an ecological niche, it is the transcription factors that need to change first and foremost.

The chromatin remodeling protein LaeA of *A. nidulans* is central to the regulation of sexual sporulation and secondary metabolite production as part of the trimeric VelB-VeA-LaeA complex[43,44]. In fusaria, LaeA orthologs suppress sexual sporulation[45], but activate secondary metabolite production and virulence[45-48]. The LaeA-type global regulators of aspergilli, fusaria and *D. coniospora* show surprisingly low (<40%) identities. Considering that fusaria are phylogenetically more closely related to *D. coniospora* than the aspergilli, we propose that LaeA may also act as a negative regulator of sexual sporulation in this endoparasite (Fig. 5).

Pathogenicity islands composed of small secreted proteins (SSPs) and pathogen-host interaction proteins

Manipulation of the host by pathogens often involves the broadcasting of effector proteins that may be expressed exclusively during infection[49]. These are typically small, secreted,

cysteine-rich proteins that may be genus-, species-, or even isolate-specific[50-52]. SSPs are often involved in host-specific pathogenicity, and include avirulence determinants, toxins, and suppressors of host defense and signaling[51,52]. Compared to intracellular small proteins, the predicted *D. coniospora* SSPs tend to have more cysteines ($p < 2e-19$, Kolmogorov-Smirnov test), and the number of these cysteines tends to be even ($p < 4e-07$, hypergeometric test, Fig. S12), ensuring a more compact structure to facilitate secretion. Most predicted SSPs (79.7%) were transcribed (FPKM > 1) under at least one life stage, with the majority (~79.0%) changing their transcription levels at least 2-fold among different growth phases. Among the 84 predicted SSPs that were highly expressed (> mean $\pm$ 2 $\times$ standard deviation) during at least one growth period, 42 are recent inventions. In a word, *D. coniospora* SSPs are fast evolving; their expression is mostly growth stage-dependent; and some SSPs are induced by the presence of the nematode prey, suggesting a dynamic interaction with the host defense system.

Hydrolytic enzymes

Chitin is a structural component of fungal cell walls as well as arthropod cuticles and nematode egg shells[53], but is much less prevalent in the collagenous exoskeleton of nematodes[54]. As expected, the insect pathogen *M. robertsii* and the nematode egg/cyst parasitic fungus *P. chlamydosporia* feature the largest variety of chitinases, while *D. coniospora* and *Ar. oligospora* have less proteins of this enzyme family (Fig. 4A and C).

Acid phosphatase activity has been detected at the site of penetration during nematode

invasion by *D. coniospora*[55]. Two of the seven acid phosphatases encoded in the genome (DCS_04121 and DCS_06910) were upregulated in the spore stage and during nematode infection (Table S15B), identifying these enzymes as plausible candidates for further biological characterization. The role of metalloproteases in nematode penetration has been challenged[56]: correspondingly, none of the corresponding genes encoded in *D. coniospora* were overexpressed during infection, although one (DCS_08224) was preferentially expressed during mycelial growth and sporulation (Table S15B).

Some dehydrogenases show a high level of transcription during nematode infection and/or mycelial growth (Table S15B). The CYP superfamily as a whole is contracted in *D. coniospora* (Figs. 4A and S13)[57]. While *D. coniospora* retained representatives of various CYP clans, most of these clans have undergone a drastic contraction (Table S17). Nevertheless, 4 clans expanded to encompass 59% of the *D. coniospora* CYPome, with 9, 5, 3 and 3 paralogs each for the CYP53, CYP54, CYP52 and the CYP61 clans, respectively (Table S17). Members of the CYP52 clan are involved in the oxidation of alkanes and fatty acids, while the CYP53 clan includes BbCYP53A26 from *B. bassiana* that contributes to the degradation of various hydrocarbons and lipids in the insect epicuticle[58]. Thus, the *D. coniospora* CYPome appears to be streamlined towards the decomposition of the nematode exoskeleton. Most of the CYPs showed moderate expression levels (< mean – 2 × standard deviation, Table S17). Four CYPs from clan 50, 52, 61 and 64 each were highly expressed during infection stage and upregulated compared to other stages, while four CYPs from clan 51, 54, 55 and 60 were highly expressed and upregulated during mycelial growth (Table S17).

Iron acquisition

Every organism needs to maintain iron homeostasis by managing iron acquisition and storage. Iron acquisition is especially problematic for animal pathogens since this metal is tightly bound by iron-sequestering proteins in the host[59,60]. Conversely, excess iron leads to cellular damage through the generation of reactive oxygen species (ROS) by the Fenton reaction. An exhaustive search for potential orthologs of the iron acquisition systems of *A. fumigatus* and *C. albicans*[59,61,62] clearly indicated that *D. coniospora* relies on an *A. fumigatus*-like system to gain access to the iron reservoirs of its host (Table S18).

Reductive iron assimilation (RIA) is the primary mechanism of iron acquisition in the plant necrotroph *Ustilago maydis*[63], but plays only a backup role to SAIA in the human pathogen *A. fumigatus* and the plant necrotroph *Co. heterostrophus*[64]. Nevertheless, RIA may be important for *D. coniospora* as shown by the presence of orthologs for all essential elements of the pathway. However, simplification and contraction of the pathway is still evident. Thus, *D. coniospora* harbors only five putative ferric reductases (compared to 17 in *C. albicans* and 15 in *A. fumigatus*) whose transcription varies at different growth stages; and features only a single multicopper oxidase and a high-affinity iron permease each (versus 5 and 4, respectively, in *C. albicans*, Table S18), both with low transcription.

D. coniospora may also be able to utilize heme as its iron source. Iron acquisition from the blood of the host by the human pathogen *C. albicans* involves the Rbt5-like hemoglobin receptor family, consisting of five glycosylphosphatidylinositol (GPI)-anchored proteins with conserved CFEM domains. CFEM-domain proteins with weak similarity to Rbt5 are also present in Pezizomycotina, but only one Rbt5-like protein from *Paracoccidioides* spp. has

been functionally identified as a hemoglobin receptor[65]. Although three proteins similar to GPI-anchored CFEM domain-containing proteins are also present *D. coniospora* (Table S18), these are only distantly related to the functionally validated hemoglobin receptors of *C. albicans* or *Paracoccidioides* spp. Similarly, only distant relatives of the ferritin receptor ALS3 of *C. albicans*[66] are encoded in the *D. coniospora* genome. Thus, *D. coniospora* does not seem to have the ability to internalize heme proteins. Instead, *D. coniospora* may proteolitically degrade these proteins, then bind, internalize and degrade the heme to gain access to iron. While no obvious orthologs of the hemoglobin or ferritin receptors are present, the *D. coniospora* genome encodes a protein (DCS_00111, Table S18) similar to the Cig1 virulence factor of *Cryptococcus neoformans* that acts as a hemophore by binding and assisting the uptake of heme[67]. Similarly, just as all sequenced Hypocreales, *D. coniospora* also encodes a predicted membrane-bound ortholog of the *C. albicans* Hmx1 heme oxygenase that takes part in heme degradation. Thus, instead of internalizing receptor-bound hemoglobin or other heme-proteins from its prey, *D. coniospora* may import and degrade heme after this porphyrin has been liberated by extracellular proteases. This strategy is viable as the endoparasite prevents any leakage of nutrients from the nematode carcass and fully utilizes its nutrient resources during the infection process[68]. In human-pathogenic *C. albicans* and in other pathogens that infect macroscopic hosts, this strategy may not be efficient as heme liberated after digesting the heme proteins would simply be washed away in the bloodstream or diluted out in the host tissues. However, *D. coniospora* fully devours the internal contents of its microscopic prey, and seals the nematode carcass during the eruption of conidiophores to prevent leakage of internal materials to the environment[68]. Thus, liberated heme would

remain fully accessible to this fungus during the infection process.

Iron homeostasis is interconnected with stress responses, as well as morphological and sexual development and virulence[64]. Not surprisingly, iron uptake, storage and utilization are tightly controlled in pathogens like *A. fumigatus* and *Co. heterostrophus*[59,62,69]. As expected, an ortholog of the central element of this regulatory network, the GATA factor SreA that represses both SAIA and RIA[59,62] is also present in *D. conispora*.

Secondary metabolism

Most synthases/synthetases encoded in the *D. coniospora* genome were found to be transcribed in at least some of the growth phases examined (Fig. S15, Table S16A). DcPKS1, DcPKS2, and DcNRPS5 were highly transcribed during mycelial growth, but were down-regulated in other life stages. DcNRPS2 was highly expressed during nematode infection, while DcNRPS4 was overexpressed in conidia. DcPKS3, DcPKS5, DcPKS6 and DcNRPS7 showed low transcription levels in all growth stages.

The *D. coniospora* genome does not encode a canonical PKS-NRPS[7,70]. However, it encodes three NRPS-PKS. One of these is similar to the *Co. heterostrophus* NPS7/PKS24 (DCS_03296/DcNRPS-PKS3, Fig. S10). The other two NRPS-PKSs (DCS_04285/DcNRPS-PKS1 and DCS_06362/DcNRPS-PKS2) belong to a family of phylogenetically distinct NRPS-PKSs found only among Hypocrealeans (Figs. S10 and S11).

The *D. coniospora* genome harbors 34 A (adenylation) domains. Phylogenetic analysis segregates these A domains into two groups: 1. acyl-CoA ligases (18 A domains in *D.*

conispora for fatty acid ligases, 4-coumarate-CoA ligases, CPS1/DIP2-like proteins) and 2. NRPS- and NRPS-like proteins (16 A domains in *D. conispora*) (Fig. S11). The A domains of two NRPS-PKSs (DCS_04285 and DCS_06362) form a clade that is distinct from that of the prototypical ChNPS7[71]. Surprisingly, the KS (ketoacyl synthase) domains of these NRPS-PKS hybrids cluster with fatty acid synthases instead of PKSs. Thus, DcNRPS-PKS1 and 2, together with NRPS-PKSs from other fungi with identical domain structures, may be part of a not yet characterized synthetase family. The transcription levels of both of these hybrid synthases was modest during the growth conditions surveyed.

In addition to the highly conserved NRPSs for an aminoaldipate semialdehyde dehydrogenase (DcNRPS11) and for the coprogen and ferricrocin siderophores (DcNRPS9 and DcNRPS6, respectively), the *D. coniospora* genome also harbors an NRPS gene cluster (centered on DcNRPS7) that shows significant synteni with similar clusters in other Hypocrealean fungi (Fig. S16A). In spite of the sequence conservation, the transcription level of DcNRPS7 is moderate in the growth conditions tested. The conservation of the gene cluster that includes the DcNRPS11 aminoaldipate semialdehyde dehydrogenase (Fig. S16B), together with its stable transcription during all growth periods suggest that this cluster may serve a basic biological function. On the other hand, DcNRPS4 (Fig. S16C), DcNRPS8 and DcNRPS-PKS1 may have resulted from relatively recent acquisition events that may have taken place before the *D. coniospora* / *T. inflatum* divergence (Fig. S11).

Drechmerins: structure, biosynthesis and bioactivities

On synthetic media, *D. coniospora* produces a complex mixture of >20 closely related linear

non-ribosomal peptide analogues, with microheterogeneity introduced by amino acid substitutions at several variable positions in the peptide chain. We have tentatively assigned the trivial name "drechmerins" to this peptide family. Mass spectrometry (MS) and tandem mass spectrometry (MS/MS) analyses of partially purified analogues indicate that the drechmerins are related to the leucinostatins[72], helioferins[73], roseoferins[74] and the culicinins[75] which all feature an AHMOD ([2*S*,4*S*]-2-amino-6-hydroxy-4-methyl-8-oxodecanoic acid) and multiple AIB (α-aminoisobutyric acid) residues. MS data are also consistent with the presence of one residue of AMD (2-amino-4-methyldecanoic acid) in drechmerins, which has only been reported previously from the culicinins[75]. Despite the multiplicity of possible analogues, the drechmerin mixture is dominated by two major components. High resolution electrospray ionization (HRESI) MS data (Fig. S14) indicated a molecular formula of $C_{61}H_{111}N_{11}O_{12}$ for the more abundant of these (drechmerin A, obs. 1190.8485 for $[M+H]^+$; calcd. 1190.8492 for $C_{61}H_{112}N_{11}O_{12}$), and $C_{60}H_{109}N_{11}O_{12}$ for the less abundant major congener (drechmerin B, obs. 1176.8330 for $[M+H]^+$; calcd. 1176.8335 for $C_{60}H_{110}N_{11}O_{12}$). B- and Y-fragment ions[9] (Table S19, Fig. S14) in MS/MS spectra are consistent with central core sequences of *N*-term–AHMOD–AIB–AIB–AMD–LEU/ILE–AIB–AIB–*C*-term and *N*-term–AHMOD–ALA–AIB–AMD–LEU/ILE–AIB–AIB–*C*-term for the 1189 and 1175 Da congeners, respectively. Thus, the difference of 14 Da between the two main components is attributable to an ALA in drechmerin B substituting for the AIB closest to the *N*-terminus in drechmerin A. MS data and comparisons with similar known peptaibiotics suggest that ALA, VAL, or IVA (isovaline) may substitute for the AIB residues in some of the various other drechmerin congeners. Accurate mass data from HRESI-MS/MS also indicate that the amino

acid that acylates the first of the two AIBs near the *C*-terminus of drechmerins has an internal residual mass of 113.0837 (Fig. S14, Table S19), which is consistent with both LEU and ILE (calculated residual mass: 113.0841), but not with the near-isobaric hydroxyproline (calculated residual mass: 113.0477). Although MS data are not sufficient to fully solve the structure of the 182 Da ($C_9H_{16}NO_2$) *N*-terminal residue, by analogy to related fungal peptides this is likely to comprise either a methyl-proline acylated by a C4 acid or a PRO acylated by a C5 acid. Similarly, by analogy the *C*-terminal is likely to comprise a -alanine (3-amino propanoic acid) amidated by an unknown group with a molecular formula of $C_7H_{16}N_3O$, possibly an N^1-methylpropane-1,2-diamine residue such as the one described to occupy the *C*-terminus of leucinostatin B[76]. Accurate MS/MS data also indicate that the AHMOD units in the drechmerins undergo facile α-β dehydration of the keto-hydroxy moiety (Fig. S14, Table S19), as has been reported for the leucinostatins[76]. This presents an obstacle to purification as attempts to prevent this conversion, or to drive the dehydration to completion, have so far been unsuccessful. Work is ongoing to overcome this purification challenge or to find a stable derivative so the structures can be fully elucidated using high-resolution NMR and chemical degradation techniques.

We propose that the assembly of the nonproteinogenic amino acids AHMOD and AMD initiates with the synthesis of 4-methyldecanoic acid on DcPKS1, followed by C2 amination to yield AMD, or C2 amination and oxidation of C6 and C8 to yield AHMOD (Fig. 6B). AIB units may be synthesized by DCS_05098 and DCS_05099 that are orthologous to the AIB synthetic genes TqaM and TqaL, respectively, from *Neurospora crassa*[77]. These two putative AIB-synthetic genes are not part of the main peptaibiotic biosynthetic cluster, but are

located at a different genomic locus on the same chromosome. The free-standing AT encoded by DcPKS2 may load the fatty acyl *N*-terminal cap onto the first T domain of DcNRPS1, initiating the assembly of a 10-amino acid peptide, likely terminating in an alanine residue. Release of the peptide may be catalyzed by the reductive release(R) domain of DcNRPS1 with a reduction/amination sequence (or by reductive amination) that may generate the *C*-terminal capping group and yield the 9-amino-acid peptaibiotics drechmerins. The observed microheterogeneity in the drechmerin congener mixture produced by the fungus would then reflect the relaxed substrate specificities of the corresponding A domains in DcNRPS1.

Peptaibiotics are ionophore antibiotics[78]. Drechmerin-containing crude organic extracts produced clear zones of inhibition against *Staphylococcus aureus* when tested at 100 μg, but showed no antibiotic activity against Enterococci or Gram-negative bacteria such as *E. coli*. These extracts also yielded a clear 6-mm inhibition zone against the corn leaf spot fungus (*Helminthosporium sativum*) when evaluated at 40 μg in a disk diffusion assay, with the most active purified fraction inhibiting growth at doses as low as 1.25 μg. Most importantly, we observed a potent and dose-dependent nematicidal activity of the drechmerin-containing crude dichloromethane culture broth extracts against the root lesion nematode (*Pratylenchus penetrans*) and the golden nematode (*Globodera rostochiensis*) (>70% mortality at 1mg/ml, and >50% mortality at 500 μg/ml, scored 48 hr post-application). Initial fractionation and bioassay studies indicated that the antifungal activity consistently co-purified with the nematicidal activities.

Definition of orthologous groups

We performed an all-by-all BLAST search of the 250,458 proteins predicted in our selection of 24 fungal species to identify homologs. We used blastp with the default settings, except that the maximum number of alignments was set to 10,000 rather than 250[79]. We defined the network of homologous proteins by connecting proteins for which the alignments returned by BLAST spanned more than 50% of both the query and the subject sequence (in order to avoid chimeric families due to fusion proteins) and had an E value < 1e-5. The weight of an edge in this network corresponds to the relative Smith-Waterman score (SW score) that is the total SW score of the query and the subject, divided by the total SW score of the query with itself. The total SW score is defined as the sum of SW scores of individual, non-overlapping alignments returned by BLAST for a query-subject pair. This results in two weights per pair (depending on which protein of the pair was the query). To obtain an undirected network suitable for mcl analyses we kept the lowest weight of the two. Furthermore, all edges with a weight < 0.2 were removed from the network. The network was clustered into families using mcl with default settings and the inflation parameter I set to 1.2[80]. This resulted in 19426 families.

For each of the 10,177 clusters cluster that consisted of more than three sequences we constructed and trimmed a multiple sequence alignment (MSA, Clustal Omega and trimAL –gappyout)[81,82]. Despite the overlap criterion applied when parsing our BLAST results we found four families in which fusion proteins ‘bridged’ non-homologous proteins. We split them up manually, so that at least part of each protein in a cluster is homologous to at least part of every other protein in the cluster. This resulted in 10,180 MSAs. For each of these MSAs, we inferred a phylogenetic tree with RaxML (-m PROTGAMMAIWAG -f a),

rooted this tree using midpoint rooting and predicted duplication events using the Species Overlap algorithm implemented in ete2 as this was shown to lead to more sensitive orthology predictions than strict tree reconciliation[83]. We split up families into distinct orthologous groups if we predicted duplication events to have occurred in the last common ancestor of our set of 24 species based on the species compositions in the tree, assuming that no horizontal transfer occurred. Using this approach we arrived at 20,655 orthologous groups, of which 11,291 had an associated gene tree.

References

1 Hane, J. K. & Oliver, R. P. RIPCAL: a tool for alignment-based analysis of repeat-induced point mutations in fungal genomic sequences. **9**, doi:10.1186/1471-2105-9-478 (2008).

2 Gao, Q. A. *et al.* Genome sequencing and comparative transcriptomics of the model entomopathogenic fungi *Metarhizium anisopliae* and *M. acridum*. *PLoS Genet.* **7**, 18, doi:e100126410.1371/journal.pgen.1001264 (2011).

3 Zhang, Y. *et al.* Specific adaptation of Ustilaginoidea virens in occupying host florets revealed by comparative and functional genomics. **5**, doi:10.1038/ncomms4849 (2014).

4 Muller, J. *et al.* eggNOG v2.0: extending the evolutionary genealogy of genes with enhanced non-supervised orthologous groups, species and functional annotations. **38**, D190-D195, doi:10.1093/nar/gkp951 (2010).

5 Winnenburg, R. *et al.* PHI-base: a new database for pathogen host interactions. *Nucleic acids research* **34**, D459-464, doi:10.1093/nar/gkj047 (2006).

6 Winnenburg, R. *et al.* PHI-base update: additions to the pathogen-host interaction database. *Nucleic Acids Res* **36**, D572-D576, doi:10.1093/nar/gkm858 (2008).

7 Bushley, K. E. & Turgeon, B. G. Phylogenomics reveals subfamilies of fungal nonribosomal peptide synthetases and their evolutionary relationships. *BMC Evol Biol* **10**, doi:10.1186/1471-2148-10-26 (2010).

8 Shimodaira, H. & Hasegawa, M. *Multiple Comparisons of Log-Likelihoods with Applications to Phylogenetic Inference*.

9 Roepstorff, P. & Fohlman, J. Proposal for a common nomenclature for sequence ions in mass spectra of peptides. *Biomedical mass spectrometry* **11**, 601, doi:10.1002/bms.1200111109 (1984).

10 Lai, Y. L. *et al.* Comparative genomics and transcriptomics analyses reveal divergent lifestyle features of nematode endoparasitic fungus *Hirsutella minnesotensis*. *Genome Biol. Evol.* **6**,

3077-3093, doi:10.1093/gbe/evu241 (2014).

11 Bushley, K. E. *et al.* The genome of *Tolypocladium inflatum*: Evolution, organization, and expression of the cyclosporin biosynthetic gene cluster. *PLoS Genet* **9**, doi:10.1371/journal.pgen.1003496 (2013).

12 Ward, E. *et al.* The Pochonia chlamydosporia Serine Protease Gene vcp1 Is Subject to Regulation by Carbon, Nitrogen and pH: Implications for Nematode Biocontrol. *Plos One* **7**, doi:10.1371/journal.pone.0035657 (2012).

13 Yang, J. K. *et al.* Genomic and proteomic analyses of the fungus *Arthrobotrys oligospora* provide insights into nematode-trap formation. *Plos Pathog* **7**, e1002179, doi:10.1371/journal.ppat.1002179 (2011).

14 Xiao, G. *et al.* Genomic perspectives on the evolution of fungal entomopathogenicity in *Beauveria bassiana*. **2**, 483, doi:10.1038/srep00483 (2012).

15 Schardl, C. L. *et al.* Plant-symbiotic fungi as chemical engineers: multi-genome analysis of the clavicipitaceae reveals dynamics of alkaloid loci. *PLoS Genet.* **9**, e1003323, doi:10.1371/journal.pgen.1003323 (2013).

16 Zheng, P. *et al.* Genome sequence of the insect pathogenic fungus Cordyceps militaris, a valued traditional Chinese medicine. *Genome Biol.* **12**, R116, doi:10.1186/gb-2011-12-11-r116 (2011).

17 Loftus, B. J. *et al.* The genome of the basidiomycetous yeast and human pathogen Cryptococcus neoformans. *Science (New York, N.Y.)* **307**, 1321-1324, doi:10.1126/science.1103773 (2005).

18 Ma, L. J. *et al.* Comparative genomics reveals mobile pathogenicity chromosomes in Fusarium. *Nature* **464**, 367-373, doi:10.1038/nature08850 (2010).

19 Dean, R. A. *et al.* The genome sequence of the rice blast fungus Magnaporthe grisea. **434**, 980-986, doi:10.1038/nature03449 (2005).

20 Pattemore, J. A. *et al.* The genome sequence of the biocontrol fungus Metarhizium anisopliae and comparative genomics of Metarhizium species. **15**, 660, doi:10.1186/1471-2164-15-660 (2014).

21 Meerupati, T. *et al.* Genomic mechanisms accounting for the adaptation to parasitism in nematode-trapping fungi. *PLoS Genet.* **9**, 20, doi:10.1371/journal.pgen.1003909 (2013).

22 Coleman, J. J. *et al.* The genome of Nectria haematococca: contribution of supernumerary chromosomes to gene expansion. *PLoS Genet.* **5**, e1000618, doi:10.1371/journal.pgen.1000618 (2009).

23 Hu, X. *et al.* Genome survey uncovers the secrets of sex and lifestyle in caterpillar fungus. *Chin. Sci. Bull.* **58**, 2846-2854, doi:10.1007/s11434-013-5929-5 (2013).

24 Larriba, E. *et al.* Sequencing and functional analysis of the genome of a nematode egg-parasitic fungus, *Pochonia chlamydosporia*. *Fungal genetics and biology : FG & B* **65**, 69-80, doi:http://dx.doi.org/10.1016/j.fgb.2014.02.002 (2014).

25 Clutterbuck, A. J. Genomic evidence of repeat-induced point mutation (RIP) in filamentous ascomycetes. *Fungal Genet. Biol.* **48**, 306-326, doi:10.1016/j.fgb.2010.09.002 (2011).

26 Treu, L. *et al.* The impact of genomic variability on gene expression in environmental Saccharomyces cerevisiae strains. *Environmental microbiology* **16**, 1378-1397, doi:10.1111/1462-2920.12327 (2014).

27 Martinez, D. *et al.* Genome sequencing and analysis of the biomass-degrading fungus

Trichoderma reesei (syn. Hypocrea jecorina). **26**, 553-560, doi:10.1038/nbt1403 (2008).
28 Daboussi, M. J. & Capy, P. Transposable elements in filamentous fungi. *Annu. Rev. Microbiol.* **57**, 275-299, doi:10.1146/annurev.micro.57.030502.091029 (2003).
29 Cuomo, C. A. *et al.* The Fusarium graminearum genome reveals a link between localized polymorphism and pathogen specialization. **317**, 1400-1402, doi:10.1126/science.1143708 (2007).
30 Ikeda, K. *et al.* Repeat-induced point mutation (RIP) in Magnaporthe grisea: implications for its sexual cycle in the natural field context. *Mol. Microbiol.* **45**, 1355-1364, doi:10.1046/j.1365-2958.2002.03101.x (2002).
31 Kasbekar, D. P. *Neurospora Duplications, and Genome Defence by RIP and Meiotic Silencing*. (Caister Academic Press, 2013).
32 Freitag, M., Williams, R. L., Kothe, G. O. & Selker, E. U. A cytosine methyltransferase homologue is essential for repeat-induced point mutation in Neurospora crassa. **99**, 8802-8807, doi:10.1073/pnas.132212899 (2002).
33 Lee, D. W., Freitag, M., Selker, E. U. & Aramayo, R. A Cytosine Methyltransferase Homologue Is Essential for Sexual Development in Aspergillus nidulans. **3**, doi:10.1371/journal.pone.0002531 (2008).
34 Braumann, I., van den Berg, M. & Kempken, F. Repeat induced point mutation in two asexual fungi, Aspergillus niger and Penicillium chrysogenum. *Curr Genet* **53**, 287-297, doi:10.1007/s00294-008-0185-y (2008).
35 Li, G., Zhang, K., Xu, J., Dong, J. & Liu, Y. Nematicidal Substances from Fungi. **1**, 212-233, doi:10.2174/187220807782330165 (2007).
36 Vandenboogert, P., Dijksterhuis, J., Velvis, H. & Veenhuis, M. Adhesive knob formation by conidia of the nematophagous fungus *Drechmeria coniospora*. *ANTON LEEUW INT J G* **61**, 221-229 (1992).
37 Son, H. *et al.* AbaA Regulates Conidiogenesis in the Ascomycete Fungus Fusarium graminearum. **8**, doi:10.1371/journal.pone.0072915 (2013).
38 Son, H. *et al.* WetA is required for conidiogenesis and conidium maturation in the Ascomycete fungus *Fusarium graminearum*. *Eukaryot. Cell* **13**, 87-98, doi:10.1128/ec.00220-13 (2014).
39 Park, H. S. & Yu, J. H. Genetic control of asexual sporulation in filamentous fungi. *Curr Opin Microbiol* **15**, 669-677, doi:10.1016/j.mib.2012.09.006 (2012).
40 Houbraken, J. & Samson, R. A. Phylogeny of Penicillium and the segregation of Trichocomaceae into three families. 1-51, doi:10.3114/sim.2011.70.01 (2011).
41 Adams, T. H., Wieser, J. K. & Yu, J. H. Asexual sporulation in Aspergillus nidulans. **62**, 35-+ (1998).
42 Miskei, M., Karányi, Z. & Pócsi, I. Annotation of stress–response proteins in the aspergilli. *Fungal genetics and biology : FG & B* **46**, S105-S120, doi:http://dx.doi.org/10.1016/j.fgb.2008.07.013 (2009).
43 Bayram, O. & Braus, G. H. Coordination of secondary metabolism and development in fungi: the velvet family of regulatory proteins. *Fems Microbiol. Rev.* **36**, 1-24, doi:10.1111/j.1574-6976.2011.00285.x (2012).
44 Bayram, O. *et al.* VelB/VeA/LaeA complex coordinates light signal with fungal development and secondary metabolism. *Science (New York, N.Y.)* **320**, 1504-1506, doi:10.1126/science.1155888 (2008).

45 Kim, H.-K. *et al.* Functional Roles of FgLaeA in Controlling Secondary Metabolism, Sexual Development, and Virulence in Fusarium graminearum. **8**, e68441, doi:10.1371/journal.pone.0068441 (2013).

46 Wiemann, P. *et al.* FfVel1 and FfLae1, components of a velvet-like complex in Fusarium fujikuroi, affect differentiation, secondary metabolism and virulence. 10.1111/j.1365-2958.2010.07263.x, doi:10.1111/j.1365-2958.2010.07263.x (2010).

47 Butchko, R. A. E., Brown, D. W., Busman, M., Tudzynski, B. & Wiemann, P. Lae1 regulates expression of multiple secondary metabolite gene clusters in Fusarium verticillioides. **49**, 602-612, doi:http://dx.doi.org/10.1016/j.fgb.2012.06.003 (2012).

48 López-Berges, M. S. *et al.* The velvet complex governs mycotoxin production and virulence of Fusarium oxysporum on plant and mammalian hosts. **87**, 49-65, doi:10.1111/mmi.12082 (2013).

49 Djamei, A. *et al.* Metabolic priming by a secreted fungal effector. *Nature* **478**, 395-+, doi:10.1038/nature10454 (2011).

50 Guyon, K., Balague, C., Roby, D. & Raffaele, S. Secretome analysis reveals effector candidates associated with broad host range necrotrophy in the fungal plant pathogen Sclerotinia sclerotiorum. *BMC Genomics* **15**, 18, doi:10.1186/1471-2164-15-336 (2014).

51 Rouxel, T. *et al.* Effector diversification within compartments of the Leptosphaeria maculans genome affected by Repeat-Induced Point mutations. *Nat. Commun.* **2**, 10, doi:10.1038/ncomms1189 (2011).

52 Beys-da-Silva, W. O. *et al.* Secretome of the Biocontrol Agent Metarhizium anisopliae Induced by the Cuticle of the Cotton Pest Dysdercus peruvianus Reveals New Insights into Infection. *J. Proteome Res.* **13**, 2282-2296, doi:10.1021/pr401204y (2014).

53 Merzendorfer, H. Chitin synthesis inhibitors: old molecules and new developments. **20**, 121-138, doi:10.1111/j.1744-7917.2012.01535.x (2013).

54 Frand, A. R., Russel, S. & Ruvkun, G. Functional genomic analysis of C. elegans molting. *PLoS biology* **3**, e312, doi:10.1371/journal.pbio.0030312 (2005).

55 Dijksterhuis, J., Veenhuis, M. & Harder, W. Ultrastructural study of adhesion and initial stages of infection of nematodes by conidia of *Drechmeria coniospora*. *Mycol. Res.* **94**, 1-8 (1990).

56 Jansson, H. B. & Friman, E. Infection-related surface proteins on conidia of the nematophagous fungus *Drechmeria coniospora*. *Mycol Res* **103**, 249-256, doi:10.1017/s0953756298007084 (1999).

57 Chen, W. *et al.* Fungal Cytochrome P450 Monooxygenases: Their Distribution, Structure, Functions, Family Expansion, and Evolutionary Origin. **6**, 1620-1634, doi:10.1093/gbe/evu132 (2014).

58 Pedrini, N., Zhang, S., Juárez, M. P. & Keyhani, N. O. Molecular characterization and expression analysis of a suite of cytochrome P450 enzymes implicated in insect hydrocarbon degradation in the entomopathogenic fungus Beauveria bassiana. **156**, 2549-2557, doi:10.1099/mic.0.039735-0 (2010).

59 Schrettl, M. & Haas, H. Iron homeostasis-Achilles' heel of *Aspergillus fumigatus*? *Curr. Opin. Microbiol.* **14**, 400-405, doi:10.1016/j.mib.2011.06.002 (2011).

60 Anderson, C. P. & Leibold, E. A. Mechanisms of iron metabolism in Caenorhabditis elegans. **5**, 113, doi:10.3389/fphar.2014.00113 (2014).

61 Chen, C. B., Pande, K., French, S. D., Tuch, B. B. & Noble, S. M. An Iron Homeostasis

Regulatory Circuit with Reciprocal Roles in Candida albicans Commensalism and Pathogenesis. *Cell Host Microbe* **10**, 118-135, doi:10.1016/j.chom.2011.07.005 (2011).

62 Haas, H. Iron - a key nexus in the virulence of Aspergillus fumigatus. *Front. Microbiol.* **3**, 10, doi:10.3389/fmicb.2012.00028 (2012).

63 Mei, B. G., Budde, A. D. & Leong, S. A. sid1, a gene initiating siderophore biosynthesis in Ustilago maydis: molecular characterization, regulation by iron, and role in phytopathogenicity. *Proc. Natl. Acad. Sci. U. S. A.* **90**, 903-907, doi:10.1073/pnas.90.3.903 (1993).

64 Condon, B. J., Oide, S., Gibson, D. M., Krasnoff, S. B. & Turgeon, B. G. Reductive iron assimilation and intracellular siderophores assist extracellular siderophore-driven iron homeostasis and virulence. *Molecular plant-microbe interactions : MPMI* **27**, 793-808, doi:10.1094/mpmi-11-13-0328-r (2014).

65 Bailao, E. F. *et al.* Hemoglobin uptake by Paracoccidioides spp. is receptor-mediated. *PLoS neglected tropical diseases* **8**, e2856, doi:10.1371/journal.pntd.0002856 (2014).

66 Almeida, R. S. *et al.* the hyphal-associated adhesin and invasin Als3 of Candida albicans mediates iron acquisition from host ferritin. *PLoS pathogens* **4**, e1000217, doi:10.1371/journal.ppat.1000217 (2008).

67 Cadieux, B. *et al.* The Mannoprotein Cig1 Supports Iron Acquisition From Heme and Virulence in the Pathogenic Fungus Cryptococcus neoformans. **207**, 1339-1347, doi:10.1093/infdis/jit029 (2013).

68 Dijksterhuis, J., Harder, W., Wyss, U. & Veenhuis, M. Colonization and digestion of nematodes by the endoparasitic nematophagous fungus *Drechmeria coniospora*. *Mycol. Res.* **95**, 873-878 (1991).

69 Zhang, N. *et al.* Iron, Oxidative Stress, and Virulence: Roles of Iron-Sensitive Transcription Factor Sre1 and the Redox Sensor ChAp1 in the Maize Pathogen Cochliobolus heterostrophus. **26**, 1473-1485, doi:10.1094/mpmi-02-13-0055-r (2013).

70 Fisch, K. M. Biosynthesis of natural products by microbial iterative hybrid PKS-NRPS. *RSC Adv.* **3**, 18228-18247, doi:10.1039/c3ra42661k (2013).

71 Lee, B. N. *et al.* Functional analysis of all nonribosomal peptide synthetases in Cochliobolus heterostrophus reveals a factor, NPS6, involved in virulence and resistance to oxidative stress. *Eukaryot. Cell* **4**, 545-555, doi:10.1128/ec.4.3.545-555.2005 (2005).

72 Isogai, A. *et al.* Structural analysis of new syringopeptins by tandem mass spectrometry. **59**, 1374-1376 (1995).

73 Gräfe, U. *et al.* Helioferins; novel antifungal lipopeptides from Mycogone rosea: Screening, isolation, structures and biological properties. **48**, 126-133 (1995).

74 Degenkolb, T. *et al.* Roseoferin, a new aminolipopeptide antibiotic complex from *Mycogone rosea*. **53**, 184-190 (2000).

75 He, H. Y. *et al.* Culicinin D, an antitumor peptaibol produced by the fungus Culicinomyces clavisporus, strain LL-12I252. **69**, 736-741, doi:10.1021/np058133r (2006).

76 Isogai, A., Nakayama, J., Takayama, S., Kusai, A. & Suzuki, A. Structural elucidation of minor components of peptidyl antibiotic P168s (leucinostatins) by tandem mass spectrometry. *Bioscience, biotechnology, and biochemistry* **56**, 1079-1085 (1992).

77 Gao, X. *et al.* Fungal Indole Alkaloid Biosynthesis: Genetic and Biochemical Investigation of the Tryptoquialanine Pathway in Penicillium aethiopicum. **133**, 2729-2741,

doi:10.1021/ja1101085 (2011).

78 Stoppacher, N. *et al.* The comprehensive peptaibiotics database. *Chem. Biodivers.* **10**, 734-743, doi:10.1002/cbdv.201200427 (2013).

79 Altschul, S. F., Gish, W., Miller, W., Myers, E. W. & Lipman, D. J. Basic local alignment search tool. *J Mol Biol* **215**, 403-410, doi:10.1016/s0022-2836(05)80360-2 (1990).

80 Enright, A. J., Van Dongen, S. & Ouzounis, C. A. An efficient algorithm for large-scale detection of protein families. *Nucleic Acids Res* **30**, 1575-1584 (2002).

81 Sievers, F. & Higgins, D. G. Clustal Omega, accurate alignment of very large numbers of sequences. *Methods in molecular biology (Clifton, N.J.)* **1079**, 105-116, doi:10.1007/978-1-62703-646-7_6 (2014).

82 Capella-Gutierrez, S., Silla-Martinez, J. M. & Gabaldon, T. trimAl: a tool for automated alignment trimming in large-scale phylogenetic analyses. *Bioinformatics* **25**, 1972-1973, doi:10.1093/bioinformatics/btp348 (2009).

83 Marcet-Houben, M. & Gabaldon, T. The tree versus the forest: the fungal tree of life and the topological diversity within the yeast phylome. *PloS One* **4**, e4357, doi:10.1371/journal.pone.0004357 (2009).
